# Molecular and Cellular Profiling of Scalp Psoriasis Reveals Differences and Similarities Compared to Skin Psoriasis

**DOI:** 10.1371/journal.pone.0148450

**Published:** 2016-02-05

**Authors:** Juan Ruano, Mayte Suárez-Fariñas, Avner Shemer, Margeaux Oliva, Emma Guttman-Yassky, James G. Krueger

**Affiliations:** 1 Department of Dermatology, Reina Sofia University Hospital, Cordoba, Spain; 2 Instituto Maimonides de Investigacion Biomedica de Cordoba (IMIBIC)/Hospital Universitario Reina Sofia/Universidad de Cordoba, Cordoba, Spain; 3 Department of Dermatology, Icahn School of Medicine at Mount Sinai, New York, New York, United States of America; 4 Department of Population Health Science and Policy, Icahn School of Medicine at Mount Sinai, New York, New York, United States of America; 5 Department of Genetics and Genomics Science, Icahn School of Medicine at Mount Sinai, New York, New York, United States of America; 6 Department of Dermatology, Tel-Hashomer Hospital and Tel-Aviv University, Tel-Aviv, Israel; 7 Icahn School of Medicine at Mount Sinai, New York, New York, United States of America; 8 Laboratory for Investigative Dermatology, The Rockefeller University, New York, New York, United States of America; CNRS-University of Toulouse, FRANCE

## Abstract

Scalp psoriasis shows a variable clinical spectrum and in many cases poses a great therapeutic challenge. However, it remains unknown whether the immune response of scalp psoriasis differs from understood pathomechanisms of psoriasis in other skin areas. We sought to determine the cellular and molecular phenotype of scalp psoriasis by performing a comparative analysis of scalp and skin using lesional and nonlesional samples from 20 Caucasian subjects with untreated moderate to severe psoriasis and significant scalp involvement and 10 control subjects without psoriasis. Our results suggest that even in the scalp, psoriasis is a disease of the inter-follicular skin. The immune mechanisms that mediate scalp psoriasis were found to be similar to those involved in skin psoriasis. However, the magnitude of dysregulation, number of differentially expressed genes, and enrichment of the psoriatic genomic fingerprint were more prominent in skin lesions. Furthermore, the scalp transcriptome showed increased modulation of several gene-sets, particularly those induced by interferon-gamma, compared with that of skin psoriasis, which was mainly associated with activation of TNF*α*/L-17/IL-22-induced keratinocyte response genes. We also detected differences in expression of gene-sets involving negative regulation, epigenetic regulation, epidermal differentiation, and dendritic cell or Th1/Th17/Th22-related T-cell processes.

## Introduction

Psoriasis is a chronic inflammatory skin disease affecting 2–3% of the population. Involvement of the scalp frequently occurs (in 50% to 100% of patients) and can be the only disease manifestation [1, 2]. The clinical features of scalp psoriasis vary from intermittent mild, patchy, red, scaly lesions to total scalp involvement.

The treatment of scalp psoriasis is only partially successful, due to limited available topical treatments and the reduced efficacy of some systemic treatments. Current clinical guidelines suggest the use of systemic therapies only for patients with severe and recalcitrant disease [3–6]. Systemic approaches used in patients with widespread disease include phototherapy, cyclosporine, acitretin, methotrexate, retinoids, biologic therapies, and small molecules such as apremilast [2]. However, few of these therapies have been evaluated specifically for scalp psoriasis.

While there is a relatively large number of murine models of psoriasis, only a few studies have examined the pathomechanisms of scalp psoriasis [7]. Because of the abundance of hair follicles in mice, murine models may initially seem like an ideal model for scalp psoriasis. However, unlike outcomes in murine models, scalp psoriasis does not generally result in hair loss. Anatomical and physiological differences between mouse and human skin explain this clinical difference. Namely, when psoriasis affects the skin of mice, which is disproportionately composed of densely distributed hair follicles, the infundibular and low follicular portions are entirely affected by an intense inflammatory infiltrate. This finding may be due to the short inter-follicular regions that do not contain any rete ridges in mice [7].

We thus sought to compare whether scalp psoriasis shares the same biological mechanisms as psoriasis in other skin areas.

## Materials and Methods

### Patients and samples isolation

Skin punch biopsies (6 mm diameter) were obtained from 20 Caucasian patients (16 males, ages 26–58 years, median 38) with moderate to severe psoriasis (involvement of >10% body surface area and/or PASI >10) with significant scalp involvement that did not receive any therapy for >4 weeks and 10 control subjects without psoriasis (4 males, ages 24–69, median 41). Lesional (LS) samples were isolated from the infiltrated border of a psoriatic plaque. Non lesional (NL) samples were taken from scalp areas with no visible psoriasis between the infiltrated plaques (Figure A in S1 File). This study was conducted according to the principles expressed in the Declaration of Helsinki and informed consent for their information to be stored in the hospital database and used for research was obtained from all subjects in written form. This study protocol was approved by Institutional Review Boards of The Rockefeller University (New York, USA), Tel-Hashomer Hospital (Tel-Aviv, Israel), and Haukeland University Hospital (Bergen, Norway).

### Immunohistochemistry

Frozen skin sections were stained in a standard manner. To determine the extent of inflammatory infiltration in interfollicular (IFL) and infundibular (inFD) areas of the scalp, we analyzed the differences in cell density between LS and NL samples. For each of the anatomical areas, at least 50% of the patients were evaluated. Primary antibodies for immunohistochemistry are listed in Table A in S1 File. Regarding inFD, the cell count was given in cells per mm2 of the infundibular epithelium. The positive cells in an area, 70 microns outside the infundibulum, were counted and presented as cells per mm epithelial length. Similarly, the cells within and outside the lower part of the follicle (LF), below the sebaceous duct and above the papilla, were enumerated. No evaluation of the cells within or outside the follicular papillae was done due to too shallow biopsies (Figure A in S1 File). Immunohistochemistry was conducted in batches for paired samples and representative staining is shown.

### Quantitative Real-time—PCR (RT-PCR)

To determine the cytokine environment of the scalp biopsies, we measured mRNA expression of major Th1-, Th2-, Th17- and Th22-related cytokines by real-time PCR (RT-PCR). RT-PCR was performed using Taqman gene expression assay. We quantified the expression of genes coding IFN-γ, IL-4, IL-17, IL-22, p19 (subunit of IL-23), p40 (subunit of IL-12 and IL-23), IL-20 and iNOS by RT-PCR using Taqman 16-gene low-density array cards (Applied Biosystems, Foster City, CA). The resulting data were normalized to human hARP gene expression as a housekeeping gene (Applied Biosystems, Foster City, CA).

### Gene expression by microarrays

RNA was extracted from skin biopsies using Qiagen RNeasy Fibrous Tissue Mini Kit (QIAGEN, Valencia, CA) and later hybridized to HGU133Plus 2.0 chip (Affymetrix, Santa Clara, CA). Scanned Affymetrix chips were scrutinized for spatial artifacts using Harslight package [8]. Differences in expression between groups were assessed using mixed effect models with areas (skin or scalp) and samples (LS, NL and N) as fixed effects and as a random intercept for each patient. Direct comparisons of LS (or NL) between regions was adjusted by area and estimated in the framework of limma R package. Transcripts with low expression were excluded. P-values were adjusted for multiple hypotheses using the Benjamini—Hochberg procedure. Genes showing fold-change (FCH) >2 and false discovery rate (FDR) <0.05 were considered to be part of the sample profile. Genomic expression differences between scalp and skin psoriasis samples were represented as heat maps, Venn diagrams and Principal Component Analysis (PCA). Raw data have been deposited in NCBI’s Gene Expression Omnibus (GEO) and are accessible through GEO Series accession number GSE75343.

We used LS and N skin samples from GSE27628 to study the expression of keratin and keratin associated proteins/KRTAPs genes from five psoriasis models (K5-Tie2, imiquimod, K14-AREG, K5-Stat3C and K5-TGFbeta1) and from GSE58558 and GSE32924 to study the expression of alopecia areata (AA) and atopic dermatitis (AD) in LS vs N samples in humans [9, 10].

### Statistical Analyses

The graphs were produced and the statistics were performed using several packages of the R language [11]. Differences of cytokine mRNA expression among groups were evaluated using Kruskal-Wallis test followed by Dunn’s multiple comparison test. A p-value <0.05 was considered significant.

### Microarray analysis

#### Differentially expressed genes (DEGs)

Microarray gene expression analysis data were analyzed and graphs were produced using several packages of the R language [11]. Differences in expression profiles were assessed using mixed models (in the limma package framework) with fixed factors Area (Skin/Scalp) and Tissue (LS, NL, Normal) and a random intercept for each patient. The significance of each comparison was assessed via contrast using a moderated t-test and adjustment for multiple hypothesis using the Benjamini-Hochberg approach. GSEA and GSVA were performed using a manually curated gene-set database. Data generated from the gene-array analysis of murine psoriasis-related models were compared with human skin and scalp transcriptomes. A more detailed description of these computational approaches is available as a Supplementary Information file online.

#### Gene set enrichment analysis (GSEA)

To further consider the biological significance of our data, we used Gene Set Enrichment Analysis (GSEA) in the classical manner, to identify pathways that correlate with the skin and scalp psoriatic phenotype [12, 13]. For a curated collection of psoriasis and immune-related gene sets, GSEA was used to identify sets enriched in the phenotype defined by the LS vs NL transcriptome and LS vs N transcriptome for both skin and scalp. The significance (p-value) for the observed enrichment score (ES) was calculated using 1000 simulations [14]. The ES reflects the degree to which a gene set is overrepresented at the top or bottom of a ranked list of genes (here the fold change of LS vs NL or LS vs Healthy) and it’s presented in its normalized form to account for differences in gene set size, thus allowing for a comparison of the enrichment magnitude across variable-sized gene sets. A positive ES/NES indicates that the gene set is significantly enriched at the “top” off the ordered fold change list, i.e., the gene set is collectively up-regulated and a negative NES indicates that the list is enriched at the “bottom” off the list, i.e., the gene set tends to be down-regulated. A modified balloon graph was used to represent NES values (size), NES sign (direction) and p-value (color) for every gene set and phenotype comparison. The software GSEA (available at http://www.broadinstitute.org/gsea/) was used for this analysis.

#### Gene Set Variation Analysis (GSVA)

GSVA method was used to estimate variation of pathway activity in our sample population in a non-parametric unsupervised manner. GSVA performs a change in coordinate systems, transforming the data from a gene by sample matrix to a gene set by sample matrix, which allows for the evaluation of pathway enrichment for each sample. GSVA constitutes a starting point to build pathway-centric models of scalp and skin psoriasis biology [15]. The input for the GSVA algorithm is a gene expression matrix in the form of log2 microarray expression values. The output of the algorithm is a matrix containing pathway enrichment profile for each gene set and sample.

## Results

### T cell infiltrates and increased epidermal growth/differentiation are observed mainly in interfollicular areas of scalp psoriasis

**Fig 1:**
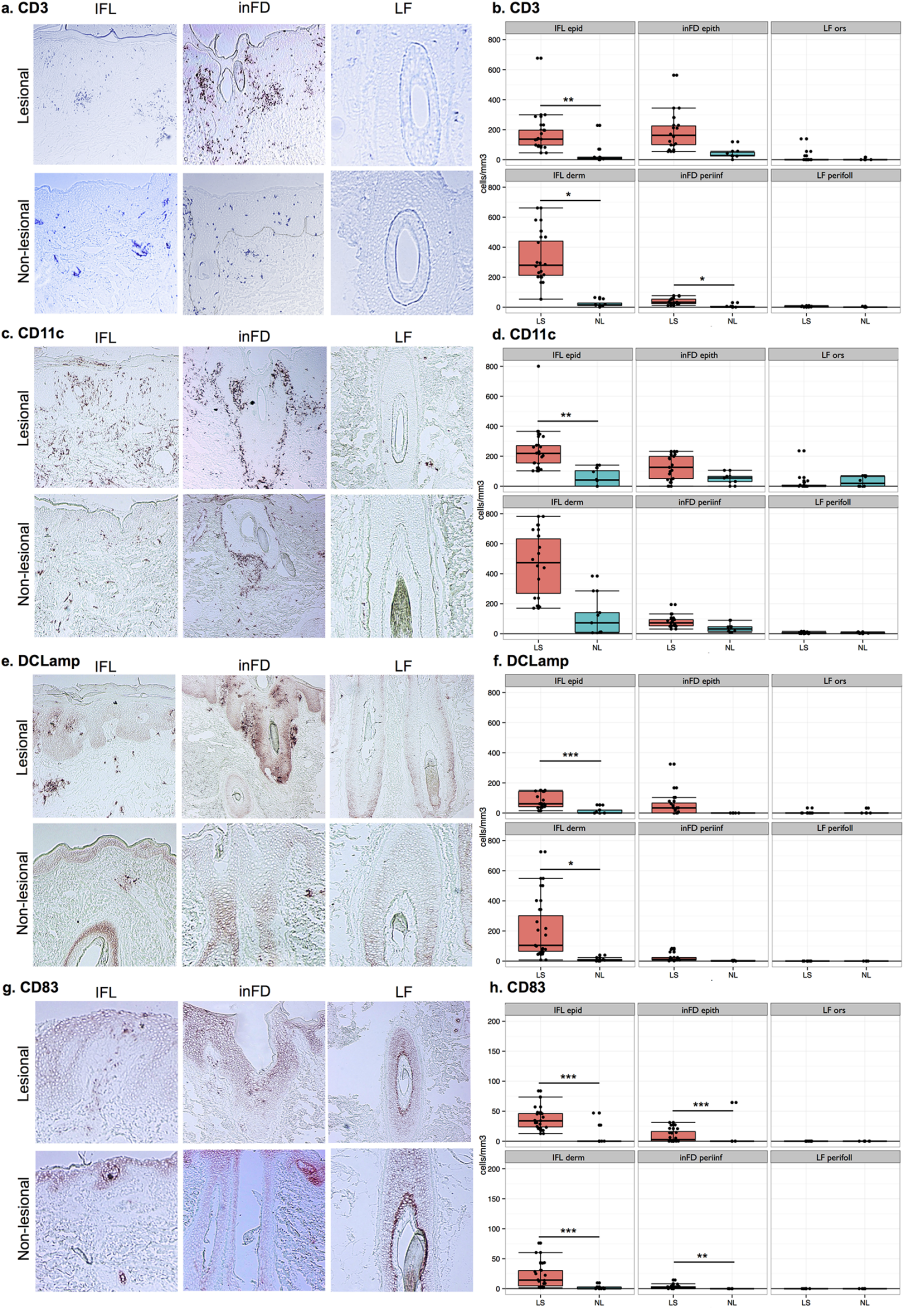
Distribution and number of CD3+, CD11c+, DC-Lamp+ and CD83+ cells in the various compartments of the study groups. Panels a, c, e and g show the histological pictures. Graphs b, d, f and h show the number of cells and statistical analysis. IFL, interfolicular; inFD, infundibular; LF, low follicule; Epid, epidermis; Epith, epithelium; Ors, outer root sheath; Periinf, periinfundibular area; Perifoll, perifollicular area. Cells were counted per mm2 epidermis and dermis in the IFL area. Median and ranges of log transformed values are given. Statistics: <0.05 = *; p<0.01 = **; p<0.001 = ***.

In this analysis, we sought to compare the hyperproliferative state of scalp psoriasis by measuring epidermal thickness and K16 staining on different scalp compartments: IFL epidermis, inFD epithelium, outer root sheath (ors) and LF (Figure A in S1 File). Genomic and protein expression of K16 have been used as markers of active psoriasis lesions and their expressions are down-regulated in resolving disease. The epidermal thickness and K16 staining pattern of scalp psoriasis were significantly higher in LS than NL biopsies in both IFL epidermis and inFD epithelium, with no differences observed in the ors or perifollicular area of the LF (Figure B(a-c) in S1 File). To determine the extent of mononuclear cell infiltration in IFL and inFD areas, we analyzed the differences in CD3^+^ and CD8^+^ cell counts between LS and NL samples. In the IFL area, CD3^+^ and CD8^+^ mononuclear cell numbers were significantly higher in both the epidermis and dermis when comparing LS and NL samples (Fig 1 and Table A in S1 File). However, in the inFD area, CD3^+^ infiltration was only observed in the peri-infundibular compartment of LS samples. Finally, the LF compartment showed minimal T-cell infiltration in the ors or perifollicular area.

### Small infiltrates of Dendritic cells (DCs) are localized to follicular scalp psoriasis

To determine the extent to which DCs contribute to scalp psoriasis, we performed immunohistochemistry for DC markers in different scalp compartments (Table 1). DC populations were mainly localized to the IFL compartment. Higher cellular infiltrates of myeloid DCs (CD11c^+^) and mature DCs (DC-LAMP^+^ and /CD68^+^) were observed in LS compared to NL samples. Similarly, Langerhans cells (LCs) (CD207/Langerin^+^) and resident DCs (BDCA-1^+^) showed higher counts in LS skin, where LCs are mostly found in the epidermis and BDCA-1^+^ counts are increased in the dermis. In inFD, peri-infundibular and LF compartments, DCs counts were low or absent, without significant differences between LS and NL samples. This finding is consistent with increased T cell infiltrates and epidermal growth in interfollicular areas.

**Table 1:** Density of dendritic cells and T cells identified with various markers in different areas and compartments of scalp.

		IFL		inFD		LF	
		Epidermis	Dermis	Epithelium	Periinf.	Ors	Perifoll.
*Marker*	*Samples*	*Cells*/*mm*^2^	*Cells*/*mm*^2^	*Cells*/*mm*^2^	*Cells*/*mm*^2^	*Cells*/*mm*^2^	*Cells*/*mm*^2^
*CD*8	LS	478 (5–343)*,^−^	433 (69–447)**, ^−^	297 (973–378)*,^−^	25 (12–60)	0 (0–62)	2 (0–13)
	NL	33 (0–488)^−^	43 (9–100)^−^	30 (0–242)^−^	8 (0–33)	0 (0)	2 (0–6)
	Normal	22 (0–36)	29 (10–55)	47 (0–104)	11 (0–45)	0 (0–26)	2 (0–13)
*BDCA*—1	LS	122 (10–207)^−^,*	148 (2–359)^−^,*	61 (0–169)	22 (1–169)	0 (0–19)	0 (0)
	NL	17 (7–94)^−^	31 (1–92)^−^	33 (0–134)	17 (5–56)	0 (0)	0 (0)
	Normal	61 (26–135)	29 (9–78)	62 (0–302)	9 (0–32)	0 (0–46)	0 (0–3)
*BDCA*—2	LS		342 (0–304)		25 (2–52)		5 (0–54)
	NL	n.c.^a^	95(25–399)**	n.c.	19(0–48)	n.c.	5(0–16)
	Normal		22 (0–49)		4 (0–44)		1 (0–13)
*CD*207	LS	232 (10–429)	30 (0–54)**,*	319 (17–349)	0 (0–10)	29 (0–158)	0 (0)
(*Langerin*)	NL	187 (72–628)	4 (0–16)^−^	429 (189–1414)	0 (0)	26 (0–130)	0 (0)
	Normal	338 (13–559)	0 (0–16)	182 (40–715)	0 (0–8)	19 (0–186)	0 (0–1)
*CD*68	LS	98 (6–178)**,**	1390 (344–1525)***,*,^−^	189 (0–571)	164 (23–161)**,^−^	0 (0–25)	26 (7–38)**,^-^
	NL	0 (0–60)^-^	245 (133–597)	0 (0)^−^	32 (15–92)^−^	0 (0–54)	13 (4–22)^−^
	Normal	0 (0–74)	150 (13–209)	cell5 row 9	cell6 row 9	cell7 row 9	cell8 row 9
*DC*205	LS	39 (7–60)	375 (40–494)**,**	14 (0–57)*,^−^	42 (9–78)	0 (0–5)	0 (0–7)
	NL	14 (0–29)	16 (2–173)^−^	0 (0–33)^−^	3 (2–55)	0 (0)	0 (0–2)
	Normal	23 (0–60)	45 (4–154)	0 (0)	18 (3–45)	0 (0)	0 (0)

Results are given as median and (range) values. IFL: interfollicular area; inFD: infundibular area; Lang.: Langerin; LF: lower follicle; n.c.: no cells; Periinf.: periinfundibular area; Ors: outer root sheeth; perifoll.: perifollicular area; LS: lesional scalp (LS); NL-B: samples from nonlesional scalp areas; N: scalp biopsies from healthy controls. Statistical comparisons and the order in which significant p-values are presented: LS vs N, LS vs NL, NL vs N. No indication or “—” = not significant;

* = p<0.05;

** = p<0.01;

*** = p<0.001.

### Similar features of scalp psoriasis transcriptome vs. psoriasis vulgaris (skin) transcriptome

**Fig 2:**
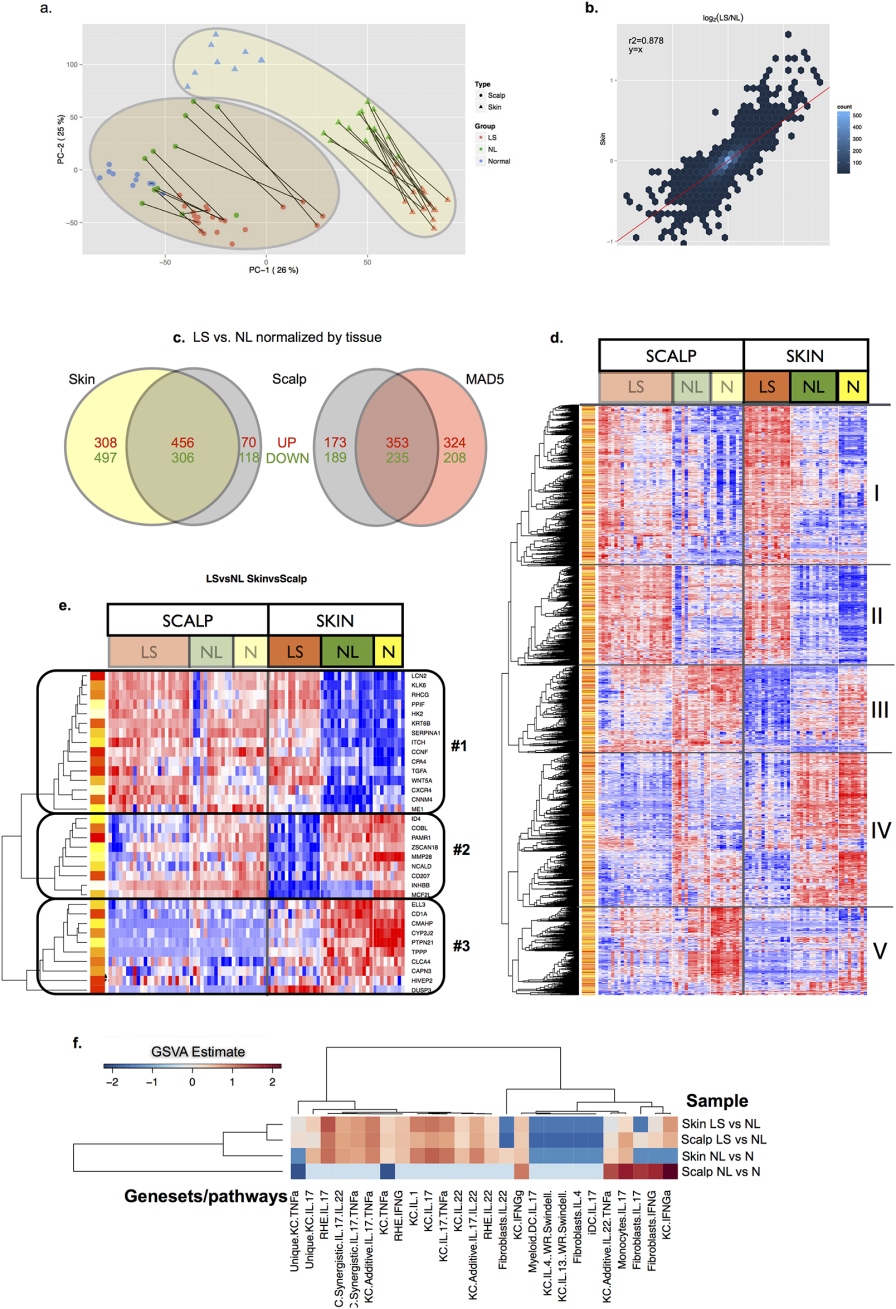
Descriptive analysis of scalp vs skin transcriptomes. (a) Principal component analysis (PCA) was performed to reveal the internal structure of the individual transcriptomes and to best explain the variance among samples when comparing the studied areas. In our data, PCA showed a better discrimination for skin samples than for scalp biopsies. (b) Scatter plot of log2FCH of LS vs NL DEGs on skin vs scalp transcriptomes. (c) Venn diagram of LS vs NL DEGs on scalp vs skin or MAD5 transcriptomes. (d) Heat map of genomic expression differences in scalp or skin psoriasis comparing LS, NL and N samples and (e) heat map showing three clusters of genes involved in differentiation of LS/NL samples in scalp vs skin areas. (f) Clustering heatmap of GSVA estimate values for a group of immune-related gene-sets comparing LS vs NL on scalp vs skin psoriasis. Terms of the X-axis refer to several set of genes up-regulated in cell culture by addition of cytokines. Unique: when a gene set is unique for that cell-cytokines combination, and no gene appears in other gene set. Synergistic: effect due to adding two cytokines to the cell culture at the same time. Additive: effect due to adding two cytokines to the cell culture sequentially. KC: Keratinocytes; RHE: Reconstituted Epidermis; DC: Dendritic Cells; iDC: inflammatory Dendritic Cells.

We used microarray to define the scalp psoriasis transcriptome and compared it with our published psoriasis skin transcriptome and the Meta-Analysis Derived (MAD) Transcriptome of Psoriasis 5 (MAD5), both sharing the same microarray platform [16]. PCA of microarray data confirms a clear separation into two different compartments, corresponding to scalp and skin areas (Fig 2a), with slightly greater expression of some genes in skin than in scalp samples as is shown in the scatterplot (Fig 2b). When evaluating the PCA plot, the difference between LS, NL and N samples was more evident for skin than scalp areas, indicating fewer differences between normal and scalp psoriasis compared with skin psoriasis. The total number of significant up- and down-regulated DEGs was somewhat higher in skin than scalp for both LS and NL samples (Fig 2c and Tables B-G in S1 File). The scalp psoriasis transcriptome DEG set contained 626 up-regulated probe sets (526 known unique DEGs) and 476 down-regulated probe sets (424 unique DEGs) (Fig 2d). Blocks I and II of Fig 2d contain genes which are largely up-regulated in LS samples compared to NL or N tissue samples, whereas blocks III, IV, and V contain genes that are mostly down-regulated in LS compared to controls. Genes most significantly up-regulated in scalp LS include S100A12, DEFB4, IL1F9 (coding IL-36γ), and IL8 (all >20-fold increased over scalp NL) and all genes that are regulated by IL-17. Additionally, many other genes that are highly represented in the skin psoriasis transcriptome, such as OASL, KYN4, PI3, CCL20, S100A9, LCN2, CXCL9, IFI27, and OAS1, are all up-regulated >5-fold in scalp LS. Several of these genes are also strongly regulated by IL-17 in keratinocytes (Table B in S1 File). Within gene comparisons, we found that background NL scalp of psoriasis had gene expressions that were intermediate between LS and N scalp, implying low levels of inflammation in this tissue. This is most clearly seen in block I and block V of Fig 2d. Further analysis of NL compared to N scalp gene expression using GSEA for cytokine-induced inflammatory pathways showed strong up-regulation of gene pathways regulated by TNF, IL-17, and interferons in NL scalp compared to N scalp (Fig 2f).

### Dissimilar features of the scalp transcriptome compared to psoriasis vulgaris (skin) transcriptome

We also detected some significant differences in the transcriptome of scalp psoriasis and psoriasis vulgaris, defined by fold-change differences between LS and NL or N tissue. Clustering algorithms identified 3 distinct clusters of these differences, as shown in Fig 2e. Genes that are strongly up-regulated in psoriasis vulgaris are shown in cluster 1 of Fig 2e, whereas genes that are down-regulated in LS skin psoriasis are shown in clusters 2 and 3 of Fig 2e. In scalp biopsies, these genes are less differentially regulated between LS and NL or N controls. For example, while LCN2, WNT5A, and KRT6 (cluster 1) are all highly up-regulated in psoriasis vulgaris LS, these genes, which show constitutively high expression in scalp NL and N biopsies, are up-regulated to a lesser degree in LS scalp biopsies. In contrast, scalp tissue had lower expression of CD1A and CD207/langerin (clusters 2 and 3), which are markers of LCs. LCN2 gene encodes lipocalin-2, a protein involved in innate immunity that sequesters iron and consequently limits bacterial growth. Lipocalin-2 is expressed in neutrophils, monocytes, and keratinocytes; its serum levels are increased in patients with psoriasis [17] and correlates with disease severity [18]. KLK6 encodes a serine protease with activity against extracellular matrix proteins, such as fibronectin, laminin, vitronectin and collagen, and is elevated in both PsA synovial fluids and psoriatic plaques [19]. KRT6B encodes keratin 6B (Krt6), a type II cytokeratin that is expressed in the ors of hair follicles and glandular epithelia. WNT5A gene encodes Wnt5a, a protein member of the WNT family implicated in oncogenesis and in several developmental processes, including regulation of cell fate and patterning during embryogenesis. There is evidence for altered Wnt signaling in psoriatic skin [20], with higher Wnt5a expression in psoriatic plaques [21] While anti-TNF*α* treatment decreases WNT5A gene expression to less than 75%, WNT5A remains part of the molecular profile of resolved psoriasis [22]. The second cluster contains genes that are under-expressed in LS compared to both NL and N tissue. The most notable gene in this cluster is MMP28, which encodes epylisin, a novel human matrix metalloproteinase expressed in keratinocytes and associated with cell proliferation during repair after response to epithelial injury [23, 24]. The third cluster contains 10 genes that are suppressed in scalp and skin LS samples compared to NL scalp and skin and N skin samples. In this cluster, only three well known genes are included: CD1A, CD207/langerin and CYP2J2, encoding cytochrome P450, family 2, subfamily J, polypeptide 2 protein.

### GSEA and GSVA analyses of DEGs show activation of similar inflammatory and disease-related pathways in scalp and skin psoriasis

**Fig 3:**
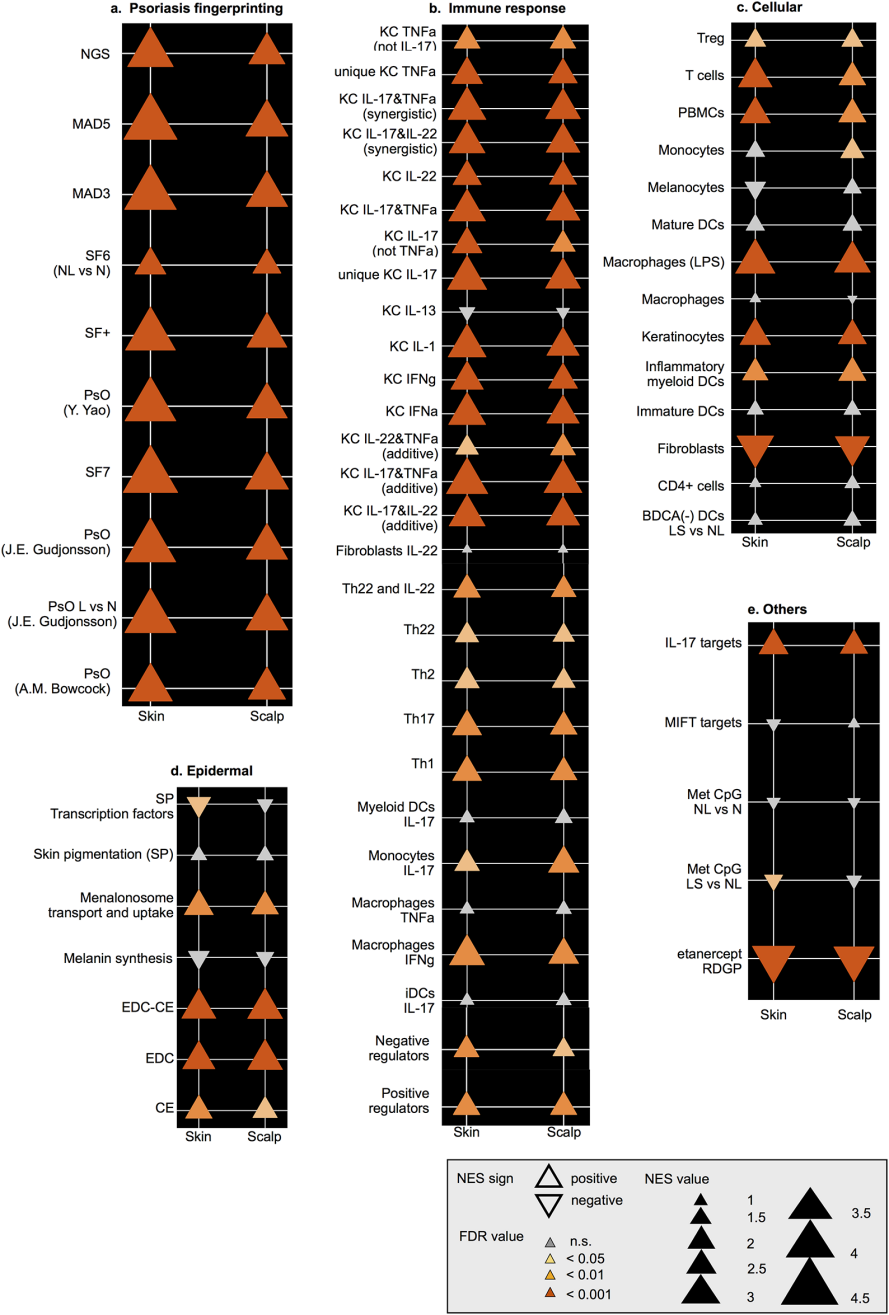
Triangle plot of GSEA results. Each panel represents NES values and p-values of GSEA analysis for LS and NL comparisons considering scalp vs skin areas for ‘psoriasis genetic print’ (a), ‘immune response’ (b, e), ‘cells biology’ (c), ‘epidermis biology’ (d), and ‘genetic regulation’ (f) gene-sets. NGS, next generation sequencing; MAD5, meta-analysis derived transcriptome of psoriasis 5; MAD3, meta-analysis derived transcriptome of psoriasis 3; SF7, Suarez-Farinas transcriptome 7; PsO, psoriasis; EDC, epidermal differentiation complex; CE, cornified envelope; KC, keratinocyte; TNF*α*, tumor necrosis factor alpha; IFNα, interferon α; IFNγ, interferon γ; PMBCs: peripheral blood mononucleated cells; DCs, dendritic cells; iDCs, immature dendritic cells; BDCA, blood dendritic cell antigen; Treg, regulatory T-cells; MIFT, microphthalmia-associated transcription factor; Met, methylated; RDGP, residual disease genomic profile.

To compare the scalp and skin transcriptomes, we used GSEA to determine correlations between the LS vs NL transcriptomes (ranked by FCHs) and previously published psoriasis pathways [25–29]. GSEA results showed significant enrichments of all gene-sets previously published for both skin and scalp transcriptomes with higher normalized enrichment scores (NES) for skin than scalp transcriptomes (Fig 3a–3e). For “core” pathways of cytokine-induced inflammatory responses (Fig 3b and 3e), as well as genes associated with specific leukocytes (Fig 3c), skin and scalp psoriasis showed very similar normalized enrichment scores. Thus, genes that are induced by TNF*α*, IL-17, IL-22, interferons, and other inflammatory cytokines are generally very similar in scalp and skin psoriasis. However, normalized GSEA enrichment scores of all disease transcriptome genes were slightly higher in skin LS than scalp LS, a finding which may be explained by constitutive expression of some psoriatic transcriptome genes in the follicular epithelium of NL scalp (as presented in Fig 3c and 3d). To estimate variations in pathway activity in the scalp psoriasis transcriptomes, we performed GSVA (Tables H-K in S1 File). Scalp transcriptomes were particularly enriched with ‘Basal vs upper epidermis’, ‘Melanocytes’, ‘Fibroblasts’ and ‘CD4^+^ T-cells’ and profiles of keratinocytes activated by INFγ or IL-13 and myeloid DCs induced by IL-17. Interestingly, enrichment of ‘negative regulators’ and ‘epigenetically regulated psoriasis-related genes’ was also observed. Skin transcriptomes were associated with significant enrichment of gene-sets related to ‘Epidermal Differentiation Complex’, ‘Cornified Envelope’, ‘Keratinocytes’, ‘T-cells’, ‘PBMCs’, ‘Macrophages’, ‘Mature DCs’, ‘Immature DCs’, ‘Th17’, ‘Th22’, and gene-sets related with TNF*α*/IL-17/IL-22-induced keratinocyte responses.

### Scalp psoriasis shows surface inflammation mediated by Th1/Th17 cytokines, which usually does not impede hair growth

**Fig 4:**
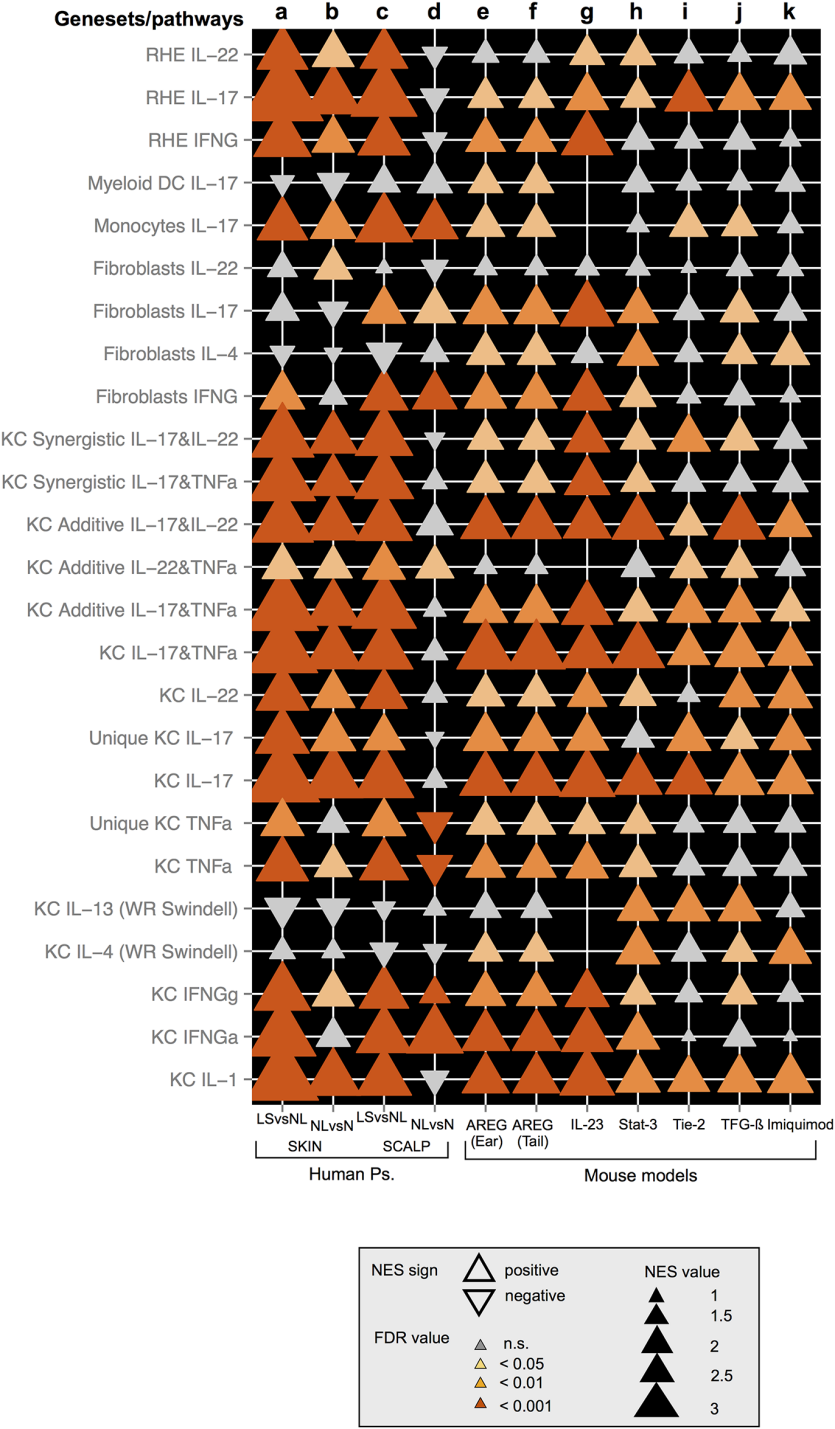
Enrichment of cytokine-related inflammatory pathways (gene sets) in human psoriasis transcriptomes as compared with murine models of psoriasis. Each panel represents NES values and p-values of GSEA analysis for LS and NL comparisons of STAT3, Tie2, and TGF*β* transgene models and the imiquimod-induced model; KC, keratinocyte; TNF*α*, tumor necrosis factor *α*; IFN*α*, interferon *α*; IFNγ, interferon γ; PMBCs: peripheral blood mononucleated cells; DCs, dendritic cells; iDCs, immature dendritic cells; BDCA, blood dendritic cell antigen; Treg, regulatory T cells; KC, keratinocytes; DCs, Myeloid dendritic cells; RHE, reconstituted human epidermis. (a-d) Expression of these cytokine pathways in human psoriasis vulgaris is shown for lesional (LS) versus non lesional (NL) and for NL versus normal (N) skin and scalp. (e–k) Cytokine enrichment in the six mouse models is illustrated as follows: K14-amphiregulin (AREG) ear skin (e) and tail skin (f), IL-23 (g), K5-Stat3C (Stat-3) (h), K5-Tie2 (Tie-2) (i), K5-TGF-*β*1 (TGF-*β*) (j), and Imiquimod (k).

When comparing immune gene-sets by GSEA, scalp psoriasis resembles skin psoriasis and to a much lesser extent the murine models (Fig 4). The STAT3, Tie2, and TGF*β* transgene models and the imiquimod-induced model represent murine models that express some inflammatory pathways that are present in psoriasis vulgaris, but with lower fidelity in the range of pathways that are expressed in human disease. In our case, IL-23-induced mouse transcriptome showed the greatest resemblance to human skin and scalp psoriasis. Even if the scalp is considered more similar to the skin of animal models, psoriasis remains a disease of the IFL skin even in the scalp.

**Fig 5:**
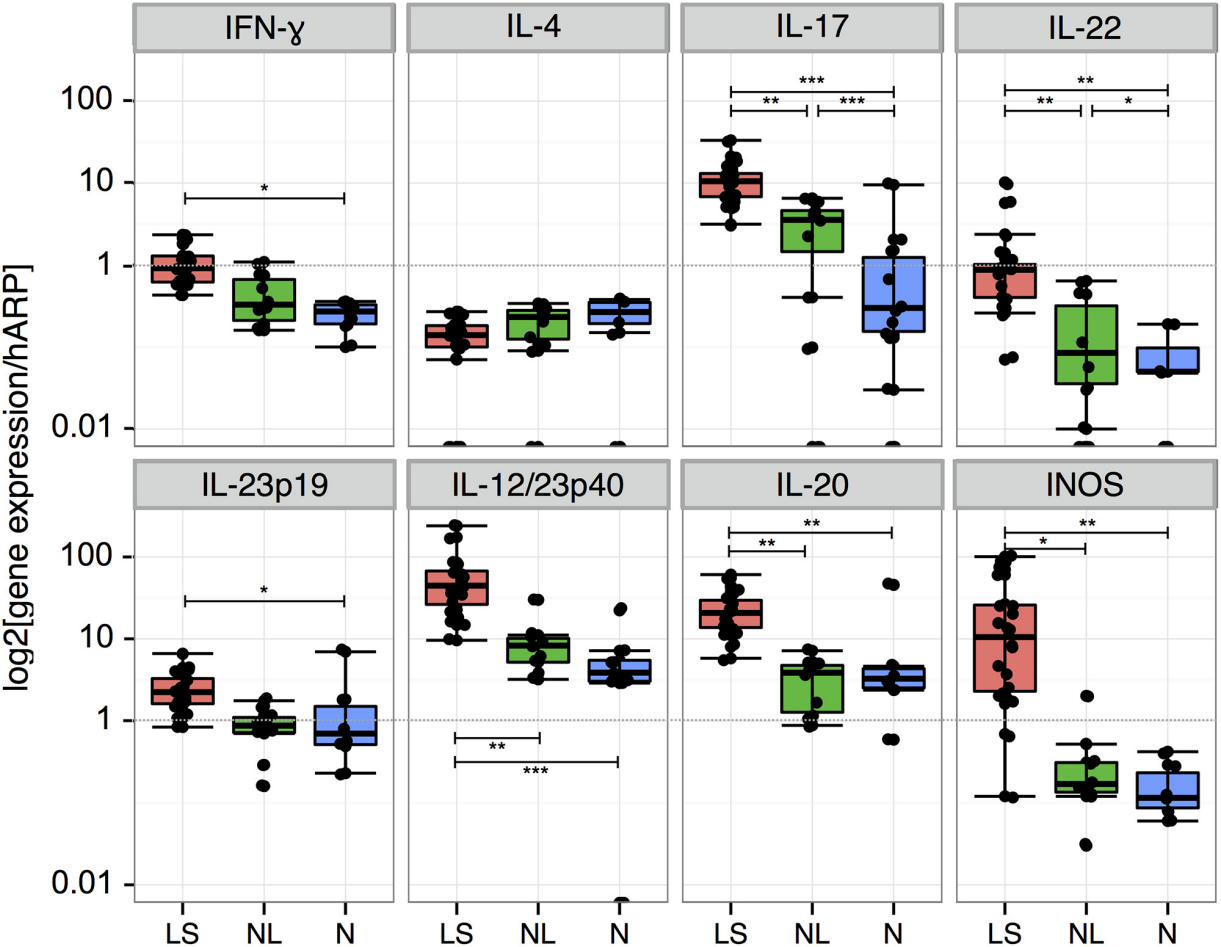
RT-PCR analysis of cytokine mRNA expression in scalp psoriasis. The cytokine microenvironment in the scalp biopsies of our study groups: Lesional (LS), non lesional (NL) and normal (N) scalp biopsies. Statistics: p<0.05 = *; p<0.01 = **; p<0.001 = ***.

To validate microarray findings, we performed RT-PCR. Expression of IFN-γ, IL-23p19, IL-12/23p40, IL-17 and IL-22 was higher in LS compared with NL scalp psoriasis samples, as previously described for LS psoriasis (Fig 5). We also analyzed IL-20 mRNA, a cytokine produced by inflammatory dendritic cells (iDCs) that affects keratinocyte activation and proliferation and found it to be significantly higher in LS than NL scalp samples (Fig 5). Higher mRNA expression of iNOS, an inducible nitric oxide synthase produced by Tip-DCs, was also observed in LS scalp, similar to previous reports about skin psoriasis.

**Fig 6:**
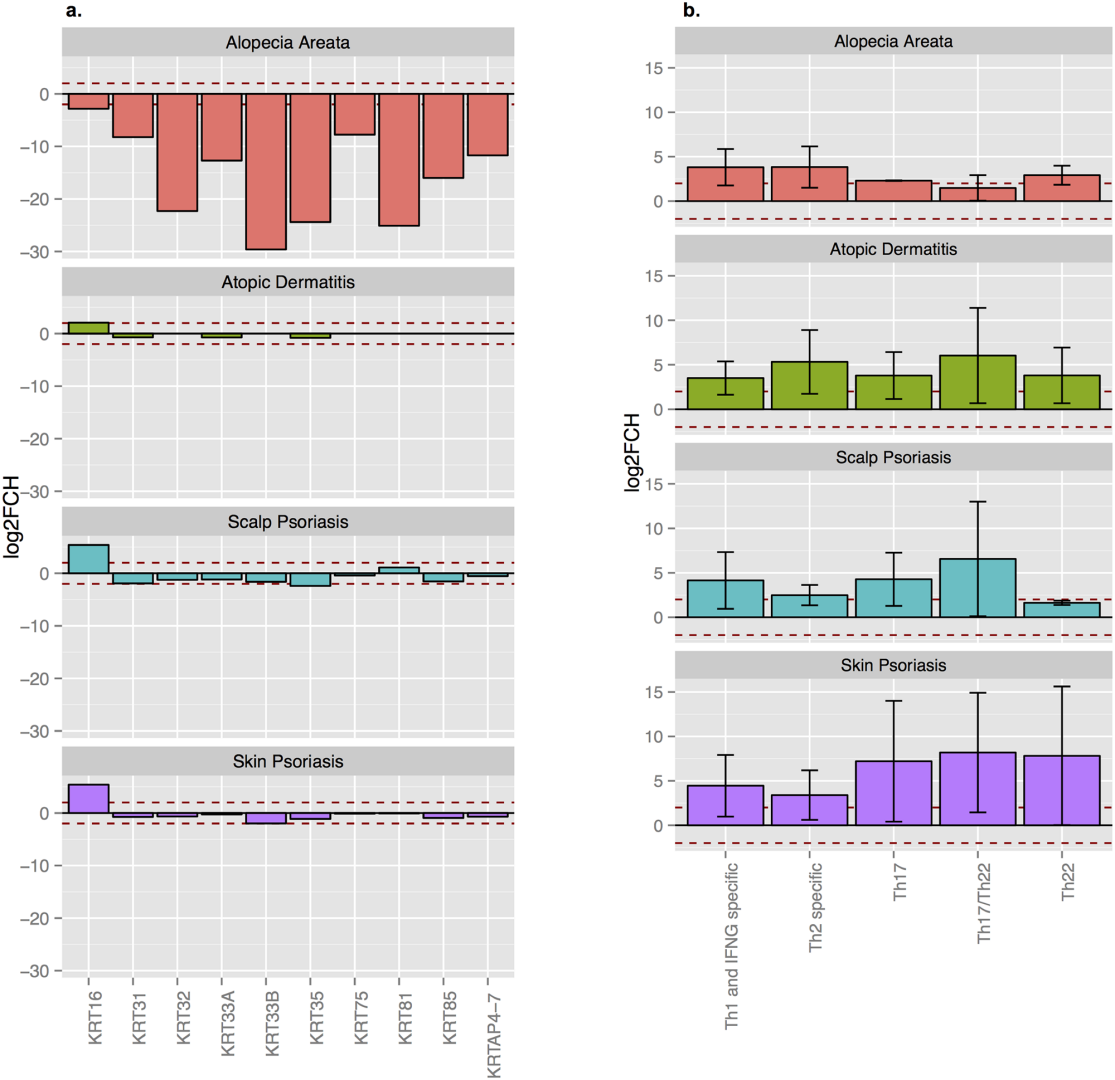
Keratin and immune-set gene expression profile of scalp psoriasis vs skin psoriasis, atopic dermatitis, and alopecia areata. Average log2FCH for alopecia areata (AA), atopic dermatitis (AA), scalp psoriasis and skin psoriasis for a representative group of (a) keratin and keratin associated proteins (KRTAPs) and (b) immune-related pathways genes adjusted by region (normalized to normal scalp [AA and scalp psoriasis] or normal skin [AD and skin psoriasis].

We also analyzed the immune-phenotype profiles of scalp and skin psoriasis compared with AA and AD. Scalp psoriasis showed a Th1/Th17 activation profile, similar to skin psoriasis, but with more Th1 and less Th22 activation. We also found significant differences compared to AA, which shows Th1/Th2 and IL-23 polarization and a lack of Th17 or Th22, and AD, which shows a higher expression of Th2/Th22 cytokines (Fig 6a).

Finally, increased KRT16 (K16) expression was found in psoriasis (scalp and skin), similar to AD, but without a significant decrease in other hair keratins (Fig 6b). On the contrary, in AA, a paradigm of inflammatory skin disease involving the hair follicle, all keratins were down-regulated, which reflects the alopecia seen in these patients.

## Discussion

This data represents the first report of a psoriasis scalp transcriptome showing that while overall there are similarities with the skin transcriptome there are also some important differences which might have therapeutic implications. Our results suggest two hypotheses: first, psoriasis remains a disease of the IFL skin even in the scalp; second, the immune mechanisms that mediate scalp psoriasis are consistent with psoriasis lesions involving other areas [30, 31]. However, there some differences in dysregulation, number of differentially expressed genes, and enrichment of the psoriatic genomic fingerprinting between scalp and skin lesions.

Psoriatic lesions are classically defined as scaly, erythematous patches with heavy infiltrate cell histology. The phenomenon of hyperkeratosis in psoriasis leads to loss of physiologic epidermal maturation, evidenced by our findings of increased K16 in the interfollicular epidermis and distal infundibular epithelium in the scalp. These findings are supported by prior studies showing K16 in lesional and nonlesional skin psoriasis [32]. To best classify the psoriasis disease process in the scalp, one needs to consider the differing biology of two epithelial zones: the superficial epithelium, composed of the interfollicular epidemis and the upper regions of hair follicles that insert into the epidermis (acrotrichial epithelium), where keratin 6/16 are not constitutively expressed, and the lower portions of the hair follicle, where the keratinocytes do constitutively express keratins 6/16. The process of psoriasis in the scalp induces expression of keratin 16 in the interfollicular epidermis and acrotrichial epithelium, but it does not further induce keratin 16 expression in follicular keratinocytes that constitutively express this keratin. In scalp psoriasis, surface inflammation usually does not impede hair growth and thus alopecia does not occur. In our study, we observed normal architecture of the lower portions of the hair follicle without a significant immune infiltrate. At the genomic level, we detected only a very small decrease in expression of keratin genes associated with hair fiber formation (meanÃƒâ€š 2-fold decrease) in NL and LS scalp, whereas most of these genes were reduced by >100-fold in scalp biopsies of AA. In contrast, while small reductions in hair keratins are seen in scalp psoriasis, a marked increase in K16 is seen by the same analysis, due to up-regulation in the intefollicular epidermis.

The epidermis functions not only as a physical barrier, but also as a chemical and immunologic barrier. In skin from patients with psoriasis, alterations in keratinocyte function produce inflammatory factors that promote chronic, self-amplifying loops of immune activity [33, 34]. The composition of cellular infiltrate of DCs and T-cell subpopulations is identical in both scalp and skin psoriasis [35]. Regarding infiltrating leukocytes, psoriasis contains large populations of both T lymphocytes and DCs, with slightly higher T-cells [30]. In our study, the IFL areas of scalp psoriasis were infiltrated with CD3^+^ and CD8^+^ cells. In psoriasis, T-cells (both CD4^+^ and CD8^+^) are known to cause low production of Th2 cytokines (IFNγ, TNF*α*, and IL-2) and high production of Th1 cytokines (IL-4, IL-6 and IL-10), leading to polarization of the type 1 pathway [34]. In scalp psoriasis, the Th1 profile was confirmed by the up-regulation of IFNγ in the RT-PCR analysis. Taken together, the histological characterization and distribution of the T-cells in lesional scalp biopsies and its cytokine environment showed similarities between IFL scalp psoriasis and psoriasis on other body parts [36].

The scalp transcriptome showed increased modulation of several gene-sets, particularly those induced by keratinocytes IFNγ compared with skin psoriasis samples, which were mainly associated with significantly higher activation of genes involved in the TNF*α*/IL-17/IL-22-induced keratinocyte responses. Scalp psoriasis transcriptome showed a profile of Th1- and Th17-cytokines activation, similar to skin psoriasis. This fact is important since it indicates that patients with psoriasis vulgaris with involvement of the scalp could benefit from a new generation of monoclonal antibodies targeting the Th17/IL-17 pathway such as ixekizumab, secukinumab, brodalumab or tildrakizumab [37].

GSEA and GSVA analyses revealed a specific role for CD4^+^ and regulatory T-cells (Tregs) in scalp but not skin psoriasis. Treg cells are crucial for immune homeostasis and prevention of pathological skewing of immune responses in tissues. In patients with psoriasis, Tregs were reported to be both quantitatively and functionally deficient in the ability to suppress T-cell activation [38]. In our study, an enrichment of the Treg genetic signature was found in scalp compared with skin psoriasis. Interestingly, sotrastaurin, a pan-protein kinase C (PKC) inhibitor, has been shown to decrease effector T-cell responses and increase regulatory responses by enhancing Foxp3 expression and preventing IL-17A and IFNγ production, even in the presence of Th17 inducing cytokines such as IL-1*β* or IL-23 [39]. Maintaining Treg stability with this new drug could be a potential therapeutic target in the context of inflammation associated with scalp psoriasis. Thus, new therapeutic approaches could focus on specific upregulated genes or pathways in scalp psoriasis. Another example is a treatment targeting cannabinoids receptors, which have been described control elements of human Krt6 expression, in order to decrease proliferation of epidermal keratinocytes [40].

Psoriasis is only observed in humans, but numerous genetic approaches to model the disease in mice have been undertaken [7, 41]. Histopathological and immune features observed in human psoriasis are also observed in murine models of psoriasis-like disease, although distinct differences are apparent. The main difference is that the mouse skin is heavily populated by hair follicles, whereas the human epidermis is mainly IFL. Furthermore, mouse skin is characterized by a lack of sweat glands and melanocytes in the IFL epidermis, a synchronized hair cycle, rapid epidermal turnover and the presence of particular subtypes of immune cells such as intraepidermal γ δT cells, CD8^+^ DCs, dendritic epidermal T cells and natural killer (NK)1.1^+^ T cells, which are absent in human skin [7]. In our study, most of the immune cell infiltrates that differ between LS and NL samples were observed in the IF area of the scalp. In the inFD area, only the peri-infundibular epithelium showed a thicker epidermis, a positive K16 suprabasal staining and a significantly higher CD3^+^ cells infiltrate in LS than NL biopsies. Thus, murine models should not be considered the gold standard to study scalp psoriasis. Based on our results, their use could hypothetically show similar limitations to those previously found in the study of skin psoriasis.

Overall, features of psoriasis in interfollicular scalp lesions and skin lesions are very similar. The psoriatic phenotype may extend into the follicular infundibulum, but it does not apparently alter lower portions of the hair follicle or hair growth. The involvement of the scalp in psoriasis is very frequent, and sometimes can be the only manifestation of disease and poses a difficult therapeutic problem. The aim of this study was to describe the immunopathology and genomic fingerprinting of scalp psoriasis and to compare the results with earlier findings of psoriasis on other areas of the body. Furthermore, our data reveal similarities and differences of immune regulation between scalp and skin psoriasis with potential therapeutic implications. Recently Jiang et al. reviewed the results of genomics-based technologies utilized for biomarker discovery in psoriasis [42]. Biomarkers identified by transcriptome analysis may provide additional insights into the molecular mechanisms and signaling pathways involved in scalp psoriasis pathogenesis and could serve as both targets for novel therapeutic interventions and surrogate/predictive markers for treatment outcome in psoriasis of this skin area. Gaining a better understanding of the pathogenesis of scalp psoriasis will lead to more efficient novel treatment strategies for scalp psoriasis. Future clinical studies are needed to assess novel therapeutic agents targeting scalp-specific genes or pathways.

## Supporting Information

S1 File Study design and analysis workflow (Figure A), the distribution of keratin 16 (K16) staining and epidermal/epithelial thickness in the various histological compartments of the scalp biopsies (Figure B(a-c)), anti bodies used for immunohistochemistry (Table A), upregulated genes in scalp psoriasis: lesional vs. non-lesional (Table B), downregulated genes in scalp psoriasis: lesional vs. non-lesional (Table C), upregulated genes in scalp psoriasis: lesional vs. normal (Table D), downregulated genes in scalp psoriasis: non-lesional vs. normal (Table E), upregulated genes in scalp psoriasis: non-lesional vs. normal (Table F), downregulated genes in scalp skin: non-lesional vs. normal (Table G), Gene Set Variation Analysis (GSVA): ‘Epidermal Biology’ and ‘Cells’ groups of gene sets (Table H), Gene Set Variation Analysis (GSVA): ‘Immune response’ group of gene sets (Table I), Gene Set Variation Analysis (GSVA): ‘Psoriasis’ group of gene sets (Table J) and Gene Set Variation Analysis (GSVA): ‘Genetic regulation’ group of gene sets (Table K).
(PDF)
