# Supplementary material for: Molecular and Cellular Profiling of Scalp Psoriasis Reveals Differences and Similarities Compared to Skin Psoriasis
Source: PLoS One. 2016 Feb 5;11(2):e0148450. doi: 10.1371/journal.pone.0148450 (PMC4743842; doi:10.1371/journal.pone.0148450)
Supplement: S1 File — Study design and analysis workflow (Figure A), the distribution of keratin 16 (K16) staining and epidermal/epithelial thickness in the various histological compartments of the scalp biopsies (Figure B(a-c)), anti bodies used for immunohistochemistry (Table A), upregulated genes in scalp psoriasis: lesional vs. non-lesional (Table B), downregulated genes in scalp psoriasis: lesional vs. non-lesional (Table C), upregulated genes in scalp psoriasis: lesional vs. normal (Table D), downregulated genes in scalp psoriasis: non-lesional vs. normal (Table E), upregulated genes in scalp psoriasis: non-lesional vs. normal (Table F), downregulated genes in scalp skin: non-lesional vs. normal (Table G), Gene Set Variation Analysis (GSVA): ‘Epidermal Biology’ and ‘Cells’ groups of gene sets (Table H), Gene Set Variation Analysis (GSVA): ‘Immune response’ group of gene sets (Table I), Gene Set Variation Analysis (GSVA): ‘Psoriasis’ group of gene sets (Table J) and Gene Set Variation Analysis (GSVA): ‘Genetic regulation’ group of gene sets (Table K). (PDF) [file pone.0148450.s001.pdf]

Figure A

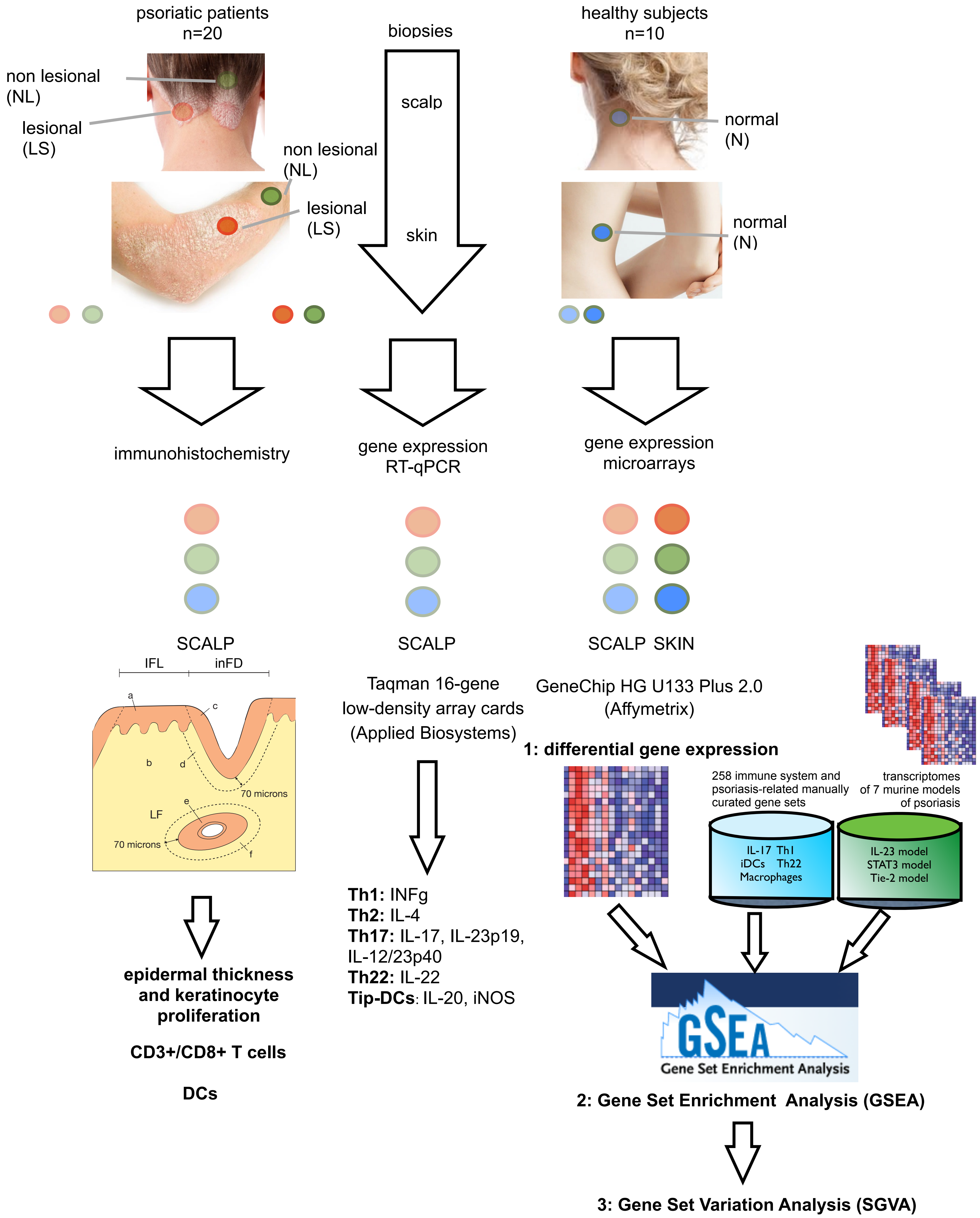

Figure A. Study design and analysis workflow. In scalp biopsies different areas of interest were considered: IFL (interfollicular area [epidermis and dermis]), inFD [infundibulum (epithelium and peri-infundibular area)] and LF [lower follicle (outer root sheath and perifollicular area)]. a) interfollicular epidermis; b) interfollicular dermis; c) infundibular epithelium (upper part and lower part-for definition see text); d) periinfundibular area (width: 70 microns); e) outer root sheath (ors.); f) perifollicular area (width: 70 microns).

Figure B

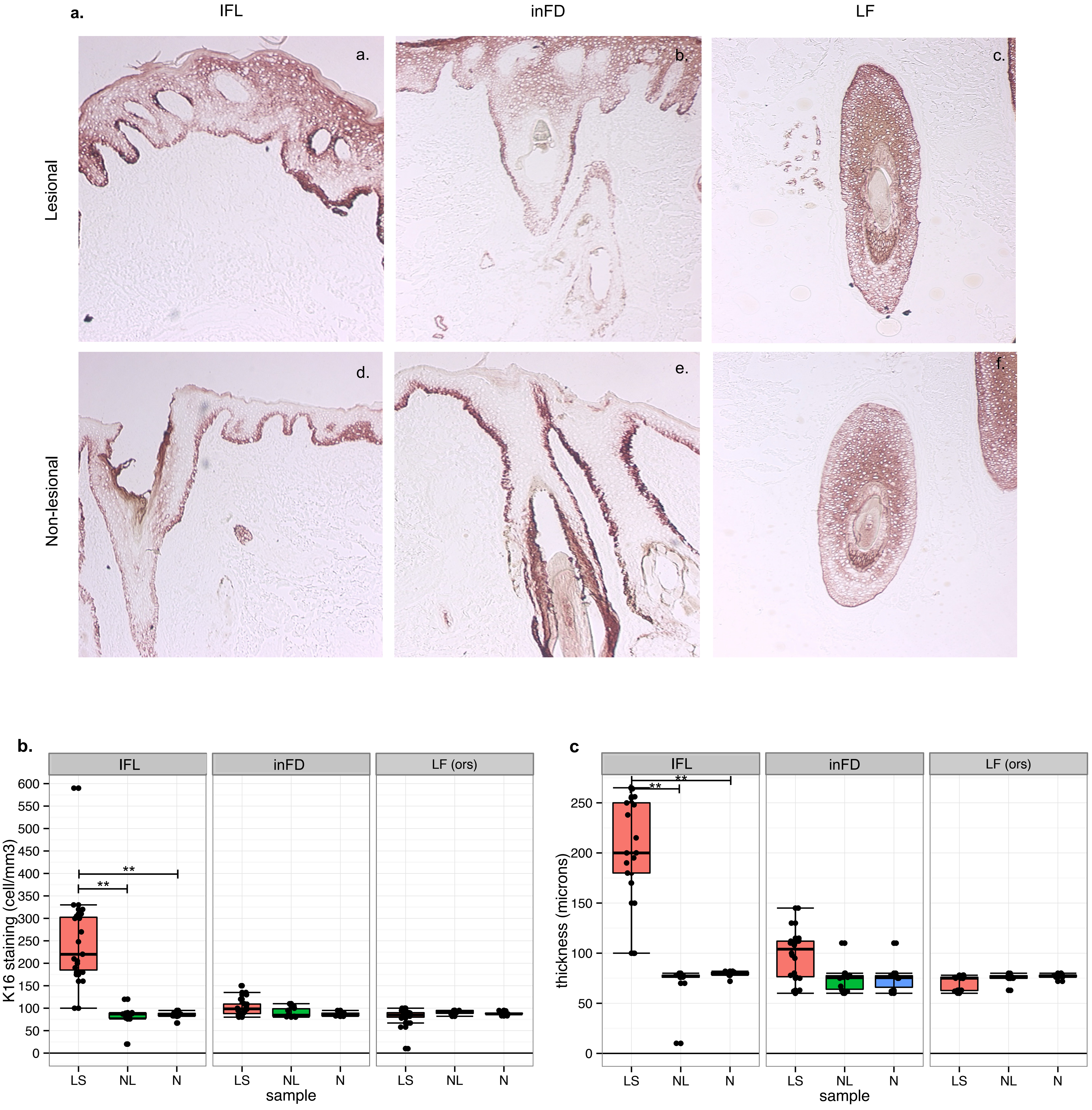

Figure B (a-c). The distribution of keratin 16 (K16) staining and epidermal/epithelial thickness in the various histological compartments of the scalp biopsies. Panels a and b show the staining distribution and the count analysis of keratin 16 (K16). Panel c represents epidermal/epithelial thickness of the various subject groups and compartments. Patient groups are: LS (lesional biopsies); NL (nonlesional). Panels a and d show representative pictures of the IFL area of the patients; panels b and e show the inFD areas; panels c and f represent the LF. Panels a and b show the biopsies from lesional scalp (LS); panels d and e show the biopsies from nonlesional (NL) scalp. Results of statistical comparisons: p< 0,05 = *; 0,01<p<0,05 = **; p<0,01 = ***. Differences not indicated are statistically nonsignificant.

Table A. Anti bodies used for immunohistochemistry.

**Marker / dilution**	**Manufacturer**	**Clone**
CD3 1:100	BD-Pharmingen, San Diego, Calif., USA	SK7
CD11c 1:100	BD-Pharmingen, San Diego, Calif., USA	B-ly6
CD83 1:100	BD-Pharmingen, San Diego, Calif., USA	HB15e
DC-Lamp 1:100	ImmunoTech, Westbrook, Me, USA	104 G4
CD207 (Langerin) 1:100	ImmunoTech, Westbrook, Me, USA	DCG m4
CD8 1:100	BD-Pharmingen, San Diego, Calif., USA	HIT8a
BDCA-1+ 10ug/ml	Miltenyi Biotec GmbH, Bergish Gladbach, Germany	AD5-8E7
BDCA-2 10 ug/ml	Miltenyi Biotec GmbH, Bergish Gladbach, Germany	AC-144
CD68 1:100	BD-Pharmingen, San Diego, Calif., USA	Yi/182A
DC205 10ug/ml	Gift from prof. R. Steinman, Rockefeller University, New York, USA	-
K16 1:1000	ImmunoTech, Westbrook, Me, USA	K8.12

Table B. Upregulated genes in scalp psoriasis: lesional vs. non-lesional.

	Probe	Symbol	Description	FCH-Scalp-LS-NL	log2FCH-Scalp-LS-NL	p-Scalp-LS-NL	FDR-Scalp-LS-NL
1	205513_at	TCN1	transcobalamin I (vitamin B12 binding protein, R binder family)	174.921	7.451	0.000	0.000
2	205863_at	S100A12	S100 calcium binding protein A12	89.893	6.490	0.000	0.000
3	207356_at	DEFB4	defensin, beta 4	47.980	5.584	0.000	0.000
4	211906_s_at	SERPINB4	serpin peptidase inhibitor, clade B (ovalbumin), member 4	37.372	5.224	0.000	0.000
5	220664_at	SPRR2C	small proline-rich protein 2C	31.775	4.990	0.000	0.000
6	210663_s_at	KYNU	kynureninase (L-kynurenine hydrolase)	31.203	4.964	0.000	0.000
7	220322_at	IL1F9	interleukin 1 family, member 9	27.280	4.770	0.000	0.000
8	217388_s_at	KYNU	kynureninase (L-kynurenine hydrolase)	23.750	4.570	0.000	0.000
9	205783_at	KLK13	kallikrein-related peptidase 13	23.733	4.569	0.000	0.000
10	206561_s_at	AKR1B10	aldo-keto reductase family 1, member B10 (aldose reductase)	23.667	4.565	0.000	0.000
11	202859_x_at	IL8	interleukin 8	23.210	4.537	0.001	0.000
12	203691_at	PI3	peptidase inhibitor 3, skin-derived (SKALP)	20.876	4.384	0.000	0.000
13	205660_at	OASL	2'-5'-oligoadenylate synthetase-like	19.213	4.264	0.000	0.000
14	217315_s_at	KLK13	kallikrein-related peptidase 13	16.328	4.029	0.000	0.000
15	204385_at	KYNU	kynureninase (L-kynurenine hydrolase)	15.962	3.997	0.000	0.000
16	207602_at	TMPRSS11D	transmembrane protease, serine 11D	15.718	3.974	0.000	0.000
17	219403_s_at	HPSE	heparanase	15.483	3.953	0.000	0.000
18	41469_at	PI3	peptidase inhibitor 3, skin-derived (SKALP)	15.433	3.948	0.000	0.000
19	215891_s_at	GM2A	GM2 ganglioside activator	14.908	3.898	0.000	0.000
20	204972_at	OAS2	2'-5'-oligoadenylate synthetase 2, 69/71kDa	14.624	3.870	0.000	0.000
21	210413_x_at	SERPINB4	serpin peptidase inhibitor, clade B (ovalbumin), member 4	14.320	3.840	0.000	0.000
22	33646_g_at	GM2A	GM2 ganglioside activator	14.220	3.830	0.000	0.000
23	207367_at	ATP12A	ATPase, H+/K+ transporting, nongastric, alpha polypeptide	13.435	3.748	0.000	0.000
24	210797_s_at	OASL	2'-5'-oligoadenylate synthetase-like	11.506	3.524	0.000	0.000
25	211788_s_at	TREX2	three prime repair exonuclease 2	11.186	3.484	0.000	0.000
26	219554_at	RHCG	Rh family, C glycoprotein	10.916	3.448	0.000	0.000
27	216258_s_at	SERPINB13	serpin peptidase inhibitor, clade B (ovalbumin), member 13	10.862	3.441	0.000	0.000
28	204733_at	KLK6	kallikrein-related peptidase 6	10.754	3.427	0.000	0.000
29	209720_s_at	SERPINB3	serpin peptidase inhibitor, clade B (ovalbumin), member 3	10.556	3.400	0.000	0.000
30	211362_s_at	SERPINB13	serpin peptidase inhibitor, clade B (ovalbumin), member 13	10.496	3.392	0.000	0.000
31	211506_s_at	IL8	interleukin 8	10.034	3.327	0.003	0.020
32	209719_x_at	SERPINB3	serpin peptidase inhibitor, clade B (ovalbumin), member 3	9.988	3.320	0.000	0.000
33	205476_at	CCL20	chemokine (C-C motif) ligand 20	9.788	3.291	0.000	0.000
34	221107_at	CHRNA9	cholinergic receptor, nicotinic, alpha 9	9.482	3.245	0.000	0.000
35	220658_s_at	ARNTL2	aryl hydrocarbon receptor nuclear translocator-like 2	8.643	3.111	0.000	0.000
36	203535_at	S100A9	S100 calcium binding protein A9	8.377	3.066	0.000	0.000
37	220187_at	STEAP4	STEAP family member 4	8.026	3.005	0.000	0.000
38	209727_at	GM2A	GM2 ganglioside activator	8.015	3.003	0.000	0.000
39	204470_at	CXCL1	chemokine (C-X-C motif) ligand 1 (melanoma growth stimulating activity, alpha)	7.954	2.992	0.000	0.000
40	209773_s_at	RRM2	ribonucleotide reductase M2 polypeptide	7.828	2.969	0.000	0.000
41	202018_s_at	LTF	lactotransferrin	7.745	2.953	0.000	0.000
42	203699_s_at	DIO2	deiodinase, iodothyronine, type II	7.309	2.870	0.000	0.000
43	210652_s_at	C1orf34	chromosome 1 open reading frame 34	7.086	2.825	0.000	0.000
44	216202_s_at	SPTLC2	serine palmitoyltransferase, long chain base subunit 2	7.041	2.816	0.000	0.000
45	204415_at	IFI6	interferon, alpha-inducible protein 6	7.025	2.812	0.000	0.000
46	208539_x_at	SPRR2D	small proline-rich protein 2D	6.976	2.802	0.000	0.000
47	203127_s_at	SPTLC2	serine palmitoyltransferase, long chain base subunit 2	6.964	2.800	0.000	0.000
48	203779_s_at	MPZL2	myelin protein zero-like 2	6.960	2.799	0.000	0.000
49	203234_at	UPP1	uridine phosphorylase 1	6.870	2.780	0.000	0.000
50	219352_at	HERC6	hect domain and RLD 6	6.634	2.730	0.000	0.000
51	215223_s_at	SOD2	superoxide dismutase 2, mitochondrial	6.547	2.711	0.000	0.000
52	218810_at	ZC3H12A	zinc finger CCCH-type containing 12A	6.504	2.701	0.000	0.000
53	213562_s_at	SQLE	squalene epoxidase	6.431	2.685	0.000	0.000
54	218400_at	OAS3	2'-5'-oligoadenylate synthetase 3, 100kDa	6.386	2.675	0.000	0.000
55	210138_at	RGS20	regulator of G-protein signaling 20	6.379	2.673	0.000	0.000
56	219850_s_at	EHF	ets homologous factor	6.210	2.635	0.000	0.000
57	32128_at	CCL18	chemokine (C-C motif) ligand 18 (pulmonary and activation-regulated)	6.204	2.633	0.002	0.010
58	212531_at	LCN2	lipocalin 2 (oncogene 24p3)	6.101	2.609	0.000	0.000
59	204858_s_at	ECGF1	endothelial cell growth factor 1 (platelet-derived)	6.078	2.604	0.000	0.000
60	214490_at	ARSF	arylsulfatase F	5.987	2.582	0.000	0.000
61	205242_at	CXCL13	chemokine (C-X-C motif) ligand 13 (B-cell chemoattractant)	5.931	2.568	0.004	0.020
62	214581_x_at	TNFRSF21	tumor necrosis factor receptor superfamily, member 21	5.786	2.533	0.000	0.000
63	214007_s_at	TWF1	twinfilin, actin-binding protein, homolog 1 (Drosophila)	5.785	2.532	0.000	0.000
64	213060_s_at	CHI3L2	chitinase 3-like 2	5.745	2.522	0.000	0.000
65	202086_at	MX1	myxovirus (influenza virus) resistance 1, interferon-inducible protein p78 (mouse)	5.610	2.488	0.000	0.000
66	206628_at	SLC5A1	solute carrier family 5 (sodium/glucose cotransporter), member 1	5.602	2.486	0.000	0.000
67	218960_at	TMPRSS4	transmembrane protease, serine 4	5.555	2.474	0.000	0.000
68	205552_s_at	OAS1	2',5'-oligoadenylate synthetase 1, 40/46kDa	5.546	2.471	0.000	0.000
69	203915_at	CXCL9	chemokine (C-X-C motif) ligand 9	5.517	2.464	0.003	0.020
70	212268_at	SERPINB1	serpin peptidase inhibitor, clade B (ovalbumin), member 1	5.516	2.464	0.000	0.000
71	205844_at	VNN1	vanin 1	5.413	2.437	0.001	0.000
72	200796_s_at	MCL1	myeloid cell leukemia sequence 1 (BCL2-related)	5.368	2.424	0.000	0.000
73	222223_s_at	IL1F5	interleukin 1 family, member 5 (delta)	5.335	2.416	0.000	0.000
74	220780_at	PLA2G3	phospholipase A2, group III	5.260	2.395	0.000	0.000
75	220528_at	VNN3	vanin 3	5.247	2.391	0.000	0.000
76	211919_s_at	CXCR4	chemokine (C-X-C motif) receptor 4	5.233	2.388	0.000	0.000
77	215977_x_at	GK	glycerol kinase	5.190	2.376	0.000	0.000
78	209772_s_at	CD24	CD24 molecule	5.182	2.373	0.000	0.000

	Probe	Symbol	Description	FCH-Scalp-LS-NL	log2FCH-Scalp-LS-NL	p-Scalp-LS-NL	FDR-Scalp-LS-NL
79	202411_at	IFI27	interferon, alpha-inducible protein 27	5.178	2.372	0.000	0.000
80	206008_at	TGM1	transglutaminase 1 (K polypeptide epidermal type I, protein-glutamine-gamma-glutamyltransferase)	5.094	2.349	0.000	0.000
81	206133_at	XAF1	XIAP associated factor-1	5.086	2.347	0.000	0.000
82	214453_s_at	IFI44	interferon-induced protein 44	5.028	2.330	0.000	0.000
83	202869_at	OAS1	2',5'-oligoadenylate synthetase 1, 40/46kDa	5.006	2.324	0.000	0.000
84	215150_at	YOD1	YOD1 OTU deubiquinating enzyme 1 homolog (S. cerevisiae)	4.927	2.301	0.001	0.000
85	204580_at	MMP12	matrix metallopeptidase 12 (macrophage elastase)	4.916	2.297	0.003	0.010
86	211561_x_at	MAPK14	mitogen-activated protein kinase 14	4.846	2.277	0.000	0.000
87	209924_at	CCL18	chemokine (C-C motif) ligand 18 (pulmonary and activation-regulated)	4.805	2.265	0.004	0.020
88	207387_s_at	GK	glycerol kinase	4.756	2.250	0.000	0.000
89	209514_s_at	RAB27A	RAB27A, member RAS oncogene family	4.756	2.250	0.000	0.000
90	215808_at	KLK10	kallikrein-related peptidase 10	4.749	2.248	0.001	0.000
91	218384_at	CARHSP1	calcium regulated heat stable protein 1, 24kDa	4.596	2.201	0.000	0.000
92	204533_at	CXCL10	chemokine (C-X-C motif) ligand 10	4.564	2.190	0.001	0.010
93	205483_s_at	ISG15	ISG15 ubiquitin-like modifier	4.436	2.149	0.000	0.000
94	205729_at	OSMR	oncostatin M receptor	4.429	2.147	0.000	0.000
95	208621_s_at	VIL2	villin 2 (ezrin)	4.386	2.133	0.002	0.010
96	203214_x_at	CDC2	cell division cycle 2, G1 to S and G2 to M	4.350	2.121	0.000	0.000
97	210608_s_at	FUT2	fucosyltransferase 2 (secretor status included)	4.298	2.104	0.000	0.000
98	204995_at	CDK5R1	cyclin-dependent kinase 5, regulatory subunit 1 (p35)	4.295	2.103	0.000	0.000
99	210087_s_at	MPZL1	myelin protein zero-like 1	4.290	2.101	0.000	0.000
100	211834_s_at	TP63	tumor protein p63	4.288	2.100	0.002	0.010
101	203180_at	ALDH1A3	aldehyde dehydrogenase 1 family, member A3	4.276	2.096	0.001	0.000
102	219225_at	PGBD5	piggyBac transposable element derived 5	4.276	2.096	0.000	0.000
103	210904_s_at	IL13RA1	interleukin 13 receptor, alpha 1	4.265	2.093	0.000	0.000
104	207381_at	ALOX12B	arachidonate 12-lipoxygenase, 12R type	4.257	2.090	0.000	0.000
105	204941_s_at	ALDH3B2	aldehyde dehydrogenase 3 family, member B2	4.230	2.081	0.006	0.030
106	216841_s_at	SOD2	superoxide dismutase 2, mitochondrial	4.185	2.065	0.000	0.000
107	217167_x_at	GK	glycerol kinase	4.161	2.057	0.000	0.000
108	200841_s_at	EPRS	glutamyl-prolyl-tRNA synthetase	4.152	2.054	0.000	0.000
109	221765_at	UGCG	UDP-glucose ceramide glucosyltransferase	4.126	2.045	0.000	0.000
110	205014_at	FGFBP1	fibroblast growth factor binding protein 1	4.105	2.037	0.000	0.000
111	207463_x_at	PRSS3	protease, serine, 3 (mesotrypsin)	4.092	2.033	0.000	0.000
112	221698_s_at	CLEC7A	C-type lectin domain family 7, member A	4.078	2.028	0.000	0.000
113	220249_at	HYAL4	hyaluronoglucosaminidase 4	4.074	2.027	0.000	0.000
114	201490_s_at	PPIF	peptidylprolyl isomerase F (cyclophilin F)	4.055	2.020	0.000	0.000
115	202134_s_at	WWTR1	WW domain containing transcription regulator 1	4.034	2.012	0.000	0.000
116	220330_s_at	SAMSN1	SAM domain, SH3 domain and nuclear localization signals 1	4.030	2.011	0.001	0.010
117	219722_s_at	GDPD3	glycerophosphodiester phosphodiesterase domain containing 3	4.021	2.008	0.000	0.000
118	206667_s_at	SCAMP1	secretory carrier membrane protein 1	4.017	2.006	0.003	0.010
119	202831_at	GPX2	glutathione peroxidase 2 (gastrointestinal)	4.014	2.005	0.000	0.000
120	205681_at	BCL2A1	BCL2-related protein A1	4.011	2.004	0.007	0.030
121	210951_x_at	RAB27A	RAB27A, member RAS oncogene family	4.006	2.002	0.000	0.000
122	216470_x_at	TRY6	trypsinogen C	3.944	1.980	0.000	0.000
123	210449_x_at	MAPK14	mitogen-activated protein kinase 14	3.941	1.978	0.000	0.000
124	216244_at	IL1RN	interleukin 1 receptor antagonist	3.932	1.975	0.000	0.000
125	201130_s_at	CDH1	cadherin 1, type 1, E-cadherin (epithelial)	3.902	1.964	0.007	0.030
126	201169_s_at	BHLHB2	basic helix-loop-helix domain containing, class B, 2	3.902	1.964	0.000	0.000
127	217497_at	ECGF1	endothelial cell growth factor 1 (platelet-derived)	3.896	1.962	0.000	0.000
128	209774_x_at	CXCL2	chemokine (C-X-C motif) ligand 2	3.882	1.957	0.002	0.010
129	202626_s_at	LYN	v-yes-1 Yamaguchi sarcoma viral related oncogene homolog	3.861	1.949	0.000	0.000
130	214710_s_at	CCNB1	cyclin B1	3.829	1.937	0.000	0.000
131	219630_at	PDZK1IP1	PDZK1 interacting protein 1	3.822	1.934	0.000	0.000
132	206177_s_at	ARG1	arginase, liver	3.814	1.931	0.000	0.000
133	209546_s_at	APOL1	apolipoprotein L, 1	3.805	1.928	0.000	0.000
134	209800_at	KRT16	keratin 16 (focal non-epidermolytic palmoplantar keratoderma)	3.802	1.927	0.001	0.000
135	205829_at	HSD17B1	hydroxysteroid (17-beta) dehydrogenase 1	3.777	1.917	0.000	0.000
136	209201_x_at	CXCR4	chemokine (C-X-C motif) receptor 4	3.747	1.906	0.000	0.000
137	205724_at	PKP1	plakophilin 1 (ectodermal dysplasia/skin fragility syndrome)	3.723	1.897	0.002	0.010
139	213421_x_at	PRSS3	protease, serine, 3 (mesotrypsin)	3.718	1.894	0.000	0.000
140	219795_at	SLC6A14	solute carrier family 6 (amino acid transporter), member 14	3.713	1.892	0.001	0.010
141	214059_at	IFI44	interferon-induced protein 44	3.700	1.888	0.001	0.000
143	210164_at	GZMB	granzyme B (granzyme 2, cytotoxic T-lymphocyte-associated serine esterase 1)	3.695	1.886	0.001	0.010
145	208650_s_at	CD24	CD24 molecule	3.660	1.872	0.000	0.000
146	218856_at	TNFRSF21	tumor necrosis factor receptor superfamily, member 21	3.660	1.872	0.000	0.000
147	219956_at	GALNT6	UDP-N-acetyl-alpha-D-galactosamine:polypeptide N-acetylgalactosaminyltransferase 6 (GalNAc-T6)	3.638	1.863	0.000	0.000
148	218990_s_at	SPRR3	small proline-rich protein 3	3.603	1.849	0.005	0.020
149	205774_at	F12	coagulation factor XII (Hageman factor)	3.584	1.841	0.000	0.000
150	215966_x_at	GK3P	glycerol kinase 3 pseudogene	3.579	1.840	0.000	0.000
151	209969_s_at	STAT1	signal transducer and activator of transcription 1, 91kDa	3.563	1.833	0.000	0.000
152	209872_s_at	PKP3	plakophilin 3	3.555	1.830	0.001	0.000
153	205554_s_at	DNASE1L3	deoxyribonuclease I-like 3	3.545	1.826	0.001	0.000
154	212021_s_at	MKI67	antigen identified by monoclonal antibody Ki-67	3.545	1.826	0.000	0.000
155	204439_at	IFI44L	interferon-induced protein 44-like	3.497	1.806	0.012	0.040
156	202357_s_at	CFB	complement factor B	3.488	1.802	0.000	0.000
157	206211_at	SELE	selectin E (endothelial adhesion molecule 1)	3.479	1.799	0.002	0.010
158	201884_at	CEACAM5	carcinoembryonic antigen-related cell adhesion molecule 5	3.470	1.795	0.001	0.000
159	210754_s_at	LYN	v-yes-1 Yamaguchi sarcoma viral related oncogene homolog	3.468	1.794	0.000	0.000
160	219358_s_at	CENTA2	centaurin, alpha 2	3.458	1.790	0.000	0.000
162	205241_at	SCO2	SCO cytochrome oxidase deficient homolog 2 (yeast)	3.454	1.788	0.000	0.000
163	209792_s_at	KLK10	kallikrein-related peptidase 10	3.450	1.786	0.000	0.000

	Probe	Symbol	Description	FCH-Scalp-LS-NL	log2FCH-Scalp-LS-NL	p-Scalp-LS-NL	FDR-Scalp-LS-NL
164	211016_x_at	HSPA4	heat shock 70kDa protein 4	3.450	1.787	0.000	0.000
165	204750_s_at	DSC2	desmocollin 2	3.447	1.785	0.000	0.000
166	214549_x_at	SPRR1A	small proline-rich protein 1A	3.422	1.775	0.000	0.000
167	208965_s_at	PYHIN1	pyrin and HIN domain family, member 1	3.405	1.768	0.000	0.000
168	218943_s_at	DDX58	DEAD (Asp-Glu-Ala-Asp) box polypeptide 58	3.397	1.764	0.000	0.000
169	200727_s_at	ACTR2	ARP2 actin-related protein 2 homolog (yeast)	3.385	1.759	0.005	0.020
170	205401_at	AGPS	alkylglycerone phosphate synthase	3.385	1.759	0.000	0.000
171	204751_x_at	DSC2	desmocollin 2	3.366	1.751	0.001	0.000
172	217739_s_at	PBEF1	pre-B-cell colony enhancing factor 1	3.353	1.746	0.000	0.000
173	216243_s_at	IL1RN	interleukin 1 receptor antagonist	3.350	1.744	0.000	0.000
174	216316_x_at	GK	glycerol kinase	3.341	1.740	0.000	0.000
175	207018_s_at	RAB27B	RAB27B, member RAS oncogene family	3.338	1.739	0.000	0.000
176	208436_s_at	IRF7	interferon regulatory factor 7	3.327	1.734	0.000	0.000
177	210732_s_at	LGALS8	lectin, galactoside-binding, soluble, 8 (galectin 8)	3.307	1.726	0.000	0.000
178	201695_s_at	NP	nucleoside phosphorylase	3.294	1.720	0.000	0.000
179	206643_at	HAL	histidine ammonia-lyase	3.283	1.715	0.000	0.000
180	220800_s_at	TMOD3	tropomodulin 3 (ubiquitous)	3.280	1.713	0.001	0.000
181	218748_s_at	EXOC5	exocyst complex component 5	3.265	1.707	0.003	0.010
182	201860_s_at	PLAT	plasminogen activator, tissue	3.261	1.705	0.001	0.000
183	211612_s_at	IL13RA1	interleukin 13 receptor, alpha 1	3.258	1.704	0.002	0.010
184	219580_s_at	TMC5	transmembrane channel-like 5	3.244	1.698	0.000	0.000
185	206004_at	TGM3	transglutaminase 3 (E polypeptide, protein-glutamine-gamma-glutamyltransferase)	3.242	1.697	0.000	0.000
186	210119_at	KCNJ15	potassium inwardly-rectifying channel, subfamily J, member 15	3.242	1.697	0.000	0.000
187	210559_s_at	CDC2	cell division cycle 2, G1 to S and G2 to M	3.239	1.695	0.000	0.000
188	204128_s_at	RFC3	replication factor C (activator 1) 3, 38kDa	3.238	1.695	0.001	0.000
189	211195_s_at	TP63	tumor protein p63	3.235	1.694	0.001	0.000
190	209882_at	RIT1	Ras-like without CAAX 1	3.227	1.690	0.000	0.000
191	210834_s_at	PTGER3	prostaglandin E receptor 3 (subtype EP3)	3.216	1.685	0.000	0.000
192	209976_s_at	CYP2E1	cytochrome P450, family 2, subfamily E, polypeptide 1	3.211	1.683	0.000	0.000
193	218454_at	FLJ22662	hypothetical protein FLJ22662	3.210	1.682	0.000	0.000
194	214697_s_at	ROD1	ROD1 regulator of differentiation 1 (S. pombe)	3.209	1.682	0.000	0.000
195	217272_s_at	SERPINB13	serpin peptidase inhibitor, clade B (ovalbumin), member 13	3.209	1.682	0.000	0.000
196	218295_s_at	NUP50	nucleoporin 50kDa	3.201	1.678	0.000	0.000
197	39402_at	IL1B	interleukin 1, beta	3.194	1.675	0.004	0.020
198	219691_at	SAMD9	sterile alpha motif domain containing 9	3.187	1.672	0.000	0.000
199	201211_s_at	DDX3X	DEAD (Asp-Glu-Ala-Asp) box polypeptide 3, X-linked	3.173	1.666	0.002	0.010
200	208596_s_at	UGT1A3	UDP glucuronosyltransferase 1 family, polypeptide A3	3.168	1.663	0.000	0.000
201	203499_at	EPHA2	EPH receptor A2	3.153	1.657	0.000	0.000
202	209231_s_at	DCTN5	dynactin 5 (p25)	3.146	1.654	0.000	0.000
203	214446_at	ELL2	elongation factor, RNA polymerase II, 2	3.120	1.641	0.001	0.000
204	203946_s_at	ARG2	arginase, type II	3.113	1.638	0.000	0.000
205	206125_s_at	KLK8	kallikrein-related peptidase 8	3.104	1.634	0.000	0.000
206	219099_at	C12orf5	chromosome 12 open reading frame 5	3.101	1.633	0.000	0.000
207	202870_s_at	CDC20	cell division cycle 20 homolog (S. cerevisiae)	3.090	1.628	0.000	0.000
208	214070_s_at	ATP10B	ATPase, Class V, type 10B	3.084	1.625	0.000	0.000
209	205488_at	GZMA	granzyme A (granzyme 1, cytotoxic T-lymphocyte-associated serine esterase 3)	3.083	1.624	0.007	0.030
210	210148_at	HIPK3	homeodomain interacting protein kinase 3	3.081	1.623	0.009	0.040
211	219978_s_at	NUSAP1	nucleolar and spindle associated protein 1	3.062	1.614	0.000	0.000
212	210935_s_at	WDR1	WD repeat domain 1	3.058	1.613	0.001	0.000
213	213696_s_at	MED8	mediator complex subunit 8	3.057	1.612	0.000	0.000
214	202575_at	CRABP2	cellular retinoic acid binding protein 2	3.055	1.611	0.000	0.000
215	209825_s_at	UCK2	uridine-cytidine kinase 2	3.038	1.603	0.000	0.000
216	209093_s_at	GBA	glucosidase, beta; acid (includes glucosylceramidase)	3.028	1.598	0.000	0.000
217	206421_s_at	SERPINB7	serpin peptidase inhibitor, clade B (ovalbumin), member 7	3.022	1.595	0.000	0.000
218	218349_s_at	ZWILCH	Zwilch, kinetochore associated, homolog (Drosophila)	3.019	1.594	0.002	0.010
219	215465_at	ABCA12	ATP-binding cassette, sub-family A (ABC1), member 12	3.009	1.589	0.000	0.000
220	202067_s_at	LDLR	low density lipoprotein receptor (familial hypercholesterolemia)	3.007	1.588	0.000	0.000
221	201858_s_at	SRGN	serglycin	3.005	1.587	0.002	0.010
222	217109_at	MUC4	mucin 4, cell surface associated	2.997	1.584	0.006	0.030
223	212379_at	GART	phosphoribosylglycinamide formyltransferase, phosphoribosylglycinamide synthetase, phosphoribosylaminoimidazole synthet	2.996	1.583	0.000	0.000
224	206113_s_at	RAB5A	RAB5A, member RAS oncogene family	2.985	1.578	0.001	0.000
225	215125_s_at	UGT1A6	UDP glucuronosyltransferase 1 family, polypeptide A6	2.984	1.577	0.002	0.010
226	211361_s_at	SERPINB13	serpin peptidase inhibitor, clade B (ovalbumin), member 13	2.981	1.576	0.001	0.000
227	205798_at	IL7R	interleukin 7 receptor	2.973	1.572	0.011	0.040
228	211075_s_at	CD47	CD47 molecule	2.947	1.559	0.000	0.000
229	213548_s_at	CDV3	CDV3 homolog (mouse)	2.940	1.556	0.006	0.020
230	211801_x_at	MFN1	mitofusin 1	2.939	1.555	0.000	0.000
231	213875_x_at	C6orf62	chromosome 6 open reading frame 62	2.939	1.555	0.008	0.030
232	215395_x_at	TRY6	trypsinogen C	2.936	1.554	0.000	0.000
233	212587_s_at	PTPRC	protein tyrosine phosphatase, receptor type, C	2.934	1.553	0.003	0.010
234	206108_s_at	SFRS6	splicing factor, arginine/serine-rich 6	2.932	1.552	0.012	0.040
235	204601_at	N4BP1	Nedd4 binding protein 1	2.928	1.550	0.000	0.000
236	213457_at	MFHAS1	malignant fibrous histiocytoma amplified sequence 1	2.925	1.549	0.000	0.000
237	35820_at	GM2A	GM2 ganglioside activator	2.916	1.544	0.000	0.000
238	206429_at	F2RL1	coagulation factor II (thrombin) receptor-like 1	2.910	1.541	0.006	0.030
239	213577_at	SQLE	squalene epoxidase	2.908	1.540	0.000	0.000
240	219555_s_at	CENPN	centromere protein N	2.907	1.539	0.000	0.000
241	219684_at	RTP4	receptor (chemosensory) transporter protein 4	2.904	1.538	0.000	0.000
242	205159_at	CSF2RB	colony stimulating factor 2 receptor, beta, low-affinity (granulocyte-macrophage)	2.899	1.535	0.005	0.020
243	210915_x_at	TRBC1	T cell receptor beta constant 1	2.899	1.536	0.004	0.020
244	205569_at	LAMP3	lysosomal-associated membrane protein 3	2.897	1.535	0.000	0.000

	Probe	Symbol	Description	FCH-Scalp-LS-NL	log2FCH-Scalp-LS-NL	p-Scalp-LS-NL	FDR-Scalp-LS-NL
246	209629_s_at	NXT2	nuclear transport factor 2-like export factor 2	2.886	1.529	0.001	0.010
247	201469_s_at	SHC1	SHC (Src homology 2 domain containing) transforming protein 1	2.880	1.526	0.001	0.000
248	202479_s_at	TRIB2	tribbles homolog 2 (Drosophila)	2.876	1.524	0.000	0.000
249	206653_at	POLR3G	polymerase (RNA) III (DNA directed) polypeptide G (32kD)	2.871	1.522	0.000	0.000
250	212009_s_at	STIP1	stress-induced-phosphoprotein 1 (Hsp70/Hsp90-organizing protein)	2.868	1.520	0.007	0.030
251	220066_at	NOD2	nucleotide-binding oligomerization domain containing 2	2.867	1.520	0.000	0.000
252	211194_s_at	TP63	tumor protein p63	2.861	1.517	0.004	0.020
253	208744_x_at	HSPH1	heat shock 105kDa/110kDa protein 1	2.845	1.509	0.000	0.000
254	201101_s_at	BCLAF1	BCL2-associated transcription factor 1	2.843	1.507	0.001	0.000
255	213572_s_at	SERPINB1	serpin peptidase inhibitor, clade B (ovalbumin), member 1	2.843	1.507	0.001	0.000
257	202531_at	IRF1	interferon regulatory factor 1	2.841	1.507	0.000	0.000
258	202095_s_at	BIRC5	baculoviral IAP repeat-containing 5 (survivin)	2.831	1.502	0.000	0.000
259	207038_at	SLC16A6	solute carrier family 16, member 6 (monocarboxylic acid transporter 7)	2.829	1.500	0.000	0.000
260	201537_s_at	DUSP3	dual specificity phosphatase 3 (vaccinia virus phosphatase VH1-related)	2.822	1.497	0.000	0.000
261	208097_s_at	TXNDC1	thioredoxin domain containing 1	2.821	1.496	0.002	0.010
262	218340_s_at	UBE1L2	ubiquitin-activating enzyme E1-like 2	2.819	1.495	0.003	0.010
265	214279_s_at	NDRG2	NDRG family member 2	2.809	1.490	0.012	0.040
266	204891_s_at	LCK	lymphocyte-specific protein tyrosine kinase	2.786	1.478	0.009	0.030
267	217785_s_at	YKT6	YKT6 v-SNARE homolog (S. cerevisiae)	2.786	1.478	0.000	0.000
268	205916_at	S100A7	S100 calcium binding protein A7	2.782	1.476	0.000	0.000
269	204440_at	CD83	CD83 molecule	2.759	1.464	0.000	0.000
270	212659_s_at	IL1RN	interleukin 1 receptor antagonist	2.757	1.463	0.000	0.000
271	216598_s_at	CCL2	chemokine (C-C motif) ligand 2	2.755	1.462	0.000	0.000
272	207386_at	CYP7B1	cytochrome P450, family 7, subfamily B, polypeptide 1	2.748	1.459	0.000	0.000
273	213872_at	C6orf62	chromosome 6 open reading frame 62	2.745	1.457	0.006	0.020
274	212142_at	MCM4	minichromosome maintenance complex component 4	2.737	1.453	0.000	0.000
275	214681_at	GK	glycerol kinase	2.735	1.451	0.002	0.010
276	204174_at	ALOX5AP	arachidonate 5-lipoxygenase-activating protein	2.721	1.444	0.000	0.000
277	209456_s_at	FBXW11	F-box and WD repeat domain containing 11	2.711	1.439	0.000	0.000
278	220318_at	EPN3	epsin 3	2.706	1.436	0.000	0.000
279	209417_s_at	IFI35	interferon-induced protein 35	2.700	1.433	0.002	0.010
280	203148_s_at	TRIM14	tripartite motif-containing 14	2.695	1.430	0.000	0.000
281	205831_at	CD2	CD2 molecule	2.694	1.430	0.006	0.020
282	218817_at	SPCS3	signal peptidase complex subunit 3 homolog (S. cerevisiae)	2.693	1.429	0.004	0.020
283	203798_s_at	VSNL1	visinin-like 1	2.692	1.429	0.002	0.010
284	207238_s_at	PTPRC	protein tyrosine phosphatase, receptor type, C	2.689	1.427	0.005	0.020
285	214975_s_at	MTMR1	myotubularin related protein 1	2.689	1.427	0.010	0.040
286	205847_at	PRSS22	protease, serine, 22	2.686	1.425	0.000	0.000
287	200730_s_at	PTP4A1	protein tyrosine phosphatase type IVA, member 1	2.676	1.420	0.001	0.000
288	213523_at	CCNE1	cyclin E1	2.663	1.413	0.000	0.000
289	215236_s_at	PICALM	phosphatidylinositol binding clathrin assembly protein	2.663	1.413	0.006	0.030
290	217192_s_at	PRDM1	PR domain containing 1, with ZNF domain	2.659	1.411	0.000	0.000
291	204962_s_at	CENPA	centromere protein A	2.657	1.410	0.000	0.000
292	205767_at	EREG	epiregulin	2.651	1.407	0.002	0.010
293	217294_s_at	ENO1	enolase 1, (alpha)	2.651	1.407	0.001	0.000
294	214088_s_at	FUT3	fucosyltransferase 3 (galactoside 3(4)-L-fucosyltransferase, Lewis blood group)	2.647	1.404	0.000	0.000
295	219079_at	CYB5R4	cytochrome b5 reductase 4	2.632	1.396	0.000	0.000
296	205349_at	GNA15	guanine nucleotide binding protein (G protein), alpha 15 (Gq class)	2.627	1.393	0.000	0.000
297	213988_s_at	SAT1	spermidine/spermine N1-acetyltransferase 1	2.625	1.393	0.000	0.000
298	204527_at	MYO5A	myosin VA (heavy chain 12, myoxin)	2.624	1.392	0.000	0.000
299	210018_x_at	MALT1	mucosa associated lymphoid tissue lymphoma translocation gene 1	2.607	1.382	0.000	0.000
300	203764_at	DLG7	discs, large homolog 7 (Drosophila)	2.601	1.379	0.003	0.010
301	200769_s_at	MAT2A	methionine adenosyltransferase II, alpha	2.600	1.379	0.007	0.030
302	212141_at	MCM4	minichromosome maintenance complex component 4	2.600	1.378	0.000	0.000
303	212737_at	GM2A	GM2 ganglioside activator	2.599	1.378	0.000	0.000
304	219918_s_at	ASPM	asp (abnormal spindle) homolog, microcephaly associated (Drosophila)	2.596	1.376	0.001	0.000
305	205909_at	POLE2	polymerase (DNA directed), epsilon 2 (p59 subunit)	2.588	1.372	0.000	0.000
306	204420_at	FOSL1	FOS-like antigen 1	2.586	1.370	0.001	0.000
307	206036_s_at	REL	v-rel reticuloendotheliosis viral oncogene homolog (avian)	2.583	1.369	0.000	0.000
308	206553_at	OAS2	2'-5'-oligoadenylate synthetase 2, 69/71kDa	2.582	1.369	0.000	0.000
309	201048_x_at	RAB6A	RAB6A, member RAS oncogene family	2.581	1.368	0.001	0.000
310	1431_at	CYP2E1	cytochrome P450, family 2, subfamily E, polypeptide 1	2.577	1.366	0.000	0.000
311	212978_at	LRRC8B	leucine rich repeat containing 8 family, member B	2.577	1.366	0.000	0.000
312	212022_s_at	MKI67	antigen identified by monoclonal antibody Ki-67	2.571	1.362	0.000	0.000
313	210852_s_at	AASS	aminoadipate-semialdehyde synthase	2.568	1.360	0.000	0.000
314	218355_at	KIF4A	kinesin family member 4A	2.568	1.361	0.000	0.000
315	202659_at	PSMB10	proteasome (prosome, macropain) subunit, beta type, 10	2.567	1.360	0.000	0.000
316	203256_at	CDH3	cadherin 3, type 1, P-cadherin (placental)	2.563	1.358	0.000	0.000
317	217028_at	CXCR4	chemokine (C-X-C motif) receptor 4	2.560	1.356	0.000	0.000
318	218562_s_at	TMEM57	transmembrane protein 57	2.560	1.356	0.000	0.000
320	209236_at	SLC23A2	solute carrier family 23 (nucleobase transporters), member 2	2.558	1.355	0.000	0.000
321	208309_s_at	MALT1	mucosa associated lymphoid tissue lymphoma translocation gene 1	2.553	1.352	0.000	0.000
322	208116_s_at	MAN1A1	mannosidase, alpha, class 1A, member 1	2.551	1.351	0.009	0.040
323	217738_at	PBEF1	pre-B-cell colony enhancing factor 1	2.546	1.348	0.000	0.000
324	219836_at	ZBED2	zinc finger, BED-type containing 2	2.545	1.348	0.000	0.000
325	212902_at	SEC24A	SEC24 related gene family, member A (S. cerevisiae)	2.544	1.347	0.001	0.000
326	217202_s_at	GLUL	glutamate-ammonia ligase (glutamine synthetase)	2.542	1.346	0.002	0.010
327	205895_s_at	NOLC1	nucleolar and coiled-body phosphoprotein 1	2.536	1.343	0.000	0.000
328	212769_at	TLE3	transducin-like enhancer of split 3 (E(sp1) homolog, Drosophila)	2.532	1.340	0.000	0.000
329	216399_s_at	SCAPER	S phase cyclin A-associated protein in the ER	2.532	1.341	0.000	0.000
330	203665_at	HMOX1	heme oxygenase (decycling) 1	2.531	1.340	0.000	0.000

	Probe	Symbol	Description	FCH-Scalp-LS-NL	log2FCH-Scalp-LS-NL	p-Scalp-LS-NL	FDR-Scalp-LS-NL
331	201523_x_at	UBE2N	ubiquitin-conjugating enzyme E2N (UBC13 homolog, yeast)	2.526	1.337	0.000	0.000
332	212290_at	SLC7A1	solute carrier family 7 (cationic amino acid transporter, y+ system), member 1	2.523	1.335	0.000	0.000
333	209714_s_at	CDKN3	cyclin-dependent kinase inhibitor 3 (CDK2-associated dual specificity phosphatase)	2.521	1.334	0.001	0.000
334	203716_s_at	DPP4	dipeptidyl-peptidase 4 (CD26, adenosine deaminase complexing protein 2)	2.506	1.325	0.010	0.040
335	201890_at	RRM2	ribonucleotide reductase M2 polypeptide	2.504	1.324	0.000	0.000
336	217763_s_at	RAB31	RAB31, member RAS oncogene family	2.500	1.322	0.000	0.000
337	210357_s_at	SMOX	spermine oxidase	2.499	1.321	0.000	0.000
338	204698_at	ISG20	interferon stimulated exonuclease gene 20kDa	2.494	1.319	0.000	0.000
339	202083_s_at	SEC14L1	SEC14-like 1 (S. cerevisiae)	2.486	1.314	0.000	0.000
340	200604_s_at	PRKAR1A	protein kinase, cAMP-dependent, regulatory, type I, alpha (tissue specific extinguisher 1)	2.483	1.312	0.006	0.030
341	201663_s_at	SMC4	structural maintenance of chromosomes 4	2.483	1.312	0.000	0.000
342	216125_s_at	RANBP9	RAN binding protein 9	2.482	1.312	0.000	0.000
343	202917_s_at	S100A8	S100 calcium binding protein A8	2.481	1.311	0.001	0.000
344	203418_at	CCNA2	cyclin A2	2.481	1.311	0.000	0.000
345	219267_at	GLTP	glycolipid transfer protein	2.481	1.311	0.000	0.000
346	203768_s_at	STS	steroid sulfatase (microsomal), isozyme S	2.478	1.309	0.001	0.000
347	201992_s_at	KIF5B	kinesin family member 5B	2.476	1.308	0.000	0.000
348	202533_s_at	DHFR	dihydrofolate reductase	2.475	1.308	0.000	0.000
349	209408_at	KIF2C	kinesin family member 2C	2.475	1.308	0.000	0.000
350	212365_at	MYO1B	myosin IB	2.475	1.307	0.000	0.000
351	203767_s_at	STS	steroid sulfatase (microsomal), isozyme S	2.473	1.306	0.000	0.000
352	203128_at	SPTLC2	serine palmitoyltransferase, long chain base subunit 2	2.472	1.306	0.000	0.000
353	203740_at	MPHOSPH6	M-phase phosphoprotein 6	2.471	1.305	0.000	0.000
354	206472_s_at	TLE3	transducin-like enhancer of split 3 (E(sp1) homolog, Drosophila)	2.471	1.305	0.000	0.000
355	206529_x_at	SLC26A4	solute carrier family 26, member 4	2.469	1.304	0.007	0.030
356	205419_at	EBI2	Epstein-Barr virus induced gene 2 (lymphocyte-specific G protein-coupled receptor)	2.463	1.301	0.003	0.010
357	219148_at	PBK	PDZ binding kinase	2.461	1.299	0.001	0.010
358	213131_at	OLFM1	olfactomedin 1	2.457	1.297	0.000	0.000
359	206748_s_at	SPAG9	sperm associated antigen 9	2.455	1.296	0.000	0.000
360	209853_s_at	PSME3	proteasome (prosome, macropain) activator subunit 3 (PA28 gamma; Ki)	2.454	1.295	0.000	0.000
361	204033_at	TRIP13	thyroid hormone receptor interactor 13	2.452	1.294	0.000	0.000
362	219915_s_at	SLC16A10	solute carrier family 16, member 10 (aromatic amino acid transporter)	2.452	1.294	0.000	0.000
363	206562_s_at	CSNK1A1	casein kinase 1, alpha 1	2.450	1.293	0.000	0.000
364	221903_s_at	CYLD	cylindromatosis (turban tumor syndrome)	2.442	1.288	0.000	0.000
365	205246_at	PEX13	peroxisome biogenesis factor 13	2.437	1.285	0.002	0.010
366	212107_s_at	DHX9	DEAH (Asp-Glu-Ala-His) box polypeptide 9	2.436	1.284	0.008	0.030
367	203560_at	GGH	gamma-glutamyl hydrolase (conjugase, folylpolygammaglutamyl hydrolase)	2.432	1.282	0.000	0.000
368	212016_s_at	PTBP1	polypyrimidine tract binding protein 1	2.429	1.280	0.003	0.010
369	205015_s_at	TGFA	transforming growth factor, alpha	2.428	1.280	0.000	0.000
370	214226_at	POL3S	polyserase 3	2.427	1.279	0.002	0.010
371	218542_at	CEP55	centrosomal protein 55kDa	2.426	1.278	0.001	0.000
372	220944_at	PGLYRP4	peptidoglycan recognition protein 4	2.426	1.278	0.000	0.000
373	204393_s_at	ACPP	acid phosphatase, prostate	2.423	1.277	0.000	0.000
374	201476_s_at	RRM1	ribonucleotide reductase M1 polypeptide	2.419	1.275	0.002	0.010
375	211796_s_at	TRBC1	T cell receptor beta constant 1	2.417	1.273	0.005	0.020
376	208567_s_at	KCNJ12	potassium inwardly-rectifying channel, subfamily J, member 12	2.412	1.270	0.003	0.020
377	201732_s_at	CLCN3	chloride channel 3	2.400	1.263	0.000	0.000
378	208992_s_at	STAT3	signal transducer and activator of transcription 3 (acute-phase response factor)	2.398	1.262	0.000	0.000
379	204641_at	NEK2	NIMA (never in mitosis gene a)-related kinase 2	2.396	1.261	0.011	0.040
380	218498_s_at	ERO1L	ERO1-like (S. cerevisiae)	2.396	1.261	0.001	0.000
381	212514_x_at	DDX3X	DEAD (Asp-Glu-Ala-Asp) box polypeptide 3, X-linked	2.393	1.259	0.000	0.000
382	210001_s_at	SOCS1	suppressor of cytokine signaling 1	2.386	1.254	0.000	0.000
383	202236_s_at	SLC16A1	solute carrier family 16, member 1 (monocarboxylic acid transporter 1)	2.384	1.253	0.000	0.000
384	219316_s_at	FLVCR2	feline leukemia virus subgroup C cellular receptor family, member 2	2.380	1.251	0.000	0.000
385	206094_x_at	UGT1A6	UDP glucuronosyltransferase 1 family, polypeptide A6	2.374	1.247	0.002	0.010
386	220865_s_at	PDSS1	prenyl (decaprenyl) diphosphate synthase, subunit 1	2.373	1.247	0.010	0.040
387	212143_s_at	IGFBP3	insulin-like growth factor binding protein 3	2.372	1.246	0.001	0.000
388	201196_s_at	AMD1	adenosylmethionine decarboxylase 1	2.371	1.246	0.000	0.000
389	208433_s_at	LRP8	low density lipoprotein receptor-related protein 8, apolipoprotein e receptor	2.370	1.245	0.000	0.000
390	204747_at	IFIT3	interferon-induced protein with tetratricopeptide repeats 3	2.364	1.241	0.000	0.000
391	205064_at	SPRR1B	small proline-rich protein 1B (cornifin)	2.364	1.241	0.000	0.000
392	209744_x_at	ITCH	itchy homolog E3 ubiquitin protein ligase (mouse)	2.364	1.241	0.000	0.000
393	205778_at	KLK7	kallikrein-related peptidase 7	2.357	1.237	0.000	0.000
394	210589_s_at	GBA	glucosidase, beta; acid (includes glucosylceramidase)	2.357	1.237	0.000	0.000
395	206932_at	CH25H	cholesterol 25-hydroxylase	2.350	1.233	0.004	0.020
396	219789_at	NPR3	natriuretic peptide receptor C/guanylate cyclase C (atrionatriuretic peptide receptor C)	2.349	1.232	0.003	0.010
397	214076_at	GFOD2	glucose-fructose oxidoreductase domain containing 2	2.344	1.229	0.000	0.000
398	201946_s_at	CCT2	chaperonin containing TCP1, subunit 2 (beta)	2.343	1.229	0.001	0.010
399	204768_s_at	FEN1	flap structure-specific endonuclease 1	2.343	1.228	0.000	0.000
400	203233_at	IL4R	interleukin 4 receptor	2.341	1.227	0.000	0.000
401	219858_s_at	FLJ20160	FLJ20160 protein	2.341	1.227	0.002	0.010
402	204057_at	IRF8	interferon regulatory factor 8	2.340	1.227	0.001	0.000
403	205595_at	DSG3	desmoglein 3 (pemphigus vulgaris antigen)	2.340	1.227	0.000	0.000
404	219856_at	C1orf116	chromosome 1 open reading frame 116	2.337	1.225	0.000	0.000
405	212720_at	PAPOLA	poly(A) polymerase alpha	2.332	1.222	0.005	0.020
406	213937_s_at	FTSJ1	FtsJ homolog 1 (E. coli)	2.330	1.220	0.000	0.000
407	220285_at	FAM108B1	family with sequence similarity 108, member B1	2.328	1.219	0.001	0.000
408	204908_s_at	BCL3	B-cell CLL/lymphoma 3	2.325	1.217	0.000	0.000
409	39249_at	AQP3	aquaporin 3 (Gill blood group)	2.321	1.215	0.000	0.000
410	209257_s_at	SMC3	structural maintenance of chromosomes 3	2.319	1.214	0.002	0.010
411	204881_s_at	UGCG	UDP-glucose ceramide glucosyltransferase	2.312	1.209	0.000	0.000

	Probe	Symbol	Description	FCH-Scalp-LS-NL	log2FCH-Scalp-LS-NL	p-Scalp-LS-NL	FDR-Scalp-LS-NL
412	220013_at	ABHD9	abhydrolase domain containing 9	2.312	1.209	0.000	0.000
413	205990_s_at	WNT5A	wingless-type MMTV integration site family, member 5A	2.311	1.209	0.005	0.020
414	211113_s_at	ABCG1	ATP-binding cassette, sub-family G (WHITE), member 1	2.298	1.200	0.000	0.000
415	208934_s_at	LGALS8	lectin, galactoside-binding, soluble, 8 (galectin 8)	2.296	1.199	0.000	0.000
416	217234_s_at	VIL2	villin 2 (ezrin)	2.292	1.196	0.002	0.010
417	210594_x_at	MPZL1	myelin protein zero-like 1	2.285	1.192	0.000	0.000
418	219187_at	FKBPL	FK506 binding protein like	2.285	1.192	0.000	0.000
419	202129_s_at	RIOK3	RIO kinase 3 (yeast)	2.280	1.189	0.001	0.000
420	212460_at	C14orf147	chromosome 14 open reading frame 147	2.280	1.189	0.000	0.000
421	209975_at	CYP2E1	cytochrome P450, family 2, subfamily E, polypeptide 1	2.279	1.188	0.000	0.000
422	214895_s_at	ADAM10	ADAM metallopeptidase domain 10	2.279	1.188	0.005	0.020
423	208651_x_at	CD24	CD24 molecule	2.278	1.188	0.000	0.000
424	202446_s_at	PLSCR1	phospholipid scramblase 1	2.274	1.185	0.000	0.000
425	205170_at	STAT2	signal transducer and activator of transcription 2, 113kDa	2.271	1.183	0.002	0.010
426	210827_s_at	ELF3	E74-like factor 3 (ets domain transcription factor, epithelial-specific)	2.267	1.181	0.001	0.000
427	217370_x_at	FUS	fusion (involved in t(12;16) in malignant liposarcoma)	2.267	1.181	0.001	0.000
428	201489_at	PPIF	peptidylprolyl isomerase F (cyclophilin F)	2.266	1.180	0.000	0.000
429	203777_s_at	RPS6KB2	ribosomal protein S6 kinase, 70kDa, polypeptide 2	2.264	1.179	0.000	0.000
430	202604_x_at	ADAM10	ADAM metallopeptidase domain 10	2.260	1.177	0.001	0.010
431	201504_s_at	TSN	translin	2.254	1.172	0.001	0.000
432	202269_x_at	GBP1	guanylate binding protein 1, interferon-inducible, 67kDa	2.250	1.170	0.000	0.000
433	207992_s_at	AMPD3	adenosine monophosphate deaminase (isoform E)	2.249	1.169	0.000	0.000
434	202625_at	LYN	v-yes-1 Yamaguchi sarcoma viral related oncogene homolog	2.246	1.167	0.000	0.000
435	218900_at	CNNM4	cyclin M4	2.246	1.167	0.000	0.000
436	213193_x_at	TRBC1	T cell receptor beta constant 1	2.245	1.167	0.006	0.020
437	202058_s_at	KPNA1	karyopherin alpha 1 (importin alpha 5)	2.243	1.166	0.003	0.010
438	210833_at	PTGER3	prostaglandin E receptor 3 (subtype EP3)	2.243	1.165	0.007	0.030
439	203974_at	HDHD1A	haloacid dehalogenase-like hydrolase domain containing 1A	2.241	1.164	0.000	0.000
440	208351_s_at	MAPK1	mitogen-activated protein kinase 1	2.240	1.164	0.001	0.000
441	205394_at	CHEK1	CHK1 checkpoint homolog (S. pombe)	2.234	1.160	0.003	0.010
442	204769_s_at	TAP2	transporter 2, ATP-binding cassette, sub-family B (MDR/TAP)	2.226	1.155	0.001	0.010
443	219095_at	PLA2G4B	phospholipase A2, group IVB (cytosolic)	2.226	1.154	0.000	0.000
444	37152_at	PPARD	peroxisome proliferator-activated receptor delta	2.225	1.154	0.000	0.000
445	211622_s_at	ARF3	ADP-ribosylation factor 3	2.224	1.153	0.004	0.020
446	217173_s_at	LDLR	low density lipoprotein receptor (familial hypercholesterolemia)	2.220	1.150	0.003	0.010
447	206166_s_at	CLCA2	chloride channel, calcium activated, family member 2	2.218	1.149	0.000	0.000
448	209055_s_at	CDC5L	CDC5 cell division cycle 5-like (S. pombe)	2.215	1.147	0.005	0.020
449	208721_s_at	ANAPC5	anaphase promoting complex subunit 5	2.212	1.145	0.004	0.020
450	206686_at	PDK1	pyruvate dehydrogenase kinase, isozyme 1	2.211	1.145	0.001	0.000
451	202503_s_at	KIAA0101	KIAA0101	2.209	1.144	0.003	0.010
452	211672_s_at	ARPC4	actin related protein 2/3 complex, subunit 4, 20kDa	2.209	1.143	0.004	0.020
453	209237_s_at	SLC23A2	solute carrier family 23 (nucleobase transporters), member 2	2.208	1.143	0.011	0.040
454	204170_s_at	CKS2	CDC28 protein kinase regulatory subunit 2	2.207	1.142	0.000	0.000
455	218951_s_at	PLCXD1	phosphatidylinositol-specific phospholipase C, X domain containing 1	2.207	1.142	0.000	0.000
456	219015_s_at	ALG13	asparagine-linked glycosylation 13 homolog (S. cerevisiae)	2.207	1.142	0.002	0.010
457	206278_at	PTAFR	platelet-activating factor receptor	2.205	1.141	0.000	0.000
458	214382_at	UNC93A	unc-93 homolog A (C. elegans)	2.205	1.141	0.008	0.030
459	216388_s_at	LTB4R	leukotriene B4 receptor	2.205	1.141	0.000	0.000
460	209159_s_at	NDRG4	NDRG family member 4	2.204	1.140	0.000	0.000
461	211090_s_at	PRPF4B	PRP4 pre-mRNA processing factor 4 homolog B (yeast)	2.203	1.140	0.007	0.030
462	202787_s_at	MAPKAPK3	mitogen-activated protein kinase-activated protein kinase 3	2.199	1.137	0.001	0.000
463	216689_x_at	ARHGAP1	Rho GTPase activating protein 1	2.198	1.136	0.004	0.020
464	220941_s_at	C21orf91	chromosome 21 open reading frame 91	2.196	1.135	0.000	0.000
465	214959_s_at	API5	apoptosis inhibitor 5	2.195	1.134	0.001	0.000
466	205538_at	CORO2A	coronin, actin binding protein, 2A	2.191	1.131	0.000	0.000
467	215760_s_at	SBNO2	strawberry notch homolog 2 (Drosophila)	2.191	1.131	0.005	0.020
468	201720_s_at	LAPTM5	lysosomal associated multispanning membrane protein 5	2.185	1.127	0.006	0.030
469	221521_s_at	GINS2	GINS complex subunit 2 (Psf2 homolog)	2.185	1.128	0.000	0.000
470	202199_s_at	SRPK1	SFRS protein kinase 1	2.183	1.126	0.000	0.000
471	206303_s_at	NUDT4	nudix (nucleoside diphosphate linked moiety X)-type motif 4	2.181	1.125	0.003	0.010
472	207223_s_at	ROD1	ROD1 regulator of differentiation 1 (S. pombe)	2.180	1.124	0.001	0.000
473	209126_x_at	KRT6B	keratin 6B	2.179	1.124	0.001	0.000
474	266_s_at	CD24	CD24 molecule	2.179	1.124	0.000	0.000
475	209949_at	NCF2	neutrophil cytosolic factor 2 (65kDa, chronic granulomatous disease, autosomal 2)	2.178	1.123	0.002	0.010
476	214838_at	SFT2D2	SFT2 domain containing 2	2.177	1.123	0.000	0.000
477	201571_s_at	DCTD	dCMP deaminase	2.175	1.121	0.000	0.000
478	209247_s_at	ABCF2	ATP-binding cassette, sub-family F (GCN20), member 2	2.170	1.118	0.000	0.000
479	60474_at	C20orf42	chromosome 20 open reading frame 42	2.169	1.117	0.000	0.000
480	204606_at	CCL21	chemokine (C-C motif) ligand 21	2.167	1.116	0.010	0.040
481	204331_s_at	MRPS12	mitochondrial ribosomal protein S12	2.165	1.114	0.000	0.000
482	205402_x_at	PRSS2	protease, serine, 2 (trypsin 2)	2.164	1.114	0.000	0.000
483	200641_s_at	YWHAZ	tyrosine 3-monooxygenase/tryptophan 5-monooxygenase activation protein, zeta polypeptide	2.160	1.111	0.001	0.010
484	205220_at	GPR109B	G protein-coupled receptor 109B	2.160	1.111	0.000	0.000
485	217881_s_at	CDC27	cell division cycle 27 homolog (S. cerevisiae)	2.159	1.110	0.004	0.020
486	208867_s_at	CSNK1A1	casein kinase 1, alpha 1	2.158	1.110	0.000	0.000
487	211150_s_at	DLAT	dihydrolipoamide S-acetyltransferase (E2 component of pyruvate dehydrogenase complex)	2.158	1.110	0.001	0.000
488	200987_x_at	PSME3	proteasome (prosome, macropain) activator subunit 3 (PA28 gamma; Ki)	2.156	1.108	0.000	0.000
489	211547_s_at	PAFAH1B1	platelet-activating factor acetylhydrolase, isoform Ib, alpha subunit 45kDa	2.156	1.108	0.012	0.040
490	214580_x_at	KRT6A	keratin 6A	2.156	1.108	0.001	0.010
491	214544_s_at	SNAP23	synaptosomal-associated protein, 23kDa	2.150	1.105	0.008	0.030
492	203072_at	MYO1E	myosin IE	2.149	1.104	0.000	0.000

	Probe	Symbol	Description	FCH-Scalp-LS-NL	log2FCH-Scalp-LS-NL	p-Scalp-LS-NL	FDR-Scalp-LS-NL
493	220104_at	ZC3HAV1	zinc finger CCCH-type, antiviral 1	2.148	1.103	0.000	0.000
494	203964_at	NMI	N-myc (and STAT) interactor	2.146	1.102	0.000	0.000
495	217448_s_at	LOC285412	similar to Epidermal Langerhans cell protein LCP1	2.146	1.101	0.000	0.000
496	211681_s_at	PDLIM5	PDZ and LIM domain 5	2.141	1.099	0.001	0.010
497	218796_at	C20orf42	chromosome 20 open reading frame 42	2.139	1.097	0.000	0.000
498	205842_s_at	JAK2	Janus kinase 2 (a protein tyrosine kinase)	2.138	1.096	0.001	0.010
499	202705_at	CCNB2	cyclin B2	2.136	1.095	0.000	0.000
500	203780_at	MPZL2	myelin protein zero-like 2	2.136	1.095	0.003	0.020
501	204465_s_at	INA	internexin neuronal intermediate filament protein, alpha	2.135	1.094	0.000	0.000
502	217496_s_at	IDE	insulin-degrading enzyme	2.133	1.093	0.000	0.000
503	221477_s_at	MGC5618	hypothetical protein MGC5618	2.132	1.092	0.000	0.000
504	219503_s_at	TMEM40	transmembrane protein 40	2.130	1.091	0.000	0.000
505	201888_s_at	IL13RA1	interleukin 13 receptor, alpha 1	2.128	1.090	0.008	0.030
506	206109_at	FUT1	fucosyltransferase 1 (galactoside 2-alpha-L-fucosyltransferase, H blood group)	2.127	1.089	0.001	0.010
507	221779_at	MICALL1	MICAL-like 1	2.126	1.088	0.000	0.000
508	202453_s_at	GTF2H1	general transcription factor IIH, polypeptide 1, 62kDa	2.124	1.087	0.001	0.000
509	213796_at	SPRR1A	small proline-rich protein 1A	2.123	1.086	0.004	0.020
510	200648_s_at	GLUL	glutamate-ammonia ligase (glutamine synthetase)	2.122	1.086	0.001	0.010
511	218719_s_at	GINS3	GINS complex subunit 3 (Psf3 homolog)	2.117	1.082	0.000	0.000
512	207008_at	IL8RB	interleukin 8 receptor, beta	2.115	1.081	0.005	0.020
513	220620_at	CRCT1	cysteine-rich C-terminal 1	2.114	1.080	0.001	0.000
514	208018_s_at	HCK	hemopoietic cell kinase	2.112	1.079	0.007	0.030
515	218755_at	KIF20A	kinesin family member 20A	2.112	1.078	0.000	0.000
516	211578_s_at	RPS6KB1	ribosomal protein S6 kinase, 70kDa, polypeptide 1	2.107	1.075	0.000	0.000
517	204224_s_at	GCH1	GTP cyclohydrolase 1 (dopa-responsive dystonia)	2.106	1.074	0.001	0.000
518	206337_at	CCR7	chemokine (C-C motif) receptor 7	2.104	1.073	0.003	0.010
519	212521_s_at	PDE8A	phosphodiesterase 8A	2.104	1.073	0.000	0.000
520	218073_s_at	TMEM48	transmembrane protein 48	2.097	1.068	0.000	0.000
521	209310_s_at	CASP4	caspase 4, apoptosis-related cysteine peptidase	2.091	1.064	0.000	0.000
522	213927_at	MAP3K9	mitogen-activated protein kinase kinase kinase 9	2.090	1.063	0.002	0.010
523	213599_at	OIP5	Opa interacting protein 5	2.089	1.063	0.000	0.000
524	217764_s_at	RAB31	RAB31, member RAS oncogene family	2.088	1.062	0.000	0.000
525	216379_x_at	CD24	CD24 molecule	2.086	1.060	0.000	0.000
526	215707_s_at	PRNP	prion protein (p27-30) (Creutzfeldt-Jakob disease, Gerstmann-Strausler-Scheinker syndrome, fatal familial insomnia)	2.084	1.060	0.003	0.010
527	208478_s_at	BAX	BCL2-associated X protein	2.082	1.058	0.000	0.000
528	215984_s_at	ARFRP1	ADP-ribosylation factor related protein 1	2.081	1.057	0.003	0.020
529	221220_s_at	SCYL2	SCY1-like 2 (S. cerevisiae)	2.081	1.058	0.009	0.040
530	220413_at	SLC39A2	solute carrier family 39 (zinc transporter), member 2	2.077	1.054	0.001	0.000
531	33304_at	ISG20	interferon stimulated exonuclease gene 20kDa	2.077	1.054	0.000	0.000
532	204825_at	MELK	maternal embryonic leucine zipper kinase	2.072	1.051	0.000	0.000
533	202069_s_at	IDH3A	isocitrate dehydrogenase 3 (NAD+) alpha	2.070	1.050	0.000	0.000
534	204638_at	ACP5	acid phosphatase 5, tartrate resistant	2.068	1.048	0.001	0.000
535	216574_s_at	RPE	ribulose-5-phosphate-3-epimerase	2.068	1.048	0.002	0.010
536	217755_at	HN1	hematological and neurological expressed 1	2.068	1.048	0.000	0.000
537	219680_at	NLRX1	NLR family member X1	2.067	1.048	0.000	0.000
538	202270_at	GBP1	guanylate binding protein 1, interferon-inducible, 67kDa	2.065	1.046	0.006	0.020
539	209373_at	MALL	mal, T-cell differentiation protein-like	2.064	1.046	0.000	0.000
540	209954_x_at	SS18	synovial sarcoma translocation, chromosome 18	2.061	1.043	0.001	0.000
541	214829_at	AASS	aminoadipate-semialdehyde synthase	2.061	1.043	0.000	0.000
542	204715_at	PANX1	pannexin 1	2.057	1.041	0.000	0.000
543	210039_s_at	PRKCQ	protein kinase C, theta	2.057	1.041	0.001	0.000
544	203328_x_at	IDE	insulin-degrading enzyme	2.054	1.039	0.000	0.000
545	220599_s_at	CARD14	caspase recruitment domain family, member 14	2.054	1.039	0.000	0.000
546	209372_x_at	TUBB2A	tubulin, beta 2A	2.053	1.038	0.011	0.040
547	205196_s_at	AP1S1	adaptor-related protein complex 1, sigma 1 subunit	2.052	1.037	0.001	0.000
548	216915_s_at	PTPN12	protein tyrosine phosphatase, non-receptor type 12	2.051	1.036	0.011	0.040
549	203573_s_at	RABGGTA	Rab geranylgeranyltransferase, alpha subunit	2.050	1.035	0.000	0.000
550	207749_s_at	PPP2R3A	protein phosphatase 2 (formerly 2A), regulatory subunit B'', alpha	2.050	1.036	0.000	0.000
551	208885_at	LCP1	lymphocyte cytosolic protein 1 (L-plastin)	2.050	1.036	0.000	0.000
552	214698_at	ROD1	ROD1 regulator of differentiation 1 (S. pombe)	2.050	1.036	0.000	0.000
553	214640_at	UNC93A	unc-93 homolog A (C. elegans)	2.049	1.035	0.000	0.000
554	214499_s_at	BCLAF1	BCL2-associated transcription factor 1	2.046	1.033	0.001	0.000
555	211806_s_at	KCNJ15	potassium inwardly-rectifying channel, subfamily J, member 15	2.042	1.030	0.001	0.000
556	201649_at	UBE2L6	ubiquitin-conjugating enzyme E2L 6	2.041	1.029	0.000	0.000
557	221666_s_at	PYCARD	PYD and CARD domain containing	2.041	1.029	0.000	0.000
558	205282_at	LRP8	low density lipoprotein receptor-related protein 8, apolipoprotein e receptor	2.039	1.028	0.000	0.000
559	207085_x_at	CSF2RA	colony stimulating factor 2 receptor, alpha, low-affinity (granulocyte-macrophage)	2.039	1.028	0.004	0.020
560	202068_s_at	LDLR	low density lipoprotein receptor (familial hypercholesterolemia)	2.038	1.027	0.000	0.000
561	205287_s_at	TFAP2C	transcription factor AP-2 gamma (activating enhancer binding protein 2 gamma)	2.038	1.027	0.008	0.030
562	207622_s_at	ABCF2	ATP-binding cassette, sub-family F (GCN20), member 2	2.038	1.027	0.000	0.000
563	217960_s_at	TOMM22	translocase of outer mitochondrial membrane 22 homolog (yeast)	2.035	1.025	0.000	0.000
564	201733_at	CLCN3	chloride channel 3	2.033	1.024	0.000	0.000
565	202988_s_at	RGS1	regulator of G-protein signaling 1	2.031	1.023	0.004	0.020
566	201735_s_at	CLCN3	chloride channel 3	2.030	1.021	0.000	0.000
567	200628_s_at	WARS	tryptophanyl-tRNA synthetase	2.029	1.021	0.001	0.000
568	219209_at	IFIH1	interferon induced with helicase C domain 1	2.029	1.021	0.001	0.010
569	203476_at	TPBG	trophoblast glycoprotein	2.025	1.018	0.000	0.000
570	204162_at	NDC80	NDC80 homolog, kinetochore complex component (S. cerevisiae)	2.024	1.017	0.001	0.000
571	204147_s_at	TFDP1	transcription factor Dp-1	2.022	1.016	0.000	0.000
572	209188_x_at	DR1	down-regulator of transcription 1, TBP-binding (negative cofactor 2)	2.022	1.016	0.000	0.000
573	212657_s_at	IL1RN	interleukin 1 receptor antagonist	2.022	1.016	0.000	0.000

	Probe	Symbol	Description	FCH-Scalp-LS-NL	log2FCH-Scalp-LS-NL	p-Scalp-LS-NL	FDR-Scalp-LS-NL
574	203747_at	AQP3	aquaporin 3 (Gill blood group)	2.019	1.013	0.000	0.000
575	207126_x_at	UGT1A1	UDP glucuronosyltransferase 1 family, polypeptide A1	2.019	1.014	0.005	0.020
576	210056_at	RND1	Rho family GTPase 1	2.019	1.014	0.010	0.040
577	201686_x_at	API5	apoptosis inhibitor 5	2.018	1.013	0.002	0.010
578	207540_s_at	SYK	spleen tyrosine kinase	2.015	1.011	0.000	0.000
579	207291_at	PRRG4	proline rich Gla (G-carboxyglutamic acid) 4 (transmembrane)	2.014	1.010	0.002	0.010
580	206193_s_at	CDSN	corneodesmosin	2.010	1.007	0.006	0.030
581	206276_at	LY6D	lymphocyte antigen 6 complex, locus D	2.008	1.006	0.000	0.000
582	213416_at	CERKL	ceramide kinase-like	2.007	1.005	0.010	0.040
583	221487_s_at	ENSA	endosulfine alpha	2.003	1.002	0.005	0.020
584	209053_s_at	WHSC1	Wolf-Hirschhorn syndrome candidate 1	2.001	1.001	0.000	0.000

Table C. Downregulated genes in scalp psoriasis: lesional vs. non-lesional.

	Probe	Symbol	Description	FCH-Scalp-LS-NL	log2FCH-Scalp-LS-NL	p-Scalp-LS-NL	FDR-Scalp-LS-NL
1	220615_s_at	MLSTD1	male sterility domain containing 1	0,052	-4,258	0,001	0,000
2	208964_s_at	FADS1	fatty acid desaturase 1	0,059	-4,079	0,000	0,000
3	202218_s_at	FADS2	fatty acid desaturase 2	0,067	-3,904	0,000	0,000
4	205404_at	HSD11B1	hydroxysteroid (11-beta) dehydrogenase 1	0,068	-3,882	0,000	0,000
5	210576_at	CYP4F8	cytochrome P450, family 4, subfamily F, polypeptide 8	0,070	-3,843	0,001	0,000
6	214240_at	GAL	galanin	0,071	-3,825	0,002	0,010
7	208963_x_at	FADS1	fatty acid desaturase 1	0,073	-3,782	0,000	0,000
8	204515_at	HSD3B1	hydroxy-delta-5-steroid dehydrogenase, 3 beta- and steroid delta-isomerase 1	0,080	-3,640	0,001	0,000
9	208962_s_at	FADS1	fatty acid desaturase 1	0,081	-3,626	0,000	0,000
10	205380_at	PDZK1	PDZ domain containing 1	0,091	-3,460	0,001	0,000
11	205913_at	PLIN	perilipin	0,103	-3,283	0,001	0,000
12	206465_at	ACSBG1	acyl-CoA synthetase bubblegum family member 1	0,112	-3,163	0,002	0,010
13	220801_s_at	HAO2	hydroxyacid oxidase 2 (long chain)	0,114	-3,127	0,001	0,010
14	204836_at	GLDC	glycine dehydrogenase (decarboxylating)	0,116	-3,112	0,001	0,010
15	210297_s_at	MSMB	microseminoprotein, beta-	0,122	-3,032	0,000	0,000
16	213693_s_at	MUC1	mucin 1, cell surface associated	0,126	-2,988	0,000	0,000
17	219429_at	FA2H	fatty acid 2-hydroxylase	0,132	-2,917	0,001	0,000
18	219140_s_at	RBP4	retinol binding protein 4, plasma	0,140	-2,840	0,000	0,000
19	221561_at	SOAT1	sterol O-acyltransferase (acyl-Coenzyme A: cholesterol acyltransferase) 1	0,147	-2,767	0,002	0,010
20	205030_at	FABP7	fatty acid binding protein 7, brain	0,150	-2,734	0,005	0,020
21	214598_at	CLDN8	claudin 8	0,158	-2,660	0,000	0,000
22	207092_at	LEP	leptin (obesity homolog, mouse)	0,160	-2,641	0,000	0,000
23	210002_at	GATA6	GATA binding protein 6	0,172	-2,540	0,000	0,000
24	204416_x_at	APOC1	apolipoprotein C-I	0,173	-2,528	0,003	0,010
25	221470_s_at	IL1F7	interleukin 1 family, member 7 (zeta)	0,173	-2,528	0,000	0,000
26	205029_s_at	FABP7	fatty acid binding protein 7, brain	0,176	-2,509	0,006	0,020
27	213706_at	GPD1	glycerol-3-phosphate dehydrogenase 1 (soluble)	0,178	-2,490	0,000	0,000
28	203549_s_at	LPL	lipoprotein lipase	0,179	-2,481	0,000	0,000
29	203548_s_at	LPL	lipoprotein lipase	0,182	-2,460	0,002	0,010
30	205883_at	ZBTB16	zinc finger and BTB domain containing 16	0,185	-2,438	0,000	0,000
31	204712_at	WIF1	WNT inhibitory factor 1	0,187	-2,419	0,001	0,010
32	209522_s_at	CRAT	carnitine acetyltransferase	0,193	-2,372	0,001	0,000
33	220065_at	TNMD	tenomodulin	0,196	-2,349	0,000	0,000
34	202982_s_at	ACOT2	acyl-CoA thioesterase 2	0,199	-2,331	0,001	0,000
35	207430_s_at	MSMB	microseminoprotein, beta-	0,204	-2,293	0,000	0,000
36	205364_at	ACOX2	acyl-Coenzyme A oxidase 2, branched chain	0,205	-2,286	0,000	0,000
37	208383_s_at	PCK1	phosphoenolpyruvate carboxykinase 1 (soluble)	0,210	-2,255	0,000	0,000
38	219398_at	CIDEC	cell death-inducing DFFA-like effector c	0,210	-2,253	0,001	0,000
39	221142_s_at	PECR	peroxisomal trans-2-enoyl-CoA reductase	0,217	-2,206	0,005	0,020
40	206243_at	TIMP4	TIMP metallopeptidase inhibitor 4	0,218	-2,197	0,000	0,000
41	207847_s_at	MUC1	mucin 1, cell surface associated	0,221	-2,180	0,001	0,010
42	210096_at	CYP4B1	cytochrome P450, family 4, subfamily B, polypeptide 1	0,223	-2,168	0,001	0,000
43	207955_at	CCL27	chemokine (C-C motif) ligand 27	0,225	-2,155	0,003	0,010
44	204154_at	CDO1	cysteine dioxygenase, type I	0,226	-2,149	0,000	0,000
45	218980_at	FHOD3	formin homology 2 domain containing 3	0,229	-2,125	0,000	0,000
46	206149_at	CHP2	calcineurin B homologous protein 2	0,232	-2,110	0,000	0,000
47	209094_at	DDAH1	dimethylarginine dimethylaminohydrolase 1	0,237	-2,076	0,000	0,000
48	209581_at	HRASLS3	HRAS-like suppressor 3	0,238	-2,074	0,000	0,000
49	204939_s_at	PLN	phospholamban	0,242	-2,046	0,000	0,000
50	213234_at	KIAA1467	KIAA1467	0,243	-2,040	0,000	0,000
51	49452_at	ACACB	acetyl-Coenzyme A carboxylase beta	0,247	-2,015	0,000	0,000
52	205139_s_at	UST	uronyl-2-sulfotransferase	0,249	-2,009	0,000	0,000
53	204607_at	HMGCS2	3-hydroxy-3-methylglutaryl-Coenzyme A synthase 2 (mitochondrial)	0,252	-1,986	0,000	0,000
54	206214_at	PLA2G7	phospholipase A2, group VII (platelet-activating factor acetylhydrolase, plasma)	0,252	-1,991	0,001	0,000
55	205478_at	PPP1R1A	protein phosphatase 1, regulatory (inhibitor) subunit 1A	0,261	-1,939	0,000	0,000
56	205843_x_at	CRAT	carnitine acetyltransferase	0,261	-1,936	0,001	0,010
57	206373_at	ZIC1	Zic family member 1 (odd-paired homolog, Drosophila)	0,263	-1,925	0,001	0,000
58	219689_at	SEMA3G	sema domain, immunoglobulin domain (Ig), short basic domain, secreted, (semaphorin) 3G	0,266	-1,910	0,000	0,000
59	203296_s_at	ATP1A2	ATPase, Na+/K+ transporting, alpha 2 (+) polypeptide	0,267	-1,906	0,000	0,000
60	222071_s_at	SLCO4C1	solute carrier organic anion transporter family, member 4C1	0,268	-1,901	0,008	0,030
61	201625_s_at	INSIG1	insulin induced gene 1	0,272	-1,878	0,003	0,010
62	201626_at	INSIG1	insulin induced gene 1	0,277	-1,850	0,000	0,000
63	215768_at	SOX5	SRY (sex determining region Y)-box 5	0,277	-1,851	0,000	0,000
64	212218_s_at	FASN	fatty acid synthase	0,279	-1,840	0,000	0,000
65	202992_at	C7	complement component 7	0,285	-1,811	0,000	0,000
66	209343_at	EFHD1	EF-hand domain family, member D1	0,287	-1,801	0,001	0,010
67	209866_s_at	LPHN3	latrophilin 3	0,287	-1,800	0,008	0,030
68	209867_s_at	LPHN3	latrophilin 3	0,289	-1,791	0,008	0,030
69	203980_at	FABP4	fatty acid binding protein 4, adipocyte	0,290	-1,784	0,000	0,000
70	205381_at	LRRC17	leucine rich repeat containing 17	0,293	-1,769	0,000	0,000
71	205518_s_at	CMAH	cytidine monophosphate-N-acetylneuraminic acid hydroxylase (CMP-N-acetylneuraminate monooxygenase)	0,293	-1,773	0,002	0,010

	Probe	Symbol	Description	FCH-Scalp-LS-NL	log2FCH-Scalp-LS-NL	p-Scalp-LS-NL	FDR-Scalp-LS-NL
72	203766_s_at	LMOD1	leiomodin 1 (smooth muscle)	0,297	-1,752	0,001	0,000
73	214027_x_at	DES	desmin	0,297	-1,751	0,000	0,000
74	215516_at	LAMB4	laminin, beta 4	0,298	-1,745	0,001	0,000
75	213050_at	COBL	cordon-bleu homolog (mouse)	0,299	-1,740	0,000	0,000
76	217897_at	FXYD6	FXYD domain containing ion transport regulator 6	0,299	-1,744	0,005	0,020
77	206466_at	ACSBG1	acyl-CoA synthetase bubblegum family member 1	0,300	-1,736	0,002	0,010
78	209613_s_at	ADH1B	alcohol dehydrogenase IB (class I), beta polypeptide	0,300	-1,739	0,004	0,020
79	218087_s_at	SORBS1	sorbin and SH3 domain containing 1	0,300	-1,737	0,000	0,000
80	219195_at	PPARGC1A	peroxisome proliferator-activated receptor gamma, coactivator 1 alpha	0,302	-1,727	0,000	0,000
81	43427_at	ACACB	acetyl-Coenzyme A carboxylase beta	0,303	-1,723	0,000	0,000
82	215695_s_at	GYG2	glycogenin 2	0,306	-1,709	0,003	0,010
83	205768_s_at	SLC27A2	solute carrier family 27 (fatty acid transporter), member 2	0,308	-1,700	0,009	0,040
84	203895_at	PLCB4	phospholipase C, beta 4	0,314	-1,673	0,002	0,010
85	205549_at	PCP4	Purkinje cell protein 4	0,314	-1,670	0,001	0,000
86	213371_at	LDB3	LIM domain binding 3	0,314	-1,673	0,000	0,000
87	212279_at	TMEM97	transmembrane protein 97	0,315	-1,667	0,006	0,030
88	219895_at	FAM70A	family with sequence similarity 70, member A	0,315	-1,665	0,000	0,000
89	207302_at	SGCG	sarcoglycan, gamma (35kDa dystrophin-associated glycoprotein)	0,316	-1,664	0,003	0,010
90	209242_at	PEG3	paternally expressed 3	0,316	-1,662	0,000	0,000
91	221950_at	EMX2	empty spiracles homeobox 2	0,317	-1,657	0,000	0,000
92	205259_at	NR3C2	nuclear receptor subfamily 3, group C, member 2	0,322	-1,636	0,000	0,000
93	206170_at	ADRB2	adrenergic, beta-2-, receptor, surface	0,324	-1,627	0,001	0,010
94	209459_s_at	ABAT	4-aminobutyrate aminotransferase	0,327	-1,613	0,000	0,000
95	32836_at	AGPAT1	1-acylglycerol-3-phosphate O-acyltransferase 1 (lysophosphatidic acid acyltransferase, alpha)	0,329	-1,603	0,001	0,000
96	213413_at	STON1	stonin 1	0,330	-1,599	0,000	0,000
97	204363_at	F3	coagulation factor III (thromboplastin, tissue factor)	0,333	-1,585	0,000	0,000
98	209894_at	LEPR	leptin receptor	0,335	-1,577	0,001	0,000
99	215039_at	LOC339524	hypothetical protein LOC339524	0,335	-1,577	0,000	0,000
100	203824_at	TSPAN8	tetraspanin 8	0,336	-1,572	0,002	0,010
101	201627_s_at	INSIG1	insulin induced gene 1	0,338	-1,565	0,005	0,020
102	212157_at	SDC2	syndecan 2	0,338	-1,564	0,000	0,000
103	219407_s_at	LAMC3	laminin, gamma 3	0,339	-1,561	0,000	0,000
104	205044_at	GABRP	gamma-aminobutyric acid (GABA) A receptor, pi	0,340	-1,558	0,001	0,000
105	221530_s_at	BHLHB3	basic helix-loop-helix domain containing, class B, 3	0,340	-1,558	0,001	0,010
106	205325_at	PHYHIP	phytanoyl-CoA 2-hydroxylase interacting protein	0,342	-1,548	0,000	0,000
107	204940_at	PLN	phospholamban	0,345	-1,535	0,001	0,000
108	212913_at	C6orf26	chromosome 6 open reading frame 26	0,345	-1,537	0,003	0,010
109	219115_s_at	IL20RA	interleukin 20 receptor, alpha	0,346	-1,531	0,001	0,000
110	206799_at	SCGB1D2	secretoglobin, family 1D, member 2	0,347	-1,528	0,001	0,010
111	205160_at	PEX11A	peroxisomal biogenesis factor 11A	0,348	-1,523	0,000	0,000
112	204894_s_at	AOC3	amine oxidase, copper containing 3 (vascular adhesion protein 1)	0,349	-1,520	0,000	0,000
113	202975_s_at	RHOBTB3	Rho-related BTB domain containing 3	0,350	-1,513	0,000	0,000
114	212282_at	TMEM97	transmembrane protein 97	0,351	-1,511	0,002	0,010
115	210964_s_at	GYG2	glycogenin 2	0,352	-1,508	0,002	0,010
116	215322_at	LONRF1	LON peptidase N-terminal domain and ring finger 1	0,355	-1,494	0,001	0,010
117	208510_s_at	PPARG	peroxisome proliferator-activated receptor gamma	0,356	-1,490	0,000	0,000
118	215271_at	TNN	tenascin N	0,356	-1,488	0,005	0,020
119	202363_at	SPOCK1	sparc/osteonectin, cwcv and kazal-like domains proteoglycan (testican) 1	0,357	-1,485	0,000	0,000
120	221974_at	PWCR1	Prader-Willi syndrome chromosome region 1	0,357	-1,486	0,000	0,000
121	209612_s_at	ADH1B	alcohol dehydrogenase IB (class I), beta polypeptide	0,361	-1,470	0,005	0,020
122	201596_x_at	KRT18	keratin 18	0,363	-1,464	0,008	0,030
123	204422_s_at	FGF2	fibroblast growth factor 2 (basic)	0,364	-1,459	0,002	0,010
124	209663_s_at	ITGA7	integrin, alpha 7	0,365	-1,452	0,000	0,000
125	213388_at	LOC727942	similar to phosphodiesterase 4D interacting protein isoform 2	0,365	-1,452	0,000	0,000
126	205908_s_at	OMD	osteomodulin	0,366	-1,451	0,008	0,030
127	214293_at	SEPT11	septin 11	0,368	-1,441	0,000	0,000
128	206509_at	PIP	prolactin-induced protein	0,372	-1,428	0,008	0,030
129	204557_s_at	DZIP1	DAZ interacting protein 1	0,373	-1,423	0,000	0,000
130	218532_s_at	FAM134B	family with sequence similarity 134, member B	0,373	-1,425	0,000	0,000
131	201496_x_at	MYH11	myosin, heavy chain 11, smooth muscle	0,375	-1,417	0,000	0,000
132	215358_x_at	ZNF37B	zinc finger protein 37B	0,376	-1,412	0,001	0,000
133	37117_at	ARHGAP8	Rho GTPase activating protein 8	0,376	-1,410	0,000	0,000
134	214823_at	ZNF204	zinc finger protein 204	0,377	-1,408	0,000	0,000
135	209616_s_at	CES1	carboxylesterase 1 (monocyte/macrophage serine esterase 1)	0,378	-1,402	0,003	0,010
136	212281_s_at	TMEM97	transmembrane protein 97	0,378	-1,404	0,002	0,010
137	214433_s_at	SELENBP1	selenium binding protein 1	0,378	-1,404	0,001	0,000
139	208788_at	ELOVL5	ELOVL family member 5, elongation of long chain fatty acids (FEN1/Elo2, SUR4/Elo3-like, yeast)	0,379	-1,401	0,002	0,010
140	213369_at	PCDH21	protocadherin 21	0,379	-1,399	0,007	0,030
141	221552_at	ABHD6	abhydrolase domain containing 6	0,379	-1,400	0,000	0,000
143	202409_at	IGF2	insulin-like growth factor 2 (somatomedin A)	0,380	-1,395	0,003	0,020
144	218974_at	SOBP	sine oculis binding protein homolog (Drosophila)	0,380	-1,396	0,000	0,000
145	202222_s_at	DES	desmin	0,381	-1,391	0,001	0,000
146	202555_s_at	MYLK	myosin, light chain kinase	0,382	-1,389	0,000	0,000
147	215129_at	PIK3C2G	phosphoinositide-3-kinase, class 2, gamma polypeptide	0,382	-1,388	0,000	0,000
148	203680_at	PRKAR2B	protein kinase, cAMP-dependent, regulatory, type II, beta	0,383	-1,383	0,001	0,000

	Probe	Symbol	Description	FCH-Scalp-LS-NL	log2FCH-Scalp-LS-NL	p-Scalp-LS-NL	FDR-Scalp-LS-NL
149	204589_at	NUAK1	NUAK family, SNF1-like kinase, 1	0,383	-1,386	0,000	0,000
150	205498_at	GHR	growth hormone receptor	0,383	-1,385	0,001	0,000
151	205541_s_at	GSPT2	G1 to S phase transition 2	0,383	-1,383	0,001	0,000
152	209292_at	ID4	inhibitor of DNA binding 4, dominant negative helix-loop-helix protein	0,384	-1,382	0,000	0,000
153	209614_at	ADH1B	alcohol dehydrogenase IB (class I), beta polypeptide	0,384	-1,382	0,003	0,010
154	219132_at	PELI2	pellino homolog 2 (Drosophila)	0,384	-1,381	0,000	0,000
155	219263_at	RNF128	ring finger protein 128	0,385	-1,379	0,002	0,010
156	203151_at	MAP1A	microtubule-associated protein 1A	0,386	-1,374	0,003	0,020
157	214761_at	ZNF423	zinc finger protein 423	0,386	-1,374	0,002	0,010
158	204591_at	CHL1	cell adhesion molecule with homology to L1CAM (close homolog of L1)	0,387	-1,371	0,000	0,000
159	214297_at	CSPG4	chondroitin sulfate proteoglycan 4	0,388	-1,365	0,002	0,010
160	201951_at	ALCAM	activated leukocyte cell adhesion molecule	0,390	-1,359	0,000	0,000
162	201952_at	ALCAM	activated leukocyte cell adhesion molecule	0,391	-1,353	0,000	0,000
163	204032_at	BCAR3	breast cancer anti-estrogen resistance 3	0,391	-1,353	0,000	0,000
164	207071_s_at	ACO1	aconitase 1, soluble	0,392	-1,351	0,001	0,000
165	209602_s_at	GATA3	GATA binding protein 3	0,393	-1,348	0,000	0,000
166	219525_at	SLC47A1	solute carrier family 47, member 1	0,394	-1,343	0,002	0,010
167	221234_s_at	BACH2	BTB and CNC homology 1, basic leucine zipper transcription factor 2	0,394	-1,344	0,000	0,000
168	208016_s_at	AGTR1	angiotensin II receptor, type 1	0,395	-1,341	0,004	0,020
169	202908_at	WFS1	Wolfram syndrome 1 (wolframin)	0,396	-1,335	0,000	0,000
170	204519_s_at	PLLP	plasma membrane proteolipid (plasmolipin)	0,397	-1,332	0,003	0,010
171	204964_s_at	SSPN	sarcospan (Kras oncogene-associated gene)	0,398	-1,329	0,000	0,000
172	219440_at	RAI2	retinoic acid induced 2	0,398	-1,330	0,000	0,000
173	204997_at	GPD1	glycerol-3-phosphate dehydrogenase 1 (soluble)	0,399	-1,327	0,003	0,020
174	213800_at	CFH	complement factor H	0,399	-1,326	0,000	0,000
175	218312_s_at	ZSCAN18	zinc finger and SCAN domain containing 18	0,400	-1,323	0,002	0,010
176	204793_at	GPRASP1	G protein-coupled receptor associated sorting protein 1	0,401	-1,317	0,000	0,000
177	220197_at	ATP6V0A4	ATPase, H+ transporting, lysosomal V0 subunit a4	0,403	-1,310	0,010	0,040
178	221747_at	TNS1	tensin 1	0,404	-1,309	0,000	0,000
179	40837_at	TLE2	transducin-like enhancer of split 2 (E(sp1) homolog, Drosophila)	0,404	-1,307	0,002	0,010
180	213900_at	C9orf61	chromosome 9 open reading frame 61	0,405	-1,303	0,001	0,010
181	214719_at	SLC46A3	solute carrier family 46, member 3	0,405	-1,304	0,001	0,000
182	202976_s_at	RHOBTB3	Rho-related BTB domain containing 3	0,406	-1,300	0,000	0,000
183	206201_s_at	MEOX2	mesenchyme homeobox 2	0,406	-1,302	0,000	0,000
184	205374_at	SLN	sarcolipin	0,407	-1,297	0,005	0,020
185	206167_s_at	ARHGAP6	Rho GTPase activating protein 6	0,407	-1,295	0,002	0,010
186	215506_s_at	DIRAS3	DIRAS family, GTP-binding RAS-like 3	0,407	-1,296	0,005	0,020
187	219304_s_at	PDGFD	platelet derived growth factor D	0,409	-1,290	0,000	0,000
188	206101_at	ECM2	extracellular matrix protein 2, female organ and adipocyte specific	0,410	-1,286	0,002	0,010
189	201497_x_at	MYH11	myosin, heavy chain 11, smooth muscle	0,411	-1,283	0,000	0,000
190	204294_at	AMT	aminomethyltransferase	0,411	-1,282	0,000	0,000
191	204736_s_at	CSPG4	chondroitin sulfate proteoglycan 4	0,411	-1,284	0,007	0,030
192	202437_s_at	CYP1B1	cytochrome P450, family 1, subfamily B, polypeptide 1	0,412	-1,278	0,002	0,010
193	204442_x_at	LTBP4	latent transforming growth factor beta binding protein 4	0,412	-1,279	0,003	0,010
194	213217_at	ADCY2	adenylate cyclase 2 (brain)	0,412	-1,279	0,003	0,010
195	220675_s_at	PNPLA3	patatin-like phospholipase domain containing 3	0,412	-1,280	0,006	0,030
196	45288_at	ABHD6	abhydrolase domain containing 6	0,412	-1,281	0,000	0,000
197	218676_s_at	PCTP	phosphatidylcholine transfer protein	0,413	-1,277	0,004	0,020
198	210517_s_at	AKAP12	A kinase (PRKA) anchor protein (gravin) 12	0,415	-1,269	0,001	0,000
199	219854_at	ZNF14	zinc finger protein 14	0,415	-1,267	0,000	0,000
200	215028_at	SEMA6A	sema domain, transmembrane domain (TM), and cytoplasmic domain, (semaphorin) 6A	0,416	-1,265	0,002	0,010
201	221928_at	ACACB	acetyl-Coenzyme A carboxylase beta	0,417	-1,263	0,000	0,000
202	202016_at	MEST	mesoderm specific transcript homolog (mouse)	0,418	-1,257	0,009	0,040
203	206028_s_at	MERTK	c-mer proto-oncogene tyrosine kinase	0,418	-1,257	0,000	0,000
204	217025_s_at	DBN1	drebrin 1	0,418	-1,259	0,006	0,020
205	203951_at	CNN1	calponin 1, basic, smooth muscle	0,419	-1,254	0,001	0,000
206	205141_at	ANG	angiogenin, ribonuclease, RNase A family, 5	0,419	-1,255	0,000	0,000
207	205856_at	SLC14A1	solute carrier family 14 (urea transporter), member 1 (Kidd blood group)	0,419	-1,255	0,007	0,030
208	214434_at	HSPA12A	heat shock 70kDa protein 12A	0,419	-1,254	0,001	0,000
209	220432_s_at	CYP39A1	cytochrome P450, family 39, subfamily A, polypeptide 1	0,419	-1,255	0,000	0,000
210	218510_x_at	FAM134B	family with sequence similarity 134, member B	0,420	-1,252	0,001	0,000
211	206656_s_at	C20orf3	chromosome 20 open reading frame 3	0,421	-1,247	0,001	0,000
212	207034_s_at	GLI2	GLI-Kruppel family member GLI2	0,421	-1,247	0,000	0,000
213	213106_at	ATP8A1	ATPase, aminophospholipid transporter (APLT), Class I, type 8A, member 1	0,422	-1,244	0,000	0,000
214	213935_at	ABHD5	abhydrolase domain containing 5	0,422	-1,244	0,002	0,010
215	204963_at	SSPN	sarcospan (Kras oncogene-associated gene)	0,423	-1,241	0,000	0,000
216	212730_at	DMN	desmuslin	0,424	-1,236	0,001	0,010
217	214375_at	PPFIBP1	PTPRF interacting protein, binding protein 1 (liprin beta 1)	0,424	-1,237	0,003	0,010
218	219778_at	ZFPM2	zinc finger protein, multitype 2	0,424	-1,238	0,005	0,020
219	205440_s_at	NPY1R	neuropeptide Y receptor Y1	0,425	-1,234	0,000	0,000
220	205529_s_at	RUNX1T1	runt-related transcription factor 1; translocated to, 1 (cyclin D-related)	0,425	-1,235	0,000	0,000
221	209543_s_at	CD34	CD34 molecule	0,425	-1,233	0,011	0,040
222	213071_at	DPT	dermatopontin	0,425	-1,233	0,000	0,000
223	201058_s_at	MYL9	myosin, light chain 9, regulatory	0,426	-1,231	0,000	0,000
224	210078_s_at	KCNAB1	potassium voltage-gated channel, shaker-related subfamily, beta member 1	0,426	-1,230	0,005	0,020

	Probe	Symbol	Description	FCH-Scalp-LS-NL	log2FCH-Scalp-LS-NL	p-Scalp-LS-NL	FDR-Scalp-LS-NL
225	202274_at	ACTG2	actin, gamma 2, smooth muscle, enteric	0,428	-1,226	0,001	0,000
226	211105_s_at	NFATC1	nuclear factor of activated T-cells, cytoplasmic, calcineurin-dependent 1	0,428	-1,223	0,002	0,010
227	201124_at	ITGB5	integrin, beta 5	0,429	-1,222	0,000	0,000
228	201839_s_at	TACSTD1	tumor-associated calcium signal transducer 1	0,429	-1,222	0,002	0,010
229	202436_s_at	CYP1B1	cytochrome P450, family 1, subfamily B, polypeptide 1	0,429	-1,221	0,006	0,020
230	221589_s_at	ALDH6A1	aldehyde dehydrogenase 6 family, member A1	0,429	-1,219	0,000	0,000
231	207961_x_at	MYH11	myosin, heavy chain 11, smooth muscle	0,430	-1,216	0,000	0,000
232	209072_at	MBP	myelin basic protein	0,430	-1,218	0,004	0,020
233	201307_at	SEPT11	septin 11	0,431	-1,213	0,000	0,000
234	213317_at	CLIC5	chloride intracellular channel 5	0,431	-1,215	0,003	0,010
235	213568_at	OSR2	odd-skipped related 2 (Drosophila)	0,431	-1,214	0,000	0,000
236	202321_at	GGPS1	geranylgeranyl diphosphate synthase 1	0,432	-1,211	0,005	0,020
237	212503_s_at	DIP2C	DIP2 disco-interacting protein 2 homolog C (Drosophila)	0,432	-1,211	0,004	0,020
238	213397_x_at	ANG	angiogenin, ribonuclease, RNase A family, 5	0,432	-1,211	0,000	0,000
239	212850_s_at	LRP4	low density lipoprotein receptor-related protein 4	0,433	-1,207	0,002	0,010
240	212099_at	RHOB	ras homolog gene family, member B	0,435	-1,201	0,000	0,000
241	213925_at	C1orf95	chromosome 1 open reading frame 95	0,435	-1,201	0,000	0,000
242	209656_s_at	TMEM47	transmembrane protein 47	0,436	-1,197	0,000	0,000
243	216331_at	ITGA7	integrin, alpha 7	0,436	-1,197	0,000	0,000
244	221590_s_at	ALDH6A1	aldehyde dehydrogenase 6 family, member A1	0,436	-1,198	0,010	0,040
246	218820_at	C14orf132	chromosome 14 open reading frame 132	0,438	-1,191	0,000	0,000
247	203903_s_at	HEPH	hephaestin	0,439	-1,189	0,000	0,000
248	205384_at	FXYD1	FXYD domain containing ion transport regulator 1 (phospholemman)	0,439	-1,187	0,001	0,010
249	212741_at	MAOA	monoamine oxidase A	0,439	-1,186	0,000	0,000
250	218517_at	PHF17	PHD finger protein 17	0,439	-1,186	0,000	0,000
251	202746_at	ITM2A	integral membrane protein 2A	0,440	-1,184	0,000	0,000
252	213122_at	TSPYL5	TSPY-like 5	0,440	-1,184	0,000	0,000
253	202242_at	TSPAN7	tetraspanin 7	0,441	-1,181	0,002	0,010
254	201540_at	FHL1	four and a half LIM domains 1	0,442	-1,179	0,000	0,000
255	209291_at	ID4	inhibitor of DNA binding 4, dominant negative helix-loop-helix protein	0,442	-1,178	0,000	0,000
257	218309_at	CAMK2N1	calcium/calmodulin-dependent protein kinase II inhibitor 1	0,442	-1,179	0,000	0,000
258	209122_at	ADFP	adipose differentiation-related protein	0,443	-1,174	0,001	0,010
259	209074_s_at	FAM107A	family with sequence similarity 107, member A	0,444	-1,172	0,001	0,010
260	219737_s_at	PCDH9	protocadherin 9	0,444	-1,171	0,000	0,000
261	217901_at	DSG2	desmoglein 2	0,445	-1,167	0,001	0,000
262	204688_at	SGCE	sarcoglycan, epsilon	0,446	-1,164	0,000	0,000
265	205428_s_at	CALB2	calbindin 2, 29kDa (calretinin)	0,448	-1,157	0,001	0,000
266	215235_at	SPTAN1	spectrin, alpha, non-erythrocytic 1 (alpha-fodrin)	0,448	-1,159	0,001	0,010
267	204083_s_at	TPM2	tropomyosin 2 (beta)	0,449	-1,155	0,000	0,000
268	217047_s_at	FAM13A1	family with sequence similarity 13, member A1	0,449	-1,154	0,000	0,000
269	205277_at	PRDM2	PR domain containing 2, with ZNF domain	0,450	-1,153	0,000	0,000
270	202052_s_at	RAI14	retinoic acid induced 14	0,451	-1,149	0,000	0,000
271	204288_s_at	SORBS2	sorbin and SH3 domain containing 2	0,451	-1,150	0,000	0,000
272	220559_at	EN1	engrailed homeobox 1	0,451	-1,150	0,001	0,010
273	215535_s_at	AGPAT1	1-acylglycerol-3-phosphate O-acyltransferase 1 (lysophosphatidic acid acyltransferase, alpha)	0,452	-1,145	0,001	0,000
274	201525_at	APOD	apolipoprotein D	0,453	-1,142	0,003	0,010
275	202973_x_at	FAM13A1	family with sequence similarity 13, member A1	0,453	-1,144	0,000	0,000
276	214464_at	CDC42BPA	CDC42 binding protein kinase alpha (DMPK-like)	0,453	-1,142	0,007	0,030
277	209815_at	PTCH1	patched homolog 1 (Drosophila)	0,455	-1,137	0,000	0,000
278	212158_at	SDC2	syndecan 2	0,455	-1,135	0,000	0,000
279	218625_at	NRN1	neuritin 1	0,455	-1,136	0,003	0,010
280	205407_at	RECK	reversion-inducing-cysteine-rich protein with kazal motifs	0,456	-1,133	0,003	0,010
281	214890_s_at	DKFZP564J102	DKFZP564J102 protein	0,456	-1,134	0,010	0,040
282	202502_at	ACADM	acyl-Coenzyme A dehydrogenase, C-4 to C-12 straight chain	0,457	-1,128	0,004	0,020
283	212276_at	LPIN1	lipin 1	0,457	-1,130	0,001	0,000
284	204734_at	KRT15	keratin 15	0,458	-1,126	0,006	0,030
285	209283_at	CRYAB	crystallin, alpha B	0,459	-1,123	0,001	0,000
286	209869_at	ADRA2A	adrenergic, alpha-2A-, receptor	0,459	-1,123	0,001	0,010
287	217506_at	LOC400642	hypothetical gene supported by BC041875; BX648984	0,459	-1,124	0,001	0,010
288	218934_s_at	HSPB7	heat shock 27kDa protein family, member 7 (cardiovascular)	0,459	-1,123	0,006	0,030
289	202003_s_at	ACAA2	acetyl-Coenzyme A acyltransferase 2 (mitochondrial 3-oxoacyl-Coenzyme A thiolase)	0,460	-1,121	0,005	0,020
290	205609_at	ANGPT1	angiopoietin 1	0,460	-1,119	0,006	0,030
291	212494_at	TENC1	tensin like C1 domain containing phosphatase (tensin 2)	0,460	-1,119	0,001	0,000
292	219765_at	ZNF329	zinc finger protein 329	0,460	-1,119	0,000	0,000
293	203382_s_at	APOE	apolipoprotein E	0,461	-1,118	0,006	0,020
294	205528_s_at	RUNX1T1	runt-related transcription factor 1; translocated to, 1 (cyclin D-related)	0,461	-1,118	0,000	0,000
295	205730_s_at	ABLIM3	actin binding LIM protein family, member 3	0,461	-1,116	0,000	0,000
296	203920_at	NR1H3	nuclear receptor subfamily 1, group H, member 3	0,462	-1,113	0,000	0,000
297	214724_at	DIXDC1	DIX domain containing 1	0,462	-1,114	0,002	0,010
298	203872_at	ACTA1	actin, alpha 1, skeletal muscle	0,463	-1,112	0,002	0,010
299	205932_s_at	MSX1	msh homeobox 1	0,463	-1,110	0,003	0,020
300	207417_s_at	ZNF177	zinc finger protein 177	0,463	-1,112	0,000	0,000
301	213348_at	CDKN1C	cyclin-dependent kinase inhibitor 1C (p57, Kip2)	0,463	-1,112	0,000	0,000
302	205158_at	RNASE4	ribonuclease, RNase A family, 4	0,464	-1,107	0,000	0,000
303	213228_at	PDE8B	phosphodiesterase 8B	0,464	-1,109	0,003	0,010

	Probe	Symbol	Description	FCH-Scalp-LS-NL	log2FCH-Scalp-LS-NL	p-Scalp-LS-NL	FDR-Scalp-LS-NL
304	212776_s_at	OBSL1	obscurin-like 1	0,465	-1,104	0,007	0,030
305	205352_at	SERPINI1	serpin peptidase inhibitor, clade I (neuroserpin), member 1	0,466	-1,103	0,000	0,000
306	203685_at	BCL2	B-cell CLL/lymphoma 2	0,467	-1,097	0,001	0,000
307	205857_at	SLC18A2	solute carrier family 18 (vesicular monoamine), member 2	0,467	-1,098	0,007	0,030
308	218966_at	MYO5C	myosin VC	0,467	-1,100	0,001	0,010
309	202668_at	EFNB2	ephrin-B2	0,468	-1,095	0,000	0,000
310	212651_at	RHOBTB1	Rho-related BTB domain containing 1	0,468	-1,095	0,001	0,010
311	215139_at	ARHGEF10	Rho guanine nucleotide exchange factor (GEF) 10	0,468	-1,096	0,001	0,000
312	221675_s_at	CHPT1	choline phosphotransferase 1	0,468	-1,095	0,000	0,000
313	210299_s_at	FHL1	four and a half LIM domains 1	0,469	-1,091	0,003	0,010
314	212980_at	USP34	ubiquitin specific peptidase 34	0,469	-1,092	0,007	0,030
315	221748_s_at	TNS1	tensin 1	0,469	-1,091	0,000	0,000
316	214981_at	POSTN	periostin, osteoblast specific factor	0,470	-1,089	0,010	0,040
317	219682_s_at	TBX3	T-box 3 (ulnar mammary syndrome)	0,470	-1,089	0,007	0,030
318	202972_s_at	FAM13A1	family with sequence similarity 13, member A1	0,472	-1,084	0,001	0,000
320	213093_at	PRKCA	protein kinase C, alpha	0,473	-1,079	0,001	0,000
321	209655_s_at	TMEM47	transmembrane protein 47	0,474	-1,076	0,001	0,000
322	212793_at	DAAM2	dishevelled associated activator of morphogenesis 2	0,474	-1,077	0,003	0,020
323	221527_s_at	PARD3	par-3 partitioning defective 3 homolog (C. elegans)	0,474	-1,078	0,000	0,000
324	206059_at	ZNF91	zinc finger protein 91	0,475	-1,075	0,004	0,020
325	203335_at	PHYH	phytanoyl-CoA 2-hydroxylase	0,476	-1,071	0,000	0,000
326	208216_at	DLX4	distal-less homeobox 4	0,476	-1,072	0,000	0,000
327	214920_at	THSD7A	thrombospondin, type I, domain containing 7A	0,476	-1,072	0,006	0,030
328	221646_s_at	ZDHHC11	zinc finger, DHHC-type containing 11	0,476	-1,070	0,007	0,030
329	206378_at	SCGB2A2	secretoglobin, family 2A, member 2	0,477	-1,068	0,001	0,000
330	220428_at	CD207	CD207 molecule, langerin	0,477	-1,068	0,001	0,000
331	222116_s_at	TBC1D16	TBC1 domain family, member 16	0,477	-1,067	0,000	0,000
332	202328_s_at	PKD1	polycystic kidney disease 1 (autosomal dominant)	0,478	-1,063	0,002	0,010
333	208760_at	UBE2I	ubiquitin-conjugating enzyme E2I (UBC9 homolog, yeast)	0,478	-1,066	0,000	0,000
334	214451_at	TFAP2B	transcription factor AP-2 beta (activating enhancer binding protein 2 beta)	0,478	-1,064	0,000	0,000
335	221584_s_at	KCNMA1	potassium large conductance calcium-activated channel, subfamily M, alpha member 1	0,478	-1,066	0,004	0,020
336	204984_at	GPC4	glypican 4	0,479	-1,062	0,000	0,000
337	206898_at	CDH19	cadherin 19, type 2	0,480	-1,059	0,004	0,020
338	208498_s_at	AMY1A	amylase, alpha 1A (salivary)	0,481	-1,055	0,000	0,000
339	213068_at	DPT	dermatopontin	0,481	-1,056	0,001	0,000
340	209603_at	GATA3	GATA binding protein 3	0,482	-1,054	0,002	0,010
341	218872_at	TESC	tescalcin	0,482	-1,054	0,000	0,000
342	212554_at	CAP2	CAP, adenylate cyclase-associated protein, 2 (yeast)	0,483	-1,051	0,001	0,010
343	220076_at	ANKH	ankylosis, progressive homolog (mouse)	0,483	-1,049	0,001	0,000
344	204160_s_at	ENPP4	ectonucleotide pyrophosphatase/phosphodiesterase 4 (putative function)	0,484	-1,048	0,011	0,040
345	209220_at	GPC3	glypican 3	0,484	-1,048	0,001	0,000
346	209737_at	MAGI2	membrane associated guanylate kinase, WW and PDZ domain containing 2	0,484	-1,047	0,001	0,000
347	202512_s_at	ATG5	ATG5 autophagy related 5 homolog (S. cerevisiae)	0,485	-1,043	0,003	0,020
348	205525_at	CALD1	caldesmon 1	0,485	-1,044	0,002	0,010
349	218692_at	GOLSYN	Golgi-localized protein	0,485	-1,044	0,000	0,000
350	218718_at	PDGFC	platelet derived growth factor C	0,486	-1,041	0,000	0,000
351	45297_at	EHD2	EH-domain containing 2	0,486	-1,040	0,004	0,020
352	204686_at	IRS1	insulin receptor substrate 1	0,487	-1,037	0,012	0,040
353	205923_at	RELN	reelin	0,487	-1,037	0,002	0,010
354	209210_s_at	PLEKHC1	pleckstrin homology domain containing, family C (with FERM domain) member 1	0,487	-1,039	0,000	0,000
355	219478_at	WFDC1	WAP four-disulfide core domain 1	0,488	-1,035	0,001	0,010
356	221276_s_at	SYNC1	syncoilin, intermediate filament 1	0,488	-1,035	0,000	0,000
357	201117_s_at	CPE	carboxypeptidase E	0,489	-1,032	0,002	0,010
358	201787_at	FBLN1	fibulin 1	0,489	-1,033	0,003	0,020
359	209604_s_at	GATA3	GATA binding protein 3	0,490	-1,028	0,000	0,000
360	213059_at	CREB3L1	cAMP responsive element binding protein 3-like 1	0,490	-1,028	0,003	0,010
361	218418_s_at	ANKRD25	ankyrin repeat domain 25	0,490	-1,028	0,000	0,000
362	219615_s_at	KCNK5	potassium channel, subfamily K, member 5	0,490	-1,028	0,002	0,010
363	222362_at	HRBL	HIV-1 Rev binding protein-like	0,490	-1,030	0,000	0,000
364	221796_at	NTRK2	neurotrophic tyrosine kinase, receptor, type 2	0,491	-1,027	0,000	0,000
365	205499_at	SRPX2	sushi-repeat-containing protein, X-linked 2	0,492	-1,024	0,002	0,010
366	205547_s_at	TAGLN	transgelin	0,492	-1,022	0,000	0,000
367	208711_s_at	CCND1	cyclin D1	0,492	-1,022	0,004	0,020
368	213182_x_at	CDKN1C	cyclin-dependent kinase inhibitor 1C (p57, Kip2)	0,492	-1,023	0,000	0,000
369	206726_at	PGDS	prostaglandin D2 synthase, hematopoietic	0,493	-1,019	0,005	0,020
370	204276_at	TK2	thymidine kinase 2, mitochondrial	0,494	-1,017	0,000	0,000
371	212510_at	GPD1L	glycerol-3-phosphate dehydrogenase 1-like	0,494	-1,017	0,000	0,000
372	219511_s_at	SNCAIP	synuclein, alpha interacting protein (synphilin)	0,494	-1,019	0,000	0,000
373	203146_s_at	GABBR1	gamma-aminobutyric acid (GABA) B receptor, 1	0,495	-1,014	0,003	0,010
374	209185_s_at	IRS2	insulin receptor substrate 2	0,495	-1,014	0,003	0,010
375	221123_x_at	ZNF395	zinc finger protein 395	0,495	-1,016	0,009	0,030
376	203705_s_at	FZD7	frizzled homolog 7 (Drosophila)	0,496	-1,011	0,007	0,030
377	220486_x_at	TMEM164	transmembrane protein 164	0,496	-1,013	0,002	0,010
378	204161_s_at	ENPP4	ectonucleotide pyrophosphatase/phosphodiesterase 4 (putative function)	0,497	-1,009	0,001	0,000
379	213587_s_at	ATP6V0E2	ATPase, H+ transporting V0 subunit e2	0,497	-1,009	0,000	0,000

	Probe	Symbol	Description	FCH-Scalp-LS-NL	log2FCH-Scalp-LS-NL	p-Scalp-LS-NL	FDR-Scalp-LS-NL
380	219188_s_at	MACROD1	MACRO domain containing 1	0,497	-1,009	0,005	0,020
381	212715_s_at	MICAL3	microtubule associated monoxygenase, calponin and LIM domain containing 3	0,498	-1,005	0,000	0,000
382	201963_at	ACSL1	acyl-CoA synthetase long-chain family member 1	0,499	-1,003	0,001	0,000
383	210762_s_at	DLC1	deleted in liver cancer 1	0,499	-1,004	0,000	0,000
384	215513_at	HYMAI	hydatidiform mole associated and imprinted	0,499	-1,003	0,011	0,040
385	213905_x_at	BGN	biglycan	0,500	-1,001	0,007	0,030

Table D. Upregulated genes in scalp psoriasis: lesional vs. normal.

	Probe	Symbol	Description	FCH-Scalp-LS-Normal	log2FCH-Scalp-LS-Normal	p-Scalp-LS-Normal	FDR-Scalp-LS-Normal
1	205513_at	TCN1	transcobalamin I (vitamin B12 binding protein, R binder family)	295.756	8.208	0.000	0.000
2	205863_at	S100A12	S100 calcium binding protein A12	212.174	7.729	0.000	0.000
3	202859_x_at	IL8	interleukin 8	125.462	6.971	0.000	0.000
4	207356_at	DEFB4	defensin, beta 4	108.933	6.767	0.000	0.000
5	209189_at	FOS	v-fos FBJ murine osteosarcoma viral oncogene homolog	83.141	6.377	0.000	0.000
6	210663_s_at	KYNU	kynureninase (L-kynurenine hydrolase)	57.892	5.855	0.000	0.000
7	204972_at	OAS2	2'-5'-oligoadenylate synthetase 2, 69/71kDa	43.784	5.452	0.000	0.000
8	220322_at	IL1F9	interleukin 1 family, member 9	40.032	5.323	0.000	0.000
9	216834_at	RGS1	regulator of G-protein signaling 1	38.477	5.266	0.000	0.000
10	217388_s_at	KYNU	kynureninase (L-kynurenine hydrolase)	38.009	5.248	0.000	0.000
11	204470_at	CXCL1	chemokine (C-X-C motif) ligand 1 (melanoma growth stimulating activity, alpha)	33.260	5.056	0.000	0.000
12	206561_s_at	AKR1B10	aldo-keto reductase family 1, member B10 (aldose reductase)	32.201	5.009	0.000	0.000
13	204385_at	KYNU	kynureninase (L-kynurenine hydrolase)	31.471	4.976	0.000	0.000
14	205660_at	OASL	2'-5'-oligoadenylate synthetase-like	29.812	4.898	0.000	0.000
15	220664_at	SPRR2C	small proline-rich protein 2C	25.479	4.671	0.000	0.000
16	206133_at	XAF1	XIAP associated factor-1	22.571	4.496	0.000	0.000
17	204415_at	IFI6	interferon, alpha-inducible protein 6	20.343	4.346	0.000	0.000
18	207602_at	TMPRSS11D	transmembrane protease, serine 11D	20.313	4.344	0.000	0.000
19	205783_at	KLK13	kallikrein-related peptidase 13	16.894	4.078	0.000	0.000
20	211906_s_at	SERPINB4	serpin peptidase inhibitor, clade B (ovalbumin), member 4	16.836	4.074	0.000	0.000
21	203691_at	PI3	peptidase inhibitor 3, skin-derived (SKALP)	16.264	4.024	0.000	0.000
22	219352_at	HERC6	hect domain and RLD 6	16.042	4.004	0.000	0.000
23	217315_s_at	KLK13	kallikrein-related peptidase 13	15.938	3.994	0.000	0.000
24	203915_at	CXCL9	chemokine (C-X-C motif) ligand 9	15.202	3.926	0.000	0.000
25	218400_at	OAS3	2'-5'-oligoadenylate synthetase 3, 100kDa	14.249	3.833	0.000	0.000
26	209774_x_at	CXCL2	chemokine (C-X-C motif) ligand 2	13.175	3.720	0.000	0.000
27	211362_s_at	SERPINB13	serpin peptidase inhibitor, clade B (ovalbumin), member 13	12.815	3.680	0.000	0.000
28	41469_at	PI3	peptidase inhibitor 3, skin-derived (SKALP)	12.100	3.597	0.000	0.000
29	210797_s_at	OASL	2'-5'-oligoadenylate synthetase-like	11.726	3.552	0.000	0.000
30	219403_s_at	HPSE	heparanase	11.537	3.528	0.000	0.000
31	216258_s_at	SERPINB13	serpin peptidase inhibitor, clade B (ovalbumin), member 13	11.455	3.518	0.000	0.000
32	214059_at	IFI44	interferon-induced protein 44	10.980	3.457	0.000	0.000
33	204533_at	CXCL10	chemokine (C-X-C motif) ligand 10	10.554	3.400	0.000	0.000
34	214453_s_at	IFI44	interferon-induced protein 44	10.414	3.380	0.000	0.000
35	210413_x_at	SERPINB4	serpin peptidase inhibitor, clade B (ovalbumin), member 4	10.219	3.353	0.000	0.000
36	203779_s_at	MPZL2	myelin protein zero-like 2	10.125	3.340	0.000	0.000
37	211506_s_at	IL8	interleukin 8	10.114	3.338	0.000	0.000
38	204580_at	MMP12	matrix metallopeptidase 12 (macrophage elastase)	9.891	3.306	0.000	0.000
39	202086_at	MX1	myxovirus (influenza virus) resistance 1, interferon-inducible protein p78 (mouse)	9.797	3.292	0.000	0.000
40	203699_s_at	DIO2	deiodinase, iodothyronine, type II	9.709	3.279	0.000	0.000
41	210652_s_at	C1orf34	chromosome 1 open reading frame 34	9.457	3.241	0.000	0.000
42	220658_s_at	ARNTL2	aryl hydrocarbon receptor nuclear translocator-like 2	9.399	3.233	0.000	0.000
43	204439_at	IFI44L	interferon-induced protein 44-like	9.392	3.231	0.000	0.000
44	203153_at	IFIT1	interferon-induced protein with tetratricopeptide repeats 1	9.349	3.225	0.000	0.000
45	202626_s_at	LYN	v-yes-1 Yamaguchi sarcoma viral related oncogene homolog	9.058	3.179	0.000	0.000
46	222223_s_at	IL1F5	interleukin 1 family, member 5 (delta)	8.911	3.156	0.000	0.000
47	202411_at	IFI27	interferon, alpha-inducible protein 27	8.730	3.126	0.000	0.000
48	206134_at	ADAMDEC1	ADAM-like, decysin 1	8.584	3.102	0.000	0.000
49	209969_s_at	STAT1	signal transducer and activator of transcription 1, 91kDa	8.526	3.092	0.000	0.000
50	219850_s_at	EHF	ets homologous factor	8.505	3.088	0.000	0.000
51	205483_s_at	ISG15	ISG15 ubiquitin-like modifier	8.385	3.068	0.000	0.000
52	209773_s_at	RRM2	ribonucleotide reductase M2 polypeptide	8.345	3.061	0.000	0.000
53	212268_at	SERPINB1	serpin peptidase inhibitor, clade B (ovalbumin), member 1	8.007	3.001	0.000	0.000
54	216202_s_at	SPTLC2	serine palmitoyltransferase, long chain base subunit 2	7.988	2.998	0.000	0.000
55	208965_s_at	PYHIN1	pyrin and HIN domain family, member 1	7.928	2.987	0.000	0.000
56	219915_s_at	SLC16A10	solute carrier family 16, member 10 (aromatic amino acid transporter)	7.750	2.954	0.000	0.000
57	214007_s_at	TWF1	twinfilin, actin-binding protein, homolog 1 (Drosophila)	7.548	2.916	0.000	0.000
58	203127_s_at	SPTLC2	serine palmitoyltransferase, long chain base subunit 2	7.534	2.913	0.000	0.000
59	209546_s_at	APOL1	apolipoprotein L, 1	7.475	2.902	0.000	0.000
60	221765_at	UGCG	UDP-glucose ceramide glucosyltransferase	7.402	2.888	0.000	0.000
61	211788_s_at	TREX2	three prime repair exonuclease 2	7.240	2.856	0.000	0.000
62	218943_s_at	DDX58	DEAD (Asp-Glu-Ala-Asp) box polypeptide 58	7.238	2.856	0.000	0.000
63	32128_at	CCL18	chemokine (C-C motif) ligand 18 (pulmonary and activation-regulated)	7.072	2.822	0.000	0.000
64	217521_at			7.026	2.813	0.000	0.000
65	213562_s_at	SQLE	squalene epoxidase	7.014	2.810	0.000	0.000
66	200727_s_at	ACTR2	ARP2 actin-related protein 2 homolog (yeast)	6.930	2.793	0.000	0.000
67	210764_s_at	CYR61	cysteine-rich, angiogenic inducer, 61	6.930	2.793	0.000	0.000
68	221698_s_at	CLEC7A	C-type lectin domain family 7, member A	6.753	2.756	0.000	0.000

	Probe	Symbol	Description	FCH-Scalp-LS-Normal	log2FCH-Scalp-LS-Normal	p-Scalp-LS-Normal	FDR-Scalp-LS-Normal
69	209924_at	CCL18	chemokine (C-C motif) ligand 18 (pulmonary and activation-regulated)	6.552	2.712	0.000	0.000
70	205242_at	CXCL13	chemokine (C-X-C motif) ligand 13 (B-cell chemoattractant)	6.471	2.694	0.000	0.000
71	220780_at	PLA2G3	phospholipase A2, group III	6.449	2.689	0.000	0.000
72	213537_at	HLA-DPA1	major histocompatibility complex, class II, DP alpha 1	6.332	2.663	0.000	0.000
73	220187_at	STEAP4	STEAP family member 4	6.300	2.655	0.000	0.000
74	210754_s_at	LYN	v-yes-1 Yamaguchi sarcoma viral related oncogene homolog	6.293	2.654	0.000	0.000
75	204994_at	MX2	myxovirus (influenza virus) resistance 2 (mouse)	6.250	2.644	0.000	0.000
76	201169_s_at	BHLHB2	basic helix-loop-helix domain containing, class B, 2	6.244	2.642	0.000	0.000
77	215150_at	YOD1	YOD1 OTU deubiquinating enzyme 1 homolog (S. cerevisiae)	6.151	2.621	0.000	0.000
78	209719_x_at	SERPINB3	serpin peptidase inhibitor, clade B (ovalbumin), member 3	6.125	2.615	0.000	0.000
79	209720_s_at	SERPINB3	serpin peptidase inhibitor, clade B (ovalbumin), member 3	6.113	2.612	0.000	0.000
80	200730_s_at	PTP4A1	protein tyrosine phosphatase type IVA, member 1	5.940	2.570	0.000	0.000
81	220330_s_at	SAMSN1	SAM domain, SH3 domain and nuclear localization signals 1	5.846	2.548	0.000	0.000
82	203234_at	UPP1	uridine phosphorylase 1	5.836	2.545	0.000	0.000
83	202357_s_at	CFB	complement factor B	5.773	2.529	0.000	0.000
84	201693_s_at	EGR1	early growth response 1	5.715	2.515	0.000	0.000
85	212587_s_at	PTPRC	protein tyrosine phosphatase, receptor type, C	5.647	2.497	0.000	0.000
86	203214_x_at	CDC2	cell division cycle 2, G1 to S and G2 to M	5.631	2.493	0.000	0.000
87	202869_at	OAS1	2',5'-oligoadenylate synthetase 1, 40/46kDa	5.622	2.491	0.000	0.000
88	201291_s_at	TOP2A	topoisomerase (DNA) II alpha 170kDa	5.545	2.471	0.000	0.000
89	202018_s_at	LTF	lactotransferrin	5.516	2.464	0.000	0.000
90	204747_at	IFIT3	interferon-induced protein with tetratricopeptide repeats 3	5.447	2.446	0.000	0.000
91	205729_at	OSMR	oncostatin M receptor	5.441	2.444	0.000	0.000
92	208097_s_at	TXNDC1	thioredoxin domain containing 1	5.420	2.438	0.000	0.000
93	214697_s_at	ROD1	ROD1 regulator of differentiation 1 (S. pombe)	5.419	2.438	0.000	0.000
94	215223_s_at	SOD2	superoxide dismutase 2, mitochondrial	5.414	2.437	0.000	0.000
95	204858_s_at	ECGF1	endothelial cell growth factor 1 (platelet-derived)	5.409	2.435	0.000	0.000
96	203767_s_at	STS	steroid sulfatase (microsomal), isozyme S	5.394	2.431	0.000	0.000
97	201469_s_at	SHC1	SHC (Src homology 2 domain containing) transforming protein 1	5.382	2.428	0.000	0.000
98	213797_at	RSAD2	radical S-adenosyl methionine domain containing 2	5.343	2.418	0.000	0.000
99	211919_s_at	CXCR4	chemokine (C-X-C motif) receptor 4	5.267	2.397	0.000	0.000
100	200796_s_at	MCL1	myeloid cell leukemia sequence 1 (BCL2-related)	5.253	2.393	0.000	0.000
101	214581_x_at	TNFRSF21	tumor necrosis factor receptor superfamily, member 21	5.213	2.382	0.000	0.000
102	219554_at	RHCG	Rh family, C glycoprotein	5.180	2.373	0.000	0.000
103	220800_s_at	TMOD3	tropomodulin 3 (ubiquitous)	5.160	2.367	0.000	0.000
104	211194_s_at	TP63	tumor protein p63	5.141	2.362	0.000	0.000
105	210138_at	RGS20	regulator of G-protein signaling 20	5.138	2.361	0.000	0.000
106	207749_s_at	PPP2R3A	protein phosphatase 2 (formerly 2A), regulatory subunit B'', alpha	4.927	2.301	0.000	0.000
107	210317_s_at	YWHAE	tyrosine 3-monooxygenase/tryptophan 5-monooxygenase activation protein, epsilon polypeptide	4.920	2.299	0.000	0.000
108	209417_s_at	IFI35	interferon-induced protein 35	4.906	2.295	0.000	0.000
109	211195_s_at	TP63	tumor protein p63	4.904	2.294	0.000	0.000
110	205170_at	STAT2	signal transducer and activator of transcription 2, 113kDa	4.901	2.293	0.000	0.000
111	220528_at	VNN3	vanin 3	4.897	2.292	0.000	0.000
112	206643_at	HAL	histidine ammonia-lyase	4.884	2.288	0.000	0.000
113	210148_at	HIPK3	homeodomain interacting protein kinase 3	4.881	2.287	0.000	0.000
114	219684_at	RTP4	receptor (chemosensory) transporter protein 4	4.876	2.286	0.000	0.000
115	216841_s_at	SOD2	superoxide dismutase 2, mitochondrial	4.856	2.280	0.000	0.000
116	205681_at	BCL2A1	BCL2-related protein A1	4.847	2.277	0.000	0.000
117	218810_at	ZC3H12A	zinc finger CCCH-type containing 12A	4.816	2.268	0.000	0.000
118	215891_s_at	GM2A	GM2 ganglioside activator	4.796	2.262	0.000	0.000
119	33646_g_at	GM2A	GM2 ganglioside activator	4.772	2.255	0.000	0.000
120	218960_at	TMPRSS4	transmembrane protease, serine 4	4.754	2.249	0.000	0.000
121	209257_s_at	SMC3	structural maintenance of chromosomes 3	4.673	2.224	0.000	0.000
122	206004_at	TGM3	transglutaminase 3 (E polypeptide, protein-glutamine-gamma-glutamyltransferase)	4.669	2.223	0.000	0.000
123	201971_s_at	ATP6V1A	ATPase, H+ transporting, lysosomal 70kDa, V1 subunit A	4.668	2.223	0.005	0.010
124	218748_s_at	EXOC5	exocyst complex component 5	4.667	2.222	0.000	0.000
125	206211_at	SELE	selectin E (endothelial adhesion molecule 1)	4.665	2.222	0.000	0.000
126	201860_s_at	PLAT	plasminogen activator, tissue	4.642	2.215	0.000	0.000
127	218349_s_at	ZWILCH	Zwilch, kinetochore associated, homolog (Drosophila)	4.607	2.204	0.000	0.000
128	202768_at	FOSB	FBJ murine osteosarcoma viral oncogene homolog B	4.564	2.190	0.007	0.020
129	207381_at	ALOX12B	arachidonate 12-lipoxygenase, 12R type	4.544	2.184	0.000	0.000
130	205890_s_at	UBD	ubiquitin D	4.501	2.170	0.000	0.000
131	206177_s_at	ARG1	arginase, liver	4.487	2.166	0.000	0.000
132	205767_at	EREG	epiregulin	4.477	2.162	0.000	0.000
133	213875_x_at	C6orf62	chromosome 6 open reading frame 62	4.455	2.156	0.000	0.000
134	206421_s_at	SERPINB7	serpin peptidase inhibitor, clade B (ovalbumin), member 7	4.432	2.148	0.000	0.000
135	205488_at	GZMA	granzyme A (granzyme 1, cytotoxic T-lymphocyte-associated serine esterase 3)	4.412	2.141	0.000	0.000
136	206667_s_at	SCAMP1	secretory carrier membrane protein 1	4.391	2.134	0.001	0.000
137	212290_at	SLC7A1	solute carrier family 7 (cationic amino acid transporter, y+ system), member 1	4.382	2.131	0.000	0.000
138	205991_s_at	PRRX1	paired related homeobox 1	4.356	2.123	0.000	0.000
139	220249_at	HYAL4	hyaluronoglucosaminidase 4	4.356	2.123	0.000	0.000
140	208621_s_at	VIL2	villin 2 (ezrin)	4.331	2.115	0.001	0.000

	Probe	Symbol	Description	FCH-Scalp-LS-Normal	log2FCH-Scalp-LS-Normal	p-Scalp-LS-Normal	FDR-Scalp-LS-Normal
141	213548_s_at	CDV3	CDV3 homolog (mouse)	4.322	2.112	0.000	0.000
142	202270_at	GBP1	guanylate binding protein 1, interferon-inducible, 67kDa	4.321	2.111	0.000	0.000
143	215177_s_at	ITGA6	integrin, alpha 6	4.320	2.111	0.000	0.000
144	213572_s_at	SERPINB1	serpin peptidase inhibitor, clade B (ovalbumin), member 1	4.310	2.108	0.000	0.000
145	205842_s_at	JAK2	Janus kinase 2 (a protein tyrosine kinase)	4.306	2.106	0.000	0.000
146	220104_at	ZC3HAV1	zinc finger CCCH-type, antiviral 1	4.305	2.106	0.000	0.000
147	208744_x_at	HSPH1	heat shock 105kDa/110kDa protein 1	4.289	2.101	0.000	0.000
148	214226_at	POL3S	polyserase 3	4.261	2.091	0.000	0.000
149	218384_at	CARHSP1	calcium regulated heat stable protein 1, 24kDa	4.204	2.072	0.000	0.000
150	205724_at	PKP1	plakophilin 1 (ectodermal dysplasia/skin fragility syndrome)	4.179	2.063	0.000	0.000
151	202531_at	IRF1	interferon regulatory factor 1	4.178	2.063	0.000	0.000
152	204995_at	CDK5R1	cyclin-dependent kinase 5, regulatory subunit 1 (p35)	4.167	2.059	0.000	0.000
153	211834_s_at	TP63	tumor protein p63	4.164	2.058	0.000	0.000
154	205401_at	AGPS	alkylglycerone phosphate synthase	4.152	2.054	0.000	0.000
155	206932_at	CH25H	cholesterol 25-hydroxylase	4.149	2.053	0.000	0.000
156	204128_s_at	RFC3	replication factor C (activator 1) 3, 38kDa	4.136	2.048	0.000	0.000
157	201858_s_at	SRGN	serglycin	4.130	2.046	0.000	0.000
158	203764_at	DLG7	discs, large homolog 7 (Drosophila)	4.126	2.045	0.000	0.000
159	209060_x_at	NCOA3	nuclear receptor coactivator 3	4.120	2.042	0.000	0.000
160	201289_at	CYR61	cysteine-rich, angiogenic inducer, 61	4.114	2.041	0.000	0.000
161	215125_s_at	UGT1A6	UDP glucuronosyltransferase 1 family, polypeptide A6	4.110	2.039	0.000	0.000
162	210852_s_at	AASS	aminoadipate-semialdehyde synthase	4.086	2.031	0.000	0.000
163	201295_s_at	WSB1	WD repeat and SOCS box-containing 1	4.061	2.022	0.000	0.000
164	205552_s_at	OAS1	2',5'-oligoadenylate synthetase 1, 40/46kDa	4.035	2.013	0.000	0.000
165	208596_s_at	UGT1A3	UDP glucuronosyltransferase 1 family, polypeptide A3	4.035	2.013	0.000	0.000
166	202988_s_at	RGS1	regulator of G-protein signaling 1	4.030	2.011	0.000	0.000
167	200841_s_at	EPRS	glutamyl-prolyl-tRNA synthetase	3.995	1.998	0.000	0.000
168	201151_s_at	MBNL1	muscleblind-like (Drosophila)	3.991	1.997	0.000	0.000
169	204962_s_at	CENPA	centromere protein A	3.989	1.996	0.000	0.000
170	201890_at	RRM2	ribonucleotide reductase M2 polypeptide	3.981	1.993	0.000	0.000
171	209236_at	SLC23A2	solute carrier family 23 (nucleobase transporters), member 2	3.968	1.989	0.000	0.000
172	209723_at	SERPINB9	serpin peptidase inhibitor, clade B (ovalbumin), member 9	3.968	1.988	0.000	0.000
173	211622_s_at	ARF3	ADP-ribosylation factor 3	3.956	1.984	0.000	0.000
174	219691_at	SAMD9	sterile alpha motif domain containing 9	3.936	1.977	0.000	0.000
175	218454_at	FLJ22662	hypothetical protein FLJ22662	3.924	1.972	0.000	0.000
176	219679_s_at	WAC	WW domain containing adaptor with coiled-coil	3.922	1.972	0.000	0.000
177	212021_s_at	MKI67	antigen identified by monoclonal antibody Ki-67	3.921	1.971	0.000	0.000
178	206429_at	F2RL1	coagulation factor II (thrombin) receptor-like 1	3.916	1.969	0.000	0.000
179	211090_s_at	PRPF4B	PRP4 pre-mRNA processing factor 4 homolog B (yeast)	3.900	1.963	0.000	0.000
180	214490_at	ARSF	arylsulfatase F	3.888	1.959	0.000	0.000
181	201041_s_at	DUSP1	dual specificity phosphatase 1	3.885	1.958	0.000	0.000
182	213988_s_at	SAT1	spermidine/spermine N1-acetyltransferase 1	3.879	1.956	0.000	0.000
183	203560_at	GGH	gamma-glutamyl hydrolase (conjugase, folylpolygammaglutamyl hydrolase)	3.876	1.955	0.000	0.000
184	204057_at	IRF8	interferon regulatory factor 8	3.872	1.953	0.000	0.000
185	219978_s_at	NUSAP1	nucleolar and spindle associated protein 1	3.861	1.949	0.000	0.000
186	201211_s_at	DDX3X	DEAD (Asp-Glu-Ala-Asp) box polypeptide 3, X-linked	3.842	1.942	0.000	0.000
187	201663_s_at	SMC4	structural maintenance of chromosomes 4	3.835	1.939	0.000	0.000
188	207992_s_at	AMPD3	adenosine monophosphate deaminase (isoform E)	3.826	1.936	0.000	0.000
189	216512_s_at	DCT	dopachrome tautomerase (dopachrome delta-isomerase, tyrosine-related protein 2)	3.793	1.923	0.001	0.000
190	207238_s_at	PTPRC	protein tyrosine phosphatase, receptor type, C	3.787	1.921	0.000	0.000
191	205844_at	VNN1	vanin 1	3.780	1.918	0.000	0.000
192	208116_s_at	MAN1A1	mannosidase, alpha, class 1A, member 1	3.780	1.918	0.000	0.000
193	214710_s_at	CCNB1	cyclin B1	3.765	1.913	0.000	0.000
194	210559_s_at	CDC2	cell division cycle 2, G1 to S and G2 to M	3.761	1.911	0.000	0.000
195	201992_s_at	KIF5B	kinesin family member 5B	3.748	1.906	0.000	0.000
196	211561_x_at	MAPK14	mitogen-activated protein kinase 14	3.731	1.900	0.000	0.000
197	214499_s_at	BCLAF1	BCL2-associated transcription factor 1	3.730	1.899	0.000	0.000
198	219669_at	CD177	CD177 molecule	3.716	1.894	0.000	0.000
199	218340_s_at	UBE1L2	ubiquitin-activating enzyme E1-like 2	3.705	1.889	0.000	0.000
200	60474_at	C20orf42	chromosome 20 open reading frame 42	3.693	1.885	0.000	0.000
201	218355_at	KIF4A	kinesin family member 4A	3.690	1.884	0.000	0.000
202	218986_s_at	FLJ20035	hypothetical protein FLJ20035	3.688	1.883	0.000	0.000
203	208436_s_at	IRF7	interferon regulatory factor 7	3.675	1.878	0.000	0.000
204	203716_s_at	DPP4	dipeptidyl-peptidase 4 (CD26, adenosine deaminase complexing protein 2)	3.672	1.876	0.000	0.000
205	211352_s_at	NCOA3	nuclear receptor coactivator 3	3.671	1.876	0.000	0.000
206	205467_at	CASP10	caspase 10, apoptosis-related cysteine peptidase	3.668	1.875	0.000	0.000
207	202269_x_at	GBP1	guanylate binding protein 1, interferon-inducible, 67kDa	3.660	1.872	0.000	0.000
208	211547_s_at	PAFAH1B1	platelet-activating factor acetylhydrolase, isoform Ib, alpha subunit 45kDa	3.655	1.870	0.000	0.000
209	212720_at	PAPOLA	poly(A) polymerase alpha	3.650	1.868	0.000	0.000
210	202446_s_at	PLSCR1	phospholipid scramblase 1	3.630	1.860	0.000	0.000
211	203641_s_at	COBLL1	COBL-like 1	3.622	1.857	0.000	0.000
212	201130_s_at	CDH1	cadherin 1, type 1, E-cadherin (epithelial)	3.614	1.854	0.005	0.010

	Probe	Symbol	Description	FCH-Scalp-LS-Normal	log2FCH-Scalp-LS-Normal	p-Scalp-LS-Normal	FDR-Scalp-LS-Normal
213	205321_at	EIF2S3	eukaryotic translation initiation factor 2, subunit 3 gamma, 52kDa	3.607	1.851	0.018	0.040
214	218542_at	CEP55	centrosomal protein 55kDa	3.603	1.849	0.000	0.000
215	205476_at	CCL20	chemokine (C-C motif) ligand 20	3.602	1.849	0.006	0.020
216	211075_s_at	CD47	CD47 molecule	3.595	1.846	0.000	0.000
217	202479_s_at	TRIB2	tribbles homolog 2 (Drosophila)	3.593	1.845	0.000	0.000
218	213577_at	SQLE	squalene epoxidase	3.589	1.843	0.000	0.000
219	204614_at	SERPINB2	serpin peptidase inhibitor, clade B (ovalbumin), member 2	3.582	1.841	0.000	0.000
220	214869_x_at	GAPVD1	GTPase activating protein and VPS9 domains 1	3.582	1.841	0.000	0.000
221	204751_x_at	DSC2	desmocollin 2	3.566	1.834	0.000	0.000
222	218856_at	TNFRSF21	tumor necrosis factor receptor superfamily, member 21	3.565	1.834	0.000	0.000
223	206653_at	POLR3G	polymerase (RNA) III (DNA directed) polypeptide G (32kD)	3.548	1.827	0.000	0.000
224	209456_s_at	FBXW11	F-box and WD repeat domain containing 11	3.545	1.826	0.000	0.000
225	212514_x_at	DDX3X	DEAD (Asp-Glu-Ala-Asp) box polypeptide 3, X-linked	3.542	1.825	0.000	0.000
226	202543_s_at	GMFB	glia maturation factor, beta	3.525	1.818	0.000	0.000
227	209921_at	SLC7A11	solute carrier family 7, (cationic amino acid transporter, y+ system) member 11	3.523	1.817	0.000	0.000
228	203535_at	S100A9	S100 calcium binding protein A9	3.506	1.810	0.003	0.010
229	219099_at	C12orf5	chromosome 12 open reading frame 5	3.499	1.807	0.000	0.000
230	209201_x_at	CXCR4	chemokine (C-X-C motif) receptor 4	3.492	1.804	0.000	0.000
231	213457_at	MFHAS1	malignant fibrous histiocytoma amplified sequence 1	3.492	1.804	0.000	0.000
232	215236_s_at	PICALM	phosphatidylinositol binding clathrin assembly protein	3.467	1.794	0.000	0.000
233	206008_at	TGM1	transglutaminase 1 (K polypeptide epidermal type I, protein-glutamine-gamma-glutamyltransferase)	3.460	1.791	0.000	0.000
234	210449_x_at	MAPK14	mitogen-activated protein kinase 14	3.443	1.783	0.000	0.000
235	214070_s_at	ATP10B	ATPase, Class V, type 10B	3.442	1.783	0.000	0.000
236	203148_s_at	TRIM14	tripartite motif-containing 14	3.432	1.779	0.000	0.000
237	204527_at	MYO5A	myosin VA (heavy chain 12, myoxin)	3.429	1.778	0.000	0.000
238	204033_at	TRIP13	thyroid hormone receptor interactor 13	3.426	1.776	0.000	0.000
239	207382_at	TP63	tumor protein p63	3.404	1.767	0.000	0.000
240	215719_x_at	FAS	Fas (TNF receptor superfamily, member 6)	3.399	1.765	0.000	0.000
241	202765_s_at	FBN1	fibrillin 1	3.395	1.764	0.000	0.000
242	208539_x_at	SPRR2D	small proline-rich protein 2D	3.395	1.763	0.000	0.000
243	203418_at	CCNA2	cyclin A2	3.386	1.760	0.000	0.000
244	211801_x_at	MFN1	mitofusin 1	3.377	1.756	0.000	0.000
245	219918_s_at	ASPM	asp (abnormal spindle) homolog, microcephaly associated (Drosophila)	3.376	1.755	0.000	0.000
246	212008_at	UBXD2	UBX domain containing 2	3.375	1.755	0.000	0.000
247	214975_s_at	MTMR1	myotubularin related protein 1	3.374	1.754	0.001	0.000
248	216252_x_at	FAS	Fas (TNF receptor superfamily, member 6)	3.373	1.754	0.000	0.000
249	212016_s_at	PTBP1	polypyrimidine tract binding protein 1	3.360	1.748	0.000	0.000
250	209727_at	GM2A	GM2 ganglioside activator	3.356	1.747	0.000	0.000
251	209825_s_at	UCK2	uridine-cytidine kinase 2	3.336	1.738	0.000	0.000
252	203974_at	HDHD1A	haloacid dehalogenase-like hydrolase domain containing 1A	3.334	1.737	0.000	0.000
253	204440_at	CD83	CD83 molecule	3.328	1.735	0.000	0.000
254	205909_at	POLE2	polymerase (DNA directed), epsilon 2 (p59 subunit)	3.323	1.732	0.000	0.000
255	220745_at	IL19	interleukin 19	3.322	1.732	0.006	0.010
256	202134_s_at	WWTR1	WW domain containing transcription regulator 1	3.295	1.720	0.000	0.000
257	204769_s_at	TAP2	transporter 2, ATP-binding cassette, sub-family B (MDR/TAP)	3.294	1.720	0.000	0.000
258	201299_s_at	MOBKL1B	MOB1, Mps One Binder kinase activator-like 1B (yeast)	3.293	1.719	0.000	0.000
259	213293_s_at	TRIM22	tripartite motif-containing 22	3.293	1.719	0.000	0.000
260	215936_s_at	KIAA1033	KIAA1033	3.282	1.715	0.000	0.000
261	220342_x_at	EDEM3	ER degradation enhancer, mannosidase alpha-like 3	3.271	1.710	0.000	0.000
262	217109_at	MUC4	mucin 4, cell surface associated	3.264	1.707	0.000	0.000
263	214786_at	MAP3K1	mitogen-activated protein kinase kinase kinase 1	3.247	1.699	0.000	0.000
265	209895_at	PTPN11	protein tyrosine phosphatase, non-receptor type 11 (Noonan syndrome 1)	3.238	1.695	0.000	0.000
266	218796_at	C20orf42	chromosome 20 open reading frame 42	3.235	1.694	0.000	0.000
267	219209_at	IFIH1	interferon induced with helicase C domain 1	3.228	1.691	0.000	0.000
268	210385_s_at	ARTS-1	type 1 tumor necrosis factor receptor shedding aminopeptidase regulator	3.222	1.688	0.000	0.000
269	209976_s_at	CYP2E1	cytochrome P450, family 2, subfamily E, polypeptide 1	3.219	1.687	0.000	0.000
270	207463_x_at	PRSS3	protease, serine, 3 (mesotrypsin)	3.218	1.686	0.000	0.000
271	212365_at	MYO1B	myosin IB	3.217	1.686	0.000	0.000
272	206125_s_at	KLK8	kallikrein-related peptidase 8	3.215	1.685	0.000	0.000
273	217739_s_at	PBEF1	pre-B-cell colony enhancing factor 1	3.213	1.684	0.000	0.000
274	202827_s_at	MMP14	matrix metallopeptidase 14 (membrane-inserted)	3.204	1.680	0.000	0.000
275	200887_s_at	STAT1	signal transducer and activator of transcription 1, 91kDa	3.202	1.679	0.000	0.000
276	205829_at	HSD17B1	hydroxysteroid (17-beta) dehydrogenase 1	3.199	1.678	0.000	0.000
277	202600_s_at	NRIP1	nuclear receptor interacting protein 1	3.194	1.676	0.001	0.000
278	209975_at	CYP2E1	cytochrome P450, family 2, subfamily E, polypeptide 1	3.174	1.666	0.002	0.000
279	209055_s_at	CDC5L	CDC5 cell division cycle 5-like (S. pombe)	3.173	1.666	0.000	0.000
280	217763_s_at	RAB31	RAB31, member RAS oncogene family	3.173	1.666	0.000	0.000
281	201044_x_at	DUSP1	dual specificity phosphatase 1	3.172	1.666	0.000	0.000
282	217028_at	CXCR4	chemokine (C-X-C motif) receptor 4	3.169	1.664	0.000	0.000
283	206025_s_at	TNFAIP6	tumor necrosis factor, alpha-induced protein 6	3.168	1.664	0.004	0.010
284	204641_at	NEK2	NIMA (never in mitosis gene a)-related kinase 2	3.159	1.659	0.000	0.000
285	214446_at	ELL2	elongation factor, RNA polymerase II, 2	3.148	1.654	0.000	0.000

	Probe	Symbol	Description	FCH-Scalp-LS-Normal	log2FCH-Scalp-LS-Normal	p-Scalp-LS-Normal	FDR-Scalp-LS-Normal
286	214483_s_at	ARFIP1	ADP-ribosylation factor interacting protein 1 (arfaptin 1)	3.143	1.652	0.000	0.000
287	214691_x_at	FAM63B	family with sequence similarity 63, member B	3.142	1.652	0.000	0.000
288	206113_s_at	RAB5A	RAB5A, member RAS oncogene family	3.134	1.648	0.000	0.000
289	204240_s_at	SMC2	structural maintenance of chromosomes 2	3.133	1.648	0.000	0.000
290	202625_at	LYN	v-yes-1 Yamaguchi sarcoma viral related oncogene homolog	3.127	1.645	0.000	0.000
291	214536_at	SLURP1	secreted LY6/PLAUR domain containing 1	3.123	1.643	0.000	0.000
292	211361_s_at	SERPINB13	serpin peptidase inhibitor, clade B (ovalbumin), member 13	3.121	1.642	0.000	0.000
293	213131_at	OLFM1	olfactomedin 1	3.120	1.641	0.000	0.000
294	205159_at	CSF2RB	colony stimulating factor 2 receptor, beta, low-affinity (granulocyte-macrophage)	3.116	1.640	0.000	0.000
295	220386_s_at	EML4	echinoderm microtubule associated protein like 4	3.115	1.639	0.001	0.000
296	212102_s_at	KPNA6	karyopherin alpha 6 (importin alpha 7)	3.113	1.638	0.000	0.000
297	210608_s_at	FUT2	fucosyltransferase 2 (secretor status included)	3.112	1.638	0.000	0.000
298	206094_x_at	UGT1A6	UDP glucuronosyltransferase 1 family, polypeptide A6	3.107	1.636	0.000	0.000
299	213707_s_at	DLX5	distal-less homeobox 5	3.105	1.635	0.000	0.000
300	220941_s_at	C21orf91	chromosome 21 open reading frame 91	3.103	1.633	0.000	0.000
301	211478_s_at	DPP4	dipeptidyl-peptidase 4 (CD26, adenosine deaminase complexing protein 2)	3.100	1.632	0.000	0.000
302	201196_s_at	AMD1	adenosylmethionine decarboxylase 1	3.082	1.624	0.000	0.000
303	218295_s_at	NUP50	nucleoporin 50kDa	3.063	1.615	0.000	0.000
304	221305_s_at	UGT1A8	UDP glucuronosyltransferase 1 family, polypeptide A8	3.061	1.614	0.002	0.000
305	219225_at	PGBD5	piggyBac transposable element derived 5	3.055	1.611	0.000	0.000
306	204748_at	PTGS2	prostaglandin-endoperoxide synthase 2 (prostaglandin G/H synthase and cyclooxygenase)	3.045	1.606	0.001	0.000
307	200607_s_at	RAD21	RAD21 homolog (S. pombe)	3.038	1.603	0.000	0.000
308	1431_at	CYP2E1	cytochrome P450, family 2, subfamily E, polypeptide 1	3.034	1.601	0.000	0.000
309	202412_s_at	USP1	ubiquitin specific peptidase 1	3.029	1.599	0.000	0.000
310	209772_s_at	CD24	CD24 molecule	3.028	1.598	0.000	0.000
311	204891_s_at	LCK	lymphocyte-specific protein tyrosine kinase	3.026	1.597	0.001	0.000
312	201856_s_at	ZFR	zinc finger RNA binding protein	3.022	1.595	0.000	0.000
313	216243_s_at	IL1RN	interleukin 1 receptor antagonist	3.020	1.595	0.000	0.000
314	214895_s_at	ADAM10	ADAM metallopeptidase domain 10	3.007	1.588	0.000	0.000
315	204279_at	PSMB9	proteasome (prosome, macropain) subunit, beta type, 9 (large multifunctional peptidase 2)	3.001	1.585	0.000	0.000
316	203499_at	EPHA2	EPH receptor A2	3.000	1.585	0.000	0.000
317	203888_at	THBD	thrombomodulin	2.999	1.584	0.000	0.000
318	215707_s_at	PRNP	prion protein (p27-30) (Creutzfeldt-Jakob disease, Gerstmann-Strausler-Scheinker syndrome, fatal familial insomnia)	2.999	1.584	0.000	0.000
319	215033_at	TM4SF1	transmembrane 4 L six family member 1	2.997	1.583	0.003	0.010
320	204502_at	SAMHD1	SAM domain and HD domain 1	2.988	1.579	0.000	0.000
321	204162_at	NDC80	NDC80 homolog, kinetochore complex component (S. cerevisiae)	2.980	1.575	0.000	0.000
322	203276_at	LMNB1	lamin B1	2.974	1.572	0.000	0.000
323	210164_at	GZMB	granzyme B (granzyme 2, cytotoxic T-lymphocyte-associated serine esterase 1)	2.971	1.571	0.003	0.000
324	219657_s_at	KLF3	Kruppel-like factor 3 (basic)	2.969	1.570	0.000	0.000
325	204285_s_at	PMAIP1	phorbol-12-myristate-13-acetate-induced protein 1	2.965	1.568	0.000	0.000
326	217678_at	SLC7A11	solute carrier family 7, (cationic amino acid transporter, y+ system) member 11	2.961	1.566	0.000	0.000
327	201101_s_at	BCLAF1	BCL2-associated transcription factor 1	2.957	1.564	0.000	0.000
328	219630_at	PDZK1IP1	PDZK1 interacting protein 1	2.957	1.564	0.000	0.000
329	205287_s_at	TFAP2C	transcription factor AP-2 gamma (activating enhancer binding protein 2 gamma)	2.951	1.561	0.000	0.000
330	201531_at	ZFP36	zinc finger protein 36, C3H type, homolog (mouse)	2.950	1.561	0.000	0.000
331	208309_s_at	MALT1	mucosa associated lymphoid tissue lymphoma translocation gene 1	2.946	1.559	0.000	0.000
332	209237_s_at	SLC23A2	solute carrier family 23 (nucleobase transporters), member 2	2.941	1.557	0.001	0.000
333	203213_at	CDC2	cell division cycle 2, G1 to S and G2 to M	2.931	1.551	0.000	0.000
334	212420_at	ELF1	E74-like factor 1 (ets domain transcription factor)	2.929	1.550	0.000	0.000
335	209761_s_at	SP110	SP110 nuclear body protein	2.927	1.550	0.000	0.000
336	205034_at	CCNE2	cyclin E2	2.922	1.547	0.000	0.000
337	205419_at	EBI2	Epstein-Barr virus induced gene 2 (lymphocyte-specific G protein-coupled receptor)	2.922	1.547	0.000	0.000
338	212286_at	ANKRD12	ankyrin repeat domain 12	2.921	1.547	0.000	0.000
339	204420_at	FOSL1	FOS-like antigen 1	2.917	1.545	0.002	0.000
340	202430_s_at	PLSCR1	phospholipid scramblase 1	2.913	1.543	0.000	0.000
341	204380_s_at	FGFR3	fibroblast growth factor receptor 3 (achondroplasia, thanatophoric dwarfism)	2.906	1.539	0.002	0.000
342	206023_at	NMU	neuromedin U	2.900	1.536	0.000	0.000
343	205831_at	CD2	CD2 molecule	2.892	1.532	0.001	0.000
344	206553_at	OAS2	2'-5'-oligoadenylate synthetase 2, 69/71kDa	2.890	1.531	0.000	0.000
345	204532_x_at	UGT1A9	UDP glucuronosyltransferase 1 family, polypeptide A9	2.889	1.531	0.000	0.000
346	205554_s_at	DNASE1L3	deoxyribonuclease I-like 3	2.886	1.529	0.000	0.000
347	204170_s_at	CKS2	CDC28 protein kinase regulatory subunit 2	2.878	1.525	0.000	0.000
348	218990_s_at	SPRR3	small proline-rich protein 3	2.876	1.524	0.011	0.030
349	202095_s_at	BIRC5	baculoviral IAP repeat-containing 5 (survivin)	2.869	1.520	0.000	0.000
350	210834_s_at	PTGER3	prostaglandin E receptor 3 (subtype EP3)	2.865	1.519	0.000	0.000
351	217764_s_at	RAB31	RAB31, member RAS oncogene family	2.862	1.517	0.000	0.000
352	203471_s_at	PLEK	pleckstrin	2.860	1.516	0.001	0.000
353	203595_s_at	IFIT5	interferon-induced protein with tetratricopeptide repeats 5	2.855	1.513	0.000	0.000
354	202619_s_at	PLOD2	procollagen-lysine, 2-oxoglutarate 5-dioxygenase 2	2.851	1.512	0.000	0.000
355	218817_at	SPCS3	signal peptidase complex subunit 3 homolog (S. cerevisiae)	2.845	1.509	0.000	0.000
356	201508_at	IGFBP4	insulin-like growth factor binding protein 4	2.839	1.505	0.000	0.000
357	204881_s_at	UGCG	UDP-glucose ceramide glucosyltransferase	2.838	1.505	0.000	0.000

	Probe	Symbol	Description	FCH-Scalp-LS-Normal	log2FCH-Scalp-LS-Normal	p-Scalp-LS-Normal	FDR-Scalp-LS-Normal
358	212107_s_at	DHX9	DEAH (Asp-Glu-Ala-His) box polypeptide 9	2.834	1.503	0.001	0.000
359	217497_at	ECGF1	endothelial cell growth factor 1 (platelet-derived)	2.828	1.500	0.000	0.000
360	213933_at	PTGER3	prostaglandin E receptor 3 (subtype EP3)	2.816	1.494	0.000	0.000
361	211339_s_at	ITK	IL2-inducible T-cell kinase	2.811	1.491	0.001	0.000
362	212634_at	KIAA0776	KIAA0776	2.810	1.491	0.000	0.000
363	201473_at	JUNB	jun B proto-oncogene	2.808	1.490	0.000	0.000
364	205798_at	IL7R	interleukin 7 receptor	2.804	1.487	0.001	0.000
365	203128_at	SPTLC2	serine palmitoyltransferase, long chain base subunit 2	2.802	1.486	0.000	0.000
366	207126_x_at	UGT1A1	UDP glucuronosyltransferase 1 family, polypeptide A1	2.802	1.486	0.000	0.000
367	202870_s_at	CDC20	cell division cycle 20 homolog (S. cerevisiae)	2.797	1.484	0.000	0.000
368	216598_s_at	CCL2	chemokine (C-C motif) ligand 2	2.796	1.483	0.000	0.000
369	217272_s_at	SERPINB13	serpin peptidase inhibitor, clade B (ovalbumin), member 13	2.791	1.481	0.000	0.000
370	206026_s_at	TNFAIP6	tumor necrosis factor, alpha-induced protein 6	2.790	1.480	0.006	0.010
371	208567_s_at	KCNJ12	potassium inwardly-rectifying channel, subfamily J, member 12	2.788	1.479	0.000	0.000
372	216399_s_at	SCAPER	S phase cyclin A-associated protein in the ER	2.787	1.479	0.000	0.000
373	221690_s_at	NLRP2	NLR family, pyrin domain containing 2	2.779	1.475	0.019	0.040
374	204258_at	CHD1	chromodomain helicase DNA binding protein 1	2.775	1.472	0.000	0.000
375	203780_at	MPZL2	myelin protein zero-like 2	2.774	1.472	0.000	0.000
376	208867_s_at	CSNK1A1	casein kinase 1, alpha 1	2.773	1.471	0.000	0.000
377	213421_x_at	PRSS3	protease, serine, 3 (mesotrypsin)	2.768	1.469	0.000	0.000
378	217192_s_at	PRDM1	PR domain containing 1, with ZNF domain	2.763	1.466	0.000	0.000
379	209882_at	RIT1	Ras-like without CAAX 1	2.760	1.464	0.000	0.000
380	200731_s_at	PTP4A1	protein tyrosine phosphatase type IVA, member 1	2.755	1.462	0.000	0.000
381	215808_at	KLK10	kallikrein-related peptidase 10	2.754	1.462	0.002	0.000
382	207828_s_at	CENPF	centromere protein F, 350/400ka (mitosin)	2.746	1.457	0.000	0.000
383	213599_at	OIP5	Opa interacting protein 5	2.744	1.456	0.000	0.000
384	215505_s_at	STRN3	striatin, calmodulin binding protein 3	2.740	1.454	0.000	0.000
385	212196_at	IL6ST	interleukin 6 signal transducer (gp130, oncostatin M receptor)	2.738	1.453	0.000	0.000
386	211965_at	ZFP36L1	zinc finger protein 36, C3H type-like 1	2.732	1.450	0.000	0.000
387	210018_x_at	MALT1	mucosa associated lymphoid tissue lymphoma translocation gene 1	2.728	1.448	0.000	0.000
388	220865_s_at	PDSS1	prenyl (decaprenyl) diphosphate synthase, subunit 1	2.728	1.448	0.000	0.000
389	205990_s_at	WNT5A	wingless-type MMTV integration site family, member 5A	2.722	1.445	0.000	0.000
390	209741_x_at	SCAPER	S phase cyclin A-associated protein in the ER	2.720	1.443	0.000	0.000
391	201733_at	CLCN3	chloride channel 3	2.714	1.440	0.000	0.000
392	220066_at	NOD2	nucleotide-binding oligomerization domain containing 2	2.713	1.440	0.000	0.000
393	205809_s_at	WASL	Wiskott-Aldrich syndrome-like	2.703	1.435	0.002	0.000
394	209569_x_at	D4S234E	DNA segment on chromosome 4 (unique) 234 expressed sequence	2.701	1.433	0.000	0.000
395	207386_at	CYP7B1	cytochrome P450, family 7, subfamily B, polypeptide 1	2.700	1.433	0.000	0.000
396	202902_s_at	CTSS	cathepsin S	2.699	1.433	0.000	0.000
397	201367_s_at	ZFP36L2	zinc finger protein 36, C3H type-like 2	2.698	1.432	0.002	0.000
398	201996_s_at	SPEN	spen homolog, transcriptional regulator (Drosophila)	2.696	1.431	0.009	0.020
399	203596_s_at	IFIT5	interferon-induced protein with tetratricopeptide repeats 5	2.695	1.430	0.000	0.000
400	212141_at	MCM4	minichromosome maintenance complex component 4	2.691	1.428	0.000	0.000
401	209040_s_at	PSMB8	proteasome (prosome, macropain) subunit, beta type, 8 (large multifunctional peptidase 7)	2.690	1.428	0.000	0.000
402	212225_at	EIF1	eukaryotic translation initiation factor 1	2.689	1.427	0.000	0.000
403	203256_at	CDH3	cadherin 3, type 1, P-cadherin (placental)	2.687	1.426	0.000	0.000
404	205269_at	LCP2	lymphocyte cytosolic protein 2 (SH2 domain containing leukocyte protein of 76kDa)	2.687	1.426	0.000	0.000
405	201559_s_at	CLIC4	chloride intracellular channel 4	2.686	1.425	0.005	0.010
406	210001_s_at	SOCS1	suppressor of cytokine signaling 1	2.683	1.424	0.000	0.000
407	219544_at	C13orf34	chromosome 13 open reading frame 34	2.681	1.423	0.000	0.000
408	202236_s_at	SLC16A1	solute carrier family 16, member 1 (monocarboxylic acid transporter 1)	2.676	1.420	0.000	0.000
409	211944_at	BAT2D1	BAT2 domain containing 1	2.676	1.420	0.000	0.000
410	205569_at	LAMP3	lysosomal-associated membrane protein 3	2.674	1.419	0.000	0.000
411	211571_s_at	VCAN	versican	2.671	1.417	0.001	0.000
412	201688_s_at	TPD52	tumor protein D52	2.665	1.414	0.000	0.000
413	204822_at	TTK	TTK protein kinase	2.665	1.414	0.000	0.000
414	205014_at	FGFBP1	fibroblast growth factor binding protein 1	2.663	1.413	0.000	0.000
415	205774_at	F12	coagulation factor XII (Hageman factor)	2.663	1.413	0.000	0.000
416	216470_x_at	TRY6	trypsinogen C	2.663	1.413	0.000	0.000
417	206562_s_at	CSNK1A1	casein kinase 1, alpha 1	2.661	1.412	0.000	0.000
418	200604_s_at	PRKAR1A	protein kinase, cAMP-dependent, regulatory, type I, alpha (tissue specific extinguisher 1)	2.659	1.411	0.000	0.000
419	212659_s_at	IL1RN	interleukin 1 receptor antagonist	2.657	1.410	0.000	0.000
420	201537_s_at	DUSP3	dual specificity phosphatase 3 (vaccinia virus phosphatase VH1-related)	2.656	1.409	0.000	0.000
421	211537_x_at	MAP3K7	mitogen-activated protein kinase kinase kinase 7	2.656	1.409	0.000	0.000
422	204750_s_at	DSC2	desmocollin 2	2.653	1.408	0.001	0.000
423	39249_at	AQP3	aquaporin 3 (Gill blood group)	2.652	1.407	0.000	0.000
424	202578_s_at	DDX19A	DEAD (Asp-Glu-Ala-As) box polypeptide 19A	2.650	1.406	0.000	0.000
425	211139_s_at	NAB1	NGFI-A binding protein 1 (EGR1 binding protein 1)	2.650	1.406	0.000	0.000
426	212460_at	C14orf147	chromosome 14 open reading frame 147	2.649	1.405	0.000	0.000
427	219901_at	FGD6	FYVE, RhoGEF and PH domain containing 6	2.647	1.404	0.000	0.000
428	216689_x_at	ARHGAP1	Rho GTPase activating protein 1	2.645	1.403	0.000	0.000
429	214038_at	CCL8	chemokine (C-C motif) ligand 8	2.644	1.403	0.000	0.000

	Probe	Symbol	Description	FCH-Scalp-LS-Normal	log2FCH-Scalp-LS-Normal	p-Scalp-LS-Normal	FDR-Scalp-LS-Normal
430	203882_at	ISGF3G	interferon-stimulated transcription factor 3, gamma 48kDa	2.643	1.402	0.000	0.000
431	210732_s_at	LGALS8	lectin, galactoside-binding, soluble, 8 (galectin 8)	2.641	1.401	0.000	0.000
432	202575_at	CRABP2	cellular retinoic acid binding protein 2	2.633	1.397	0.000	0.000
433	209408_at	KIF2C	kinesin family member 2C	2.632	1.396	0.000	0.000
434	212379_at	GART	phosphoribosylglycinamide formyltransferase, phosphoribosylglycinamide synthetase, phosphoribosylaminoimidazole synthetase	2.627	1.394	0.000	0.000
435	201641_at	BST2	bone marrow stromal cell antigen 2	2.625	1.392	0.003	0.010
436	204601_at	N4BP1	Nedd4 binding protein 1	2.624	1.392	0.000	0.000
437	205127_at	PTGS1	prostaglandin-endoperoxide synthase 1 (prostaglandin G/H synthase and cyclooxygenase)	2.621	1.390	0.002	0.000
438	208966_x_at	IFI16	interferon, gamma-inducible protein 16	2.619	1.389	0.000	0.000
439	209041_s_at	UBE2G2	ubiquitin-conjugating enzyme E2G 2 (UBC7 homolog, yeast)	2.610	1.384	0.000	0.000
440	206109_at	FUT1	fucosyltransferase 1 (galactoside 2-alpha-L-fucosyltransferase, H blood group)	2.608	1.383	0.000	0.000
441	203887_s_at	THBD	thrombomodulin	2.603	1.380	0.000	0.000
442	213226_at	CCNA2	cyclin A2	2.603	1.380	0.000	0.000
443	219461_at	PAK6	p21(CDKN1A)-activated kinase 6	2.603	1.380	0.000	0.000
444	202831_at	GPX2	glutathione peroxidase 2 (gastrointestinal)	2.601	1.379	0.000	0.000
445	212978_at	LRRC8B	leucine rich repeat containing 8 family, member B	2.595	1.376	0.000	0.000
446	213986_s_at	C19orf6	chromosome 19 open reading frame 6	2.588	1.372	0.002	0.000
447	218095_s_at	TMEM165	transmembrane protein 165	2.588	1.372	0.000	0.000
448	221618_s_at	TAF9B	TAF9B RNA polymerase II, TATA box binding protein (TBP)-associated factor, 31kDa	2.588	1.372	0.001	0.000
449	200798_x_at	MCL1	myeloid cell leukemia sequence 1 (BCL2-related)	2.584	1.370	0.000	0.000
450	202431_s_at	MYC	v-myc myelocytomatosis viral oncogene homolog (avian)	2.580	1.368	0.000	0.000
451	201732_s_at	CLCN3	chloride channel 3	2.579	1.367	0.000	0.000
452	219158_s_at	NARG1	NMDA receptor regulated 1	2.576	1.365	0.000	0.000
453	212385_at	TCF4	transcription factor 4	2.574	1.364	0.000	0.000
454	221113_s_at	WNT16	wingless-type MMTV integration site family, member 16	2.573	1.364	0.000	0.000
455	218273_s_at	PPM2C	protein phosphatase 2C, magnesium-dependent, catalytic subunit	2.569	1.361	0.000	0.000
456	201720_s_at	LAPTM5	lysosomal associated multispanning membrane protein 5	2.564	1.358	0.000	0.000
457	212808_at	NFATC2IP	nuclear factor of activated T-cells, cytoplasmic, calcineurin-dependent 2 interacting protein	2.564	1.358	0.000	0.000
458	203362_s_at	MAD2L1	MAD2 mitotic arrest deficient-like 1 (yeast)	2.559	1.355	0.000	0.000
459	210873_x_at	APOBEC3A	apolipoprotein B mRNA editing enzyme, catalytic polypeptide-like 3A	2.559	1.355	0.011	0.030
460	213470_s_at	HNRPH1	heterogeneous nuclear ribonucleoprotein H1 (H)	2.550	1.351	0.001	0.000
461	204698_at	ISG20	interferon stimulated exonuclease gene 20kDa	2.549	1.350	0.000	0.000
462	203964_at	NMI	N-myc (and STAT) interactor	2.545	1.348	0.000	0.000
463	205240_at	GPSM2	G-protein signaling modulator 2 (AGS3-like, C. elegans)	2.534	1.341	0.000	0.000
464	219148_at	PBK	PDZ binding kinase	2.530	1.339	0.000	0.000
465	200769_s_at	MAT2A	methionine adenosyltransferase II, alpha	2.526	1.337	0.001	0.000
466	209281_s_at	ATP2B1	ATPase, Ca++ transporting, plasma membrane 1	2.525	1.336	0.000	0.000
467	203476_at	TPBG	trophoblast glycoprotein	2.524	1.336	0.000	0.000
468	206276_at	LY6D	lymphocyte antigen 6 complex, locus D	2.523	1.335	0.000	0.000
470	210186_s_at	FKBP1A	FK506 binding protein 1A, 12kDa	2.522	1.334	0.000	0.000
471	220026_at	CLCA4	chloride channel, calcium activated, family member 4	2.515	1.330	0.001	0.000
472	221107_at	CHRNA9	cholinergic receptor, nicotinic, alpha 9	2.515	1.331	0.009	0.020
473	222383_s_at	ALOXE3	arachidonate lipoxygenase 3	2.512	1.329	0.000	0.000
474	202064_s_at	SEL1L	sel-1 suppressor of lin-12-like (C. elegans)	2.510	1.328	0.000	0.000
475	211668_s_at	PLAU	plasminogen activator, urokinase	2.498	1.321	0.000	0.000
476	206504_at	CYP24A1	cytochrome P450, family 24, subfamily A, polypeptide 1	2.493	1.318	0.000	0.000
477	219787_s_at	ECT2	epithelial cell transforming sequence 2 oncogene	2.489	1.315	0.000	0.000
478	204622_x_at	NR4A2	nuclear receptor subfamily 4, group A, member 2	2.483	1.312	0.017	0.040
479	206765_at	KCNJ2	potassium inwardly-rectifying channel, subfamily J, member 2	2.480	1.311	0.000	0.000
480	208900_s_at	TOP1	topoisomerase (DNA) I	2.480	1.310	0.000	0.000
481	208721_s_at	ANAPC5	anaphase promoting complex subunit 5	2.479	1.310	0.000	0.000
482	206332_s_at	IFI16	interferon, gamma-inducible protein 16	2.474	1.307	0.000	0.000
483	211578_s_at	RPS6KB1	ribosomal protein S6 kinase, 70kDa, polypeptide 1	2.470	1.304	0.000	0.000
484	212574_x_at	C19orf6	chromosome 19 open reading frame 6	2.465	1.302	0.001	0.000
485	206247_at	MICB	MHC class I polypeptide-related sequence B	2.462	1.300	0.000	0.000
486	218782_s_at	ATAD2	ATPase family, AAA domain containing 2	2.461	1.299	0.000	0.000
487	201742_x_at	SFRS1	splicing factor, arginine/serine-rich 1 (splicing factor 2, alternate splicing factor)	2.457	1.297	0.000	0.000
488	206668_s_at	SCAMP1	secretory carrier membrane protein 1	2.456	1.296	0.000	0.000
489	202805_s_at	ABCC1	ATP-binding cassette, sub-family C (CFTR/MRP), member 1	2.453	1.295	0.000	0.000
490	212002_at	C1orf144	chromosome 1 open reading frame 144	2.452	1.294	0.000	0.000
491	219279_at	DOCK10	dedicator of cytokinesis 10	2.449	1.292	0.000	0.000
492	212022_s_at	MKI67	antigen identified by monoclonal antibody Ki-67	2.448	1.291	0.000	0.000
493	208047_s_at	NAB1	NGFI-A binding protein 1 (EGR1 binding protein 1)	2.445	1.290	0.000	0.000
494	204823_at	NAV3	neuron navigator 3	2.440	1.287	0.000	0.000
495	201422_at	IFI30	interferon, gamma-inducible protein 30	2.439	1.287	0.000	0.000
496	213320_at	PRMT3	protein arginine methyltransferase 3	2.439	1.286	0.000	0.000
497	204863_s_at	IL6ST	interleukin 6 signal transducer (gp130, oncostatin M receptor)	2.438	1.286	0.001	0.000
498	216804_s_at	PDLIM5	PDZ and LIM domain 5	2.438	1.286	0.000	0.000
499	206409_at	TIAM1	T-cell lymphoma invasion and metastasis 1	2.435	1.284	0.000	0.000
500	212588_at	PTPRC	protein tyrosine phosphatase, receptor type, C	2.434	1.283	0.001	0.000
501	203180_at	ALDH1A3	aldehyde dehydrogenase 1 family, member A3	2.432	1.282	0.005	0.010
502	213857_s_at	CD47	CD47 molecule	2.432	1.282	0.000	0.000

	Probe	Symbol	Description	FCH-Scalp-LS-Normal	log2FCH-Scalp-LS-Normal	p-Scalp-LS-Normal	FDR-Scalp-LS-Normal
503	201523_x_at	UBE2N	ubiquitin-conjugating enzyme E2N (UBC13 homolog, yeast)	2.426	1.279	0.000	0.000
504	208433_s_at	LRP8	low density lipoprotein receptor-related protein 8, apolipoprotein e receptor	2.426	1.279	0.000	0.000
505	202659_at	PSMB10	proteasome (prosome, macropain) subunit, beta type, 10	2.425	1.278	0.000	0.000
506	211113_s_at	ABCG1	ATP-binding cassette, sub-family G (WHITE), member 1	2.420	1.275	0.000	0.000
507	211450_s_at	MSH6	mutS homolog 6 (E. coli)	2.416	1.273	0.000	0.000
508	218947_s_at	PAPD1	PAP associated domain containing 1	2.416	1.273	0.000	0.000
509	221884_at	EVI1	ecotropic viral integration site 1	2.415	1.272	0.000	0.000
510	209024_s_at	SYNCRIP	synaptotagmin binding, cytoplasmic RNA interacting protein	2.414	1.272	0.000	0.000
511	200922_at	KDELR1	KDEL (Lys-Asp-Glu-Leu) endoplasmic reticulum protein retention receptor 1	2.412	1.270	0.000	0.000
512	203747_at	AQP3	aquaporin 3 (Gill blood group)	2.409	1.268	0.000	0.000
513	206858_s_at	HOXC6	homeobox C6	2.407	1.268	0.002	0.000
514	202604_x_at	ADAM10	ADAM metallopeptidase domain 10	2.404	1.266	0.000	0.000
515	214829_at	AASS	aminoadipate-semialdehyde synthase	2.403	1.265	0.000	0.000
516	208103_s_at	ANP32E	acidic (leucine-rich) nuclear phosphoprotein 32 family, member E	2.401	1.264	0.000	0.000
517	209744_x_at	ITCH	itchy homolog E3 ubiquitin protein ligase (mouse)	2.399	1.262	0.000	0.000
518	202478_at	TRIB2	tribbles homolog 2 (Drosophila)	2.398	1.262	0.000	0.000
519	201686_x_at	API5	apoptosis inhibitor 5	2.397	1.261	0.000	0.000
520	206061_s_at	DICER1	Dicer1, Dcr-1 homolog (Drosophila)	2.397	1.261	0.001	0.000
521	216388_s_at	LTB4R	leukotriene B4 receptor	2.397	1.261	0.000	0.000
522	200641_s_at	YWHAZ	tyrosine 3-monooxygenase/tryptophan 5-monooxygenase activation protein, zeta polypeptide	2.393	1.259	0.000	0.000
523	201514_s_at	G3BP1	GTPase activating protein (SH3 domain) binding protein 1	2.387	1.255	0.000	0.000
524	211536_x_at	MAP3K7	mitogen-activated protein kinase kinase kinase 7	2.385	1.254	0.000	0.000
525	217738_at	PBEF1	pre-B-cell colony enhancing factor 1	2.385	1.254	0.000	0.000
526	219079_at	CYB5R4	cytochrome b5 reductase 4	2.381	1.252	0.000	0.000
527	201859_at	SRGN	serglycin	2.379	1.250	0.000	0.000
528	203936_s_at	MMP9	matrix metallopeptidase 9 (gelatinase B, 92kDa gelatinase, 92kDa type IV collagenase)	2.371	1.245	0.005	0.010
529	201649_at	UBE2L6	ubiquitin-conjugating enzyme E2L 6	2.367	1.243	0.000	0.000
530	209000_s_at	SEPT8	septin 8	2.367	1.243	0.000	0.000
531	217430_x_at	COL1A1	collagen, type I, alpha 1	2.366	1.243	0.002	0.000
532	213523_at	CCNE1	cyclin E1	2.365	1.242	0.000	0.000
533	209653_at	KPNA4	karyopherin alpha 4 (importin alpha 3)	2.363	1.241	0.000	0.000
534	209821_at	IL33	interleukin 33	2.363	1.241	0.000	0.000
535	220202_s_at	RC3H2	ring finger and CCCH-type zinc finger domains 2	2.363	1.241	0.000	0.000
536	204224_s_at	GCH1	GTP cyclohydrolase 1 (dopa-responsive dystonia)	2.362	1.240	0.000	0.000
537	206336_at	CXCL6	chemokine (C-X-C motif) ligand 6 (granulocyte chemotactic protein 2)	2.361	1.239	0.012	0.030
538	209218_at	SQLE	squalene epoxidase	2.357	1.237	0.000	0.000
539	211559_s_at	CCNG2	cyclin G2	2.357	1.237	0.012	0.030
540	201476_s_at	RRM1	ribonucleotide reductase M1 polypeptide	2.356	1.236	0.000	0.000
541	215646_s_at	VCAN	versican	2.356	1.236	0.005	0.010
542	202643_s_at	TNFAIP3	tumor necrosis factor, alpha-induced protein 3	2.354	1.235	0.000	0.000
543	210087_s_at	MPZL1	myelin protein zero-like 1	2.353	1.234	0.000	0.000
544	209629_s_at	NXT2	nuclear transport factor 2-like export factor 2	2.347	1.231	0.005	0.010
545	217173_s_at	LDLR	low density lipoprotein receptor (familial hypercholesterolemia)	2.345	1.229	0.002	0.000
546	202954_at	UBE2C	ubiquitin-conjugating enzyme E2C	2.342	1.228	0.000	0.000
547	220892_s_at	PSAT1	phosphoserine aminotransferase 1	2.341	1.227	0.000	0.000
548	219358_s_at	CENTA2	centaurin, alpha 2	2.338	1.226	0.000	0.000
549	202089_s_at	SLC39A6	solute carrier family 39 (zinc transporter), member 6	2.335	1.224	0.000	0.000
550	221039_s_at	DDEF1	development and differentiation enhancing factor 1	2.330	1.220	0.000	0.000
551	204026_s_at	ZWINT	ZW10 interactor	2.324	1.217	0.000	0.000
552	207700_s_at	NCOA3	nuclear receptor coactivator 3	2.320	1.214	0.000	0.000
553	216244_at	IL1RN	interleukin 1 receptor antagonist	2.318	1.213	0.000	0.000
554	217762_s_at	RAB31	RAB31, member RAS oncogene family	2.318	1.213	0.000	0.000
555	209762_x_at	SP110	SP110 nuclear body protein	2.315	1.211	0.000	0.000
556	205394_at	CHEK1	CHK1 checkpoint homolog (S. pombe)	2.313	1.210	0.000	0.000
557	206098_at	ZBTB6	zinc finger and BTB domain containing 6	2.313	1.209	0.000	0.000
558	204825_at	MELK	maternal embryonic leucine zipper kinase	2.303	1.204	0.000	0.000
559	211726_s_at	FMO2	flavin containing monooxygenase 2 (non-functional)	2.303	1.204	0.010	0.020
560	200900_s_at	M6PR	mannose-6-phosphate receptor (cation dependent)	2.302	1.203	0.000	0.000
561	207018_s_at	RAB27B	RAB27B, member RAS oncogene family	2.301	1.202	0.000	0.000
562	204989_s_at	ITGB4	integrin, beta 4	2.300	1.201	0.006	0.010
563	209949_at	NCF2	neutrophil cytosolic factor 2 (65kDa, chronic granulomatous disease, autosomal 2)	2.291	1.196	0.000	0.000
564	219296_at	ZDHHC13	zinc finger, DHHC-type containing 13	2.291	1.196	0.000	0.000
565	207540_s_at	SYK	spleen tyrosine kinase	2.290	1.196	0.000	0.000
566	215465_at	ABCA12	ATP-binding cassette, sub-family A (ABC1), member 12	2.289	1.195	0.000	0.000
567	217881_s_at	CDC27	cell division cycle 27 homolog (S. cerevisiae)	2.289	1.195	0.000	0.000
568	213927_at	MAP3K9	mitogen-activated protein kinase kinase kinase 9	2.288	1.194	0.000	0.000
569	208012_x_at	SP110	SP110 nuclear body protein	2.287	1.193	0.000	0.000
570	32069_at	N4BP1	Nedd4 binding protein 1	2.284	1.192	0.000	0.000
571	202687_s_at	TNFSF10	tumor necrosis factor (ligand) superfamily, member 10	2.281	1.189	0.000	0.000
572	213373_s_at	CASP8	caspase 8, apoptosis-related cysteine peptidase	2.280	1.189	0.000	0.000
573	213872_at	C6orf62	chromosome 6 open reading frame 62	2.279	1.188	0.006	0.020
574	211672_s_at	ARPC4	actin related protein 2/3 complex, subunit 4, 20kDa	2.277	1.187	0.009	0.020

	Probe	Symbol	Description	FCH-Scalp-LS-Normal	log2FCH-Scalp-LS-Normal	p-Scalp-LS-Normal	FDR-Scalp-LS-Normal
575	201070_x_at	SF3B1	splicing factor 3b, subunit 1, 155kDa	2.274	1.185	0.007	0.020
576	204612_at	PKIA	protein kinase (cAMP-dependent, catalytic) inhibitor alpha	2.272	1.184	0.000	0.000
577	210639_s_at	ATG5	ATG5 autophagy related 5 homolog (S. cerevisiae)	2.269	1.182	0.001	0.000
578	211676_s_at	IFNGR1	interferon gamma receptor 1	2.267	1.181	0.000	0.000
579	206082_at	HCP5	HLA complex P5	2.266	1.180	0.000	0.000
580	219326_s_at	B3GNT2	UDP-GlcNAc:betaGal beta-1,3-N-acetylglucosaminyltransferase 2	2.262	1.178	0.000	0.000
581	222158_s_at	C1orf121	chromosome 1 open reading frame 121	2.258	1.175	0.001	0.000
582	213172_at	TTC9	tetratricopeptide repeat domain 9	2.256	1.173	0.000	0.000
583	203923_s_at	CYBB	cytochrome b-245, beta polypeptide (chronic granulomatous disease)	2.251	1.171	0.008	0.020
584	214651_s_at	HOXA9	homeobox A9	2.251	1.171	0.003	0.010
585	205190_at	PLS1	plastin 1 (I isoform)	2.244	1.166	0.000	0.000
586	205590_at	RASGRP1	RAS guanyl releasing protein 1 (calcium and DAG-regulated)	2.242	1.165	0.000	0.000
587	218755_at	KIF20A	kinesin family member 20A	2.241	1.164	0.000	0.000
588	202705_at	CCNB2	cyclin B2	2.240	1.164	0.000	0.000
589	215760_s_at	SBNO2	strawberry notch homolog 2 (Drosophila)	2.240	1.163	0.000	0.000
590	208992_s_at	STAT3	signal transducer and activator of transcription 3 (acute-phase response factor)	2.238	1.162	0.000	0.000
591	208328_s_at	MEF2A	myocyte enhancer factor 2A	2.236	1.161	0.000	0.000
592	212794_s_at	KIAA1033	KIAA1033	2.235	1.160	0.000	0.000
593	201504_s_at	TSN	translin	2.230	1.157	0.000	0.000
594	221520_s_at	CDCA8	cell division cycle associated 8	2.229	1.156	0.000	0.000
595	219858_s_at	FLJ20160	FLJ20160 protein	2.226	1.154	0.001	0.000
596	204969_s_at	RDX	radixin	2.225	1.154	0.000	0.000
597	218009_s_at	PRC1	protein regulator of cytokinesis 1	2.222	1.152	0.000	0.000
598	213007_at	FANCI	Fanconi anemia, complementation group I	2.221	1.151	0.000	0.000
599	205220_at	GPR109B	G protein-coupled receptor 109B	2.220	1.151	0.000	0.000
600	213975_s_at	LYZ	lysozyme (renal amyloidosis)	2.220	1.151	0.000	0.000
601	214544_s_at	SNAP23	synaptosomal-associated protein, 23kDa	2.217	1.149	0.001	0.000
602	209093_s_at	GBA	glucosidase, beta; acid (includes glucosylceramidase)	2.215	1.147	0.000	0.000
603	218006_s_at	ZNF22	zinc finger protein 22 (KOX 15)	2.214	1.146	0.001	0.000
604	209159_s_at	NDRG4	NDRG family member 4	2.213	1.146	0.000	0.000
605	221477_s_at	MGC5618	hypothetical protein MGC5618	2.213	1.146	0.000	0.000
606	209872_s_at	PKP3	plakophilin 3	2.201	1.138	0.016	0.040
607	219580_s_at	TMC5	transmembrane channel-like 5	2.199	1.137	0.002	0.000
608	202638_s_at	ICAM1	intercellular adhesion molecule 1 (CD54), human rhinovirus receptor	2.198	1.136	0.000	0.000
609	220926_s_at	EDEM3	ER degradation enhancer, mannosidase alpha-like 3	2.198	1.136	0.000	0.000
610	204768_s_at	FEN1	flap structure-specific endonuclease 1	2.197	1.136	0.000	0.000
611	203755_at	BUB1B	BUB1 budding uninhibited by benzimidazoles 1 homolog beta (yeast)	2.196	1.135	0.000	0.000
612	219956_at	GALNT6	UDP-N-acetyl-alpha-D-galactosamine:polypeptide N-acetylgalactosaminyltransferase 6 (GalNAc-T6)	2.193	1.133	0.004	0.010
613	210592_s_at	SAT1	spermidine/spermine N1-acetyltransferase 1	2.192	1.132	0.000	0.000
614	200787_s_at	PEA15	phosphoprotein enriched in astrocytes 15	2.191	1.132	0.000	0.000
615	202129_s_at	RIOK3	RIO kinase 3 (yeast)	2.191	1.132	0.000	0.000
616	203455_s_at	SAT1	spermidine/spermine N1-acetyltransferase 1	2.191	1.131	0.000	0.000
617	205241_at	SCO2	SCO cytochrome oxidase deficient homolog 2 (yeast)	2.189	1.130	0.000	0.000
618	209754_s_at	TMPO	thymopoietin	2.188	1.129	0.002	0.000
619	206075_s_at	CSNK2A1	casein kinase 2, alpha 1 polypeptide	2.179	1.124	0.000	0.000
620	211150_s_at	DLAT	dihydrolipoamide S-acetyltransferase (E2 component of pyruvate dehydrogenase complex)	2.179	1.124	0.000	0.000
621	212325_at	LIMCH1	LIM and calponin homology domains 1	2.179	1.124	0.000	0.000
622	209188_x_at	DR1	down-regulator of transcription 1, TBP-binding (negative cofactor 2)	2.177	1.122	0.000	0.000
623	203504_s_at	ABCA1	ATP-binding cassette, sub-family A (ABC1), member 1	2.174	1.120	0.000	0.000
624	207724_s_at	SPAST	spastin	2.174	1.120	0.002	0.000
625	208930_s_at	ILF3	interleukin enhancer binding factor 3, 90kDa	2.174	1.120	0.000	0.000
626	205836_s_at	YTHDC2	YTH domain containing 2	2.172	1.119	0.000	0.000
627	206337_at	CCR7	chemokine (C-C motif) receptor 7	2.172	1.119	0.000	0.000
628	220172_at	C2orf37	chromosome 2 open reading frame 37	2.172	1.119	0.000	0.000
629	203021_at	SLPI	secretory leukocyte peptidase inhibitor	2.167	1.116	0.000	0.000
630	210802_s_at	DIMT1L	DIM1 dimethyladenosine transferase 1-like (S. cerevisiae)	2.167	1.115	0.000	0.000
631	201292_at	TOP2A	topoisomerase (DNA) II alpha 170kDa	2.165	1.114	0.000	0.000
632	205479_s_at	PLAU	plasminogen activator, urokinase	2.165	1.115	0.000	0.000
633	210951_x_at	RAB27A	RAB27A, member RAS oncogene family	2.164	1.114	0.001	0.000
634	204444_at	KIF11	kinesin family member 11	2.163	1.113	0.000	0.000
635	218719_s_at	GINS3	GINS complex subunit 3 (Psf3 homolog)	2.163	1.113	0.000	0.000
636	200646_s_at	NUCB1	nucleobindin 1	2.162	1.112	0.001	0.000
637	209954_x_at	SS18	synovial sarcoma translocation, chromosome 18	2.162	1.112	0.000	0.000
638	209349_at	RAD50	RAD50 homolog (S. cerevisiae)	2.161	1.112	0.000	0.000
639	200935_at	CALR	calreticulin	2.158	1.110	0.001	0.000
640	209260_at	SFN	stratifin	2.158	1.109	0.000	0.000
641	209853_s_at	PSME3	proteasome (prosome, macropain) activator subunit 3 (PA28 gamma; Ki)	2.158	1.110	0.000	0.000
642	204465_s_at	INA	internexin neuronal intermediate filament protein, alpha	2.157	1.109	0.000	0.000
643	204804_at	TRIM21	tripartite motif-containing 21	2.157	1.109	0.000	0.000
644	204908_s_at	BCL3	B-cell CLL/lymphoma 3	2.156	1.108	0.000	0.000
645	213850_s_at	SFRS2IP	splicing factor, arginine/serine-rich 2, interacting protein	2.154	1.107	0.001	0.000
646	221059_s_at	CHST6	carbohydrate (N-acetylglucosamine 6-O) sulfotransferase 6	2.154	1.107	0.000	0.000

	Probe	Symbol	Description	FCH-Scalp-LS-Normal	log2FCH-Scalp-LS-Normal	p-Scalp-LS-Normal	FDR-Scalp-LS-Normal
647	202510_s_at	TNFAIP2	tumor necrosis factor, alpha-induced protein 2	2.153	1.106	0.000	0.000
648	211833_s_at	BAX	BCL2-associated X protein	2.152	1.106	0.000	0.000
649	217725_x_at	SERBP1	SERPINE1 mRNA binding protein 1	2.150	1.104	0.000	0.000
650	209606_at	PSCDBP	pleckstrin homology, Sec7 and coiled-coil domains, binding protein	2.148	1.103	0.001	0.000
651	219555_s_at	CENPN	centromere protein N	2.148	1.103	0.001	0.000
652	205246_at	PEX13	peroxisome biogenesis factor 13	2.147	1.103	0.001	0.000
653	205595_at	DSG3	desmoglein 3 (pemphigus vulgaris antigen)	2.147	1.102	0.000	0.000
654	209376_x_at	SFRS2IP	splicing factor, arginine/serine-rich 2, interacting protein	2.147	1.102	0.001	0.000
655	215395_x_at	TRY6	trypsinogen C	2.146	1.102	0.000	0.000
656	202069_s_at	IDH3A	isocitrate dehydrogenase 3 (NAD+) alpha	2.142	1.099	0.000	0.000
657	202503_s_at	KIAA0101	KIAA0101	2.142	1.099	0.000	0.000
658	207708_at	ALOXE3	arachidonate lipoxygenase 3	2.142	1.099	0.000	0.000
659	201884_at	CEACAM5	carcinoembryonic antigen-related cell adhesion molecule 5	2.141	1.098	0.018	0.040
660	204715_at	PANX1	pannexin 1	2.140	1.097	0.000	0.000
661	215723_s_at	PLD1	phospholipase D1, phosphatidylcholine-specific	2.140	1.098	0.000	0.000
662	209520_s_at	NCBP1	nuclear cap binding protein subunit 1, 80kDa	2.139	1.097	0.000	0.000
663	213416_at	CERKL	ceramide kinase-like	2.139	1.097	0.001	0.000
664	203968_s_at	CDC6	cell division cycle 6 homolog (S. cerevisiae)	2.138	1.097	0.000	0.000
665	211796_s_at	TRBC1	T cell receptor beta constant 1	2.137	1.096	0.010	0.030
666	212229_s_at	FBXO21	F-box protein 21	2.137	1.096	0.001	0.000
667	204207_s_at	RNGTT	RNA guanylyltransferase and 5'-phosphatase	2.134	1.094	0.000	0.000
668	203740_at	MPHOSPH6	M-phase phosphoprotein 6	2.133	1.093	0.000	0.000
669	220599_s_at	CARD14	caspase recruitment domain family, member 14	2.130	1.091	0.000	0.000
670	215554_at	GPLD1	glycosylphosphatidylinositol specific phospholipase D1	2.129	1.090	0.018	0.040
671	204147_s_at	TFDP1	transcription factor Dp-1	2.125	1.087	0.000	0.000
673	201775_s_at	ATPAF1	ATP synthase mitochondrial F1 complex assembly factor 1	2.124	1.087	0.000	0.000
674	204048_s_at	PHACTR2	phosphatase and actin regulator 2	2.122	1.085	0.003	0.010
675	218562_s_at	TMEM57	transmembrane protein 57	2.121	1.084	0.000	0.000
676	204709_s_at	KIF23	kinesin family member 23	2.120	1.084	0.000	0.000
677	201048_x_at	RAB6A	RAB6A, member RAS oncogene family	2.119	1.083	0.001	0.000
678	205046_at	CENPE	centromere protein E, 312kDa	2.119	1.084	0.001	0.000
679	210367_s_at	PTGES	prostaglandin E synthase	2.118	1.083	0.000	0.000
680	207108_s_at	NIPBL	Nipped-B homolog (Drosophila)	2.116	1.081	0.002	0.000
681	201723_s_at	GALNT1	UDP-N-acetyl-alpha-D-galactosamine:polypeptide N-acetylgalactosaminyltransferase 1 (GalNAc-T1)	2.115	1.081	0.000	0.000
682	207124_s_at	GNB5	guanine nucleotide binding protein (G protein), beta 5	2.115	1.081	0.000	0.000
683	208650_s_at	CD24	CD24 molecule	2.113	1.079	0.000	0.000
684	214168_s_at	TJP1	tight junction protein 1 (zona occludens 1)	2.113	1.079	0.000	0.000
685	206628_at	SLC5A1	solute carrier family 5 (sodium/glucose cotransporter), member 1	2.111	1.078	0.002	0.000
686	221653_x_at	APOL2	apolipoprotein L, 2	2.109	1.077	0.000	0.000
687	202416_at	DNAJC7	DnaJ (Hsp40) homolog, subfamily C, member 7	2.108	1.076	0.000	0.000
688	207198_s_at	LIMS1	LIM and senescent cell antigen-like domains 1	2.108	1.076	0.000	0.000
689	209203_s_at	BICD2	bicaudal D homolog 2 (Drosophila)	2.107	1.075	0.000	0.000
690	207388_s_at	PTGES	prostaglandin E synthase	2.105	1.074	0.000	0.000
691	205538_at	CORO2A	coronin, actin binding protein, 2A	2.103	1.072	0.000	0.000
692	211193_at	TP63	tumor protein p63	2.099	1.069	0.000	0.000
693	216125_s_at	RANBP9	RAN binding protein 9	2.096	1.068	0.000	0.000
694	212577_at	SMCHD1	structural maintenance of chromosomes flexible hinge domain containing 1	2.095	1.067	0.000	0.000
695	203616_at	POLB	polymerase (DNA directed), beta	2.094	1.066	0.000	0.000
696	205282_at	LRP8	low density lipoprotein receptor-related protein 8, apolipoprotein e receptor	2.094	1.066	0.000	0.000
697	221830_at	RAP2A	RAP2A, member of RAS oncogene family	2.094	1.066	0.000	0.000
698	220655_at	TNIP3	TNFAIP3 interacting protein 3	2.092	1.065	0.011	0.030
699	202918_s_at	MOBKL3	MOB1, Mps One Binder kinase activator-like 3 (yeast)	2.089	1.063	0.000	0.000
700	219267_at	GLTP	glycolipid transfer protein	2.088	1.062	0.000	0.000
701	212142_at	MCM4	minichromosome maintenance complex component 4	2.087	1.062	0.001	0.000
702	201073_s_at	SMARCC1	SWI/SNF related, matrix associated, actin dependent regulator of chromatin, subfamily c, member 1	2.086	1.061	0.000	0.000
703	202497_x_at	SLC2A3	solute carrier family 2 (facilitated glucose transporter), member 3	2.086	1.061	0.002	0.000
704	213358_at	KIAA0802	KIAA0802	2.086	1.061	0.000	0.000
705	217785_s_at	YKT6	YKT6 v-SNARE homolog (S. cerevisiae)	2.085	1.060	0.000	0.000
706	210943_s_at	LYST	lysosomal trafficking regulator	2.084	1.059	0.017	0.040
707	210053_at	TAF5	TAF5 RNA polymerase II, TATA box binding protein (TBP)-associated factor, 100kDa	2.075	1.053	0.000	0.000
708	211804_s_at	CDK2	cyclin-dependent kinase 2	2.075	1.053	0.001	0.000
709	214119_s_at	FKBP1A	FK506 binding protein 1A, 12kDa	2.074	1.053	0.000	0.000
710	214440_at	NAT1	N-acetyltransferase 1 (arylamine N-acetyltransferase)	2.074	1.052	0.000	0.000
711	210052_s_at	TPX2	TPX2, microtubule-associated, homolog (Xenopus laevis)	2.072	1.051	0.000	0.000
712	202118_s_at	CPNE3	copine III	2.071	1.051	0.005	0.010
713	209006_s_at	C1orf63	chromosome 1 open reading frame 63	2.071	1.051	0.000	0.000
714	209642_at	BUB1	BUB1 budding uninhibited by benzimidazoles 1 homolog (yeast)	2.071	1.050	0.000	0.000
715	203199_s_at	MTRR	5-methyltetrahydrofolate-homocysteine methyltransferase reductase	2.068	1.048	0.000	0.000
716	203770_s_at	STS	steroid sulfatase (microsomal), isozyme S	2.067	1.047	0.000	0.000
717	207038_at	SLC16A6	solute carrier family 16, member 6 (monocarboxylic acid transporter 7)	2.067	1.048	0.001	0.000
718	221903_s_at	CYLD	cylindromatosis (turban tumor syndrome)	2.065	1.046	0.000	0.000
719	200987_x_at	PSME3	proteasome (prosome, macropain) activator subunit 3 (PA28 gamma; Ki)	2.064	1.045	0.000	0.000

	Probe	Symbol	Description	FCH-Scalp-LS-Normal	log2FCH-Scalp-LS-Normal	p-Scalp-LS-Normal	FDR-Scalp-LS-Normal
720	201768_s_at	CLINT1	clathrin interactor 1	2.063	1.045	0.000	0.000
721	207319_s_at	CDC2L5	cell division cycle 2-like 5 (cholinesterase-related cell division controller)	2.063	1.045	0.000	0.000
722	211300_s_at	TP53	tumor protein p53 (Li-Fraumeni syndrome)	2.061	1.043	0.007	0.020
723	212178_s_at	POM121	POM121 membrane glycoprotein (rat)	2.061	1.043	0.000	0.000
724	210935_s_at	WDR1	WD repeat domain 1	2.060	1.043	0.011	0.030
725	217196_s_at	CAMSAP1L1	calmodulin regulated spectrin-associated protein 1-like 1	2.058	1.042	0.000	0.000
726	203357_s_at	CAPN7	calpain 7	2.056	1.040	0.000	0.000
727	206472_s_at	TLE3	transducin-like enhancer of split 3 (E(sp1) homolog, Drosophila)	2.056	1.040	0.000	0.000
728	203319_s_at	ZNF148	zinc finger protein 148	2.055	1.039	0.000	0.000
729	37028_at	PPP1R15A	protein phosphatase 1, regulatory (inhibitor) subunit 15A	2.054	1.039	0.000	0.000
730	220651_s_at	MCM10	minichromosome maintenance complex component 10	2.049	1.035	0.000	0.000
731	211778_s_at	OVOL2	ovo-like 2 (Drosophila)	2.048	1.034	0.000	0.000
732	213664_at	SLC1A1	solute carrier family 1 (neuronal/epithelial high affinity glutamate transporter, system Xag), member 1	2.048	1.035	0.004	0.010
733	219688_at	BBS7	Bardet-Biedl syndrome 7	2.046	1.033	0.001	0.000
734	212840_at	UBXD7	UBX domain containing 7	2.045	1.032	0.001	0.000
735	203321_s_at	ADNP2	ADNP homeobox 2	2.042	1.030	0.000	0.000
736	220576_at	PGAP1	GPI deacylase	2.041	1.029	0.000	0.000
737	205474_at	CRLF3	cytokine receptor-like factor 3	2.040	1.029	0.000	0.000
738	200890_s_at	SSR1	signal sequence receptor, alpha (translocon-associated protein alpha)	2.039	1.028	0.000	0.000
739	219666_at	MS4A6A	membrane-spanning 4-domains, subfamily A, member 6A	2.038	1.027	0.008	0.020
740	209363_s_at	MED21	mediator complex subunit 21	2.037	1.027	0.000	0.000
741	219836_at	ZBED2	zinc finger, BED-type containing 2	2.037	1.026	0.003	0.010
742	203934_at	KDR	kinase insert domain receptor (a type III receptor tyrosine kinase)	2.035	1.025	0.000	0.000
743	202558_s_at	STCH	stress 70 protein chaperone, microsome-associated, 60kDa	2.033	1.024	0.006	0.020
744	212378_at	GART	phosphoribosylglycinamide formyltransferase, phosphoribosylglycinamide synthetase, phosphoribosylaminoimidazole synthetase	2.030	1.022	0.000	0.000
745	201695_s_at	NP	nucleoside phosphorylase	2.028	1.020	0.000	0.000
746	209231_s_at	DCTN5	dynactin 5 (p25)	2.028	1.020	0.000	0.000
747	211013_x_at	PML	promyelocytic leukemia	2.028	1.020	0.000	0.000
748	210845_s_at	PLAUR	plasminogen activator, urokinase receptor	2.026	1.019	0.014	0.030
749	202637_s_at	ICAM1	intercellular adhesion molecule 1 (CD54), human rhinovirus receptor	2.025	1.018	0.000	0.000
750	218209_s_at	P15RS	hypothetical protein FLJ10656	2.025	1.018	0.000	0.000
751	219793_at	SNX16	sorting nexin 16	2.025	1.018	0.001	0.000
752	208897_s_at	DDX18	DEAD (Asp-Glu-Ala-Asp) box polypeptide 18	2.022	1.016	0.000	0.000
753	203300_x_at	AP1S2	adaptor-related protein complex 1, sigma 2 subunit	2.021	1.015	0.002	0.000
754	219211_at	USP18	ubiquitin specific peptidase 18	2.021	1.015	0.003	0.010
755	205895_s_at	NOLC1	nucleolar and coiled-body phosphoprotein 1	2.020	1.014	0.000	0.000
756	206166_s_at	CLCA2	chloride channel, calcium activated, family member 2	2.020	1.014	0.000	0.000
757	201454_s_at	NPEPPS	aminopeptidase puromycin sensitive	2.019	1.014	0.000	0.000
758	201694_s_at	EGR1	early growth response 1	2.017	1.012	0.001	0.000
759	212937_s_at	COL6A1	collagen, type VI, alpha 1	2.015	1.010	0.007	0.020
760	214805_at	EIF4A1	eukaryotic translation initiation factor 4A, isoform 1	2.015	1.011	0.000	0.000
761	207165_at	HMMR	hyaluronan-mediated motility receptor (RHAMM)	2.014	1.010	0.003	0.010
762	209514_s_at	RAB27A	RAB27A, member RAS oncogene family	2.014	1.010	0.004	0.010
763	213193_x_at	TRBC1	T cell receptor beta constant 1	2.013	1.010	0.007	0.020
764	204049_s_at	PHACTR2	phosphatase and actin regulator 2	2.011	1.008	0.007	0.020
765	215990_s_at	BCL6	B-cell CLL/lymphoma 6 (zinc finger protein 51)	2.008	1.006	0.000	0.000
766	218802_at	CCDC109B	coiled-coil domain containing 109B	2.007	1.005	0.000	0.000
767	219934_s_at	SULT1E1	sulfotransferase family 1E, estrogen-preferring, member 1	2.006	1.004	0.017	0.040
768	200628_s_at	WARS	tryptophanyl-tRNA synthetase	2.005	1.004	0.000	0.000
769	203553_s_at	MAP4K5	mitogen-activated protein kinase kinase kinase kinase 5	2.005	1.003	0.000	0.000
770	221695_s_at	MAP3K2	mitogen-activated protein kinase kinase kinase 2	2.004	1.003	0.000	0.000
771	210972_x_at	TRA@	T cell receptor alpha locus	2.003	1.002	0.000	0.000
772	217826_s_at	UBE2J1	ubiquitin-conjugating enzyme E2, J1 (UBC6 homolog, yeast)	2.002	1.001	0.000	0.000
773	200729_s_at	ACTR2	ARP2 actin-related protein 2 homolog (yeast)	2.000	1.000	0.000	0.000
774	207387_s_at	GK	glycerol kinase	2.000	1.000	0.011	0.030

Table E. Downregulated genes in scalp psoriasis: non-lesional vs. normal.

	Probe	Symbol	Description	FCH-Scalp-NL-N	log2FCH-Scalp-NL-N	p-Scalp-NL-N	FDR-Scalp-NL-N
1	211430_s_at	IGHG3	immunoglobulin heavy constant gamma 3 (G3m marker)	0.050	-4.326	0.001	0.000
2	214677_x_at	IGLJ3	immunoglobulin lambda joining 3	0.058	-4.114	0.000	0.000
3	209138_x_at	IGL@	immunoglobulin lambda locus	0.061	-4.044	0.000	0.000
4	220507_s_at	UPB1	ureidopropionase, beta	0.104	-3.268	0.000	0.000
5	214370_at	S100A8	S100 calcium binding protein A8	0.120	-3.055	0.000	0.000
6	215946_x_at	CTA-246H3.1	similar to omega protein	0.130	-2.945	0.000	0.000
7	217022_s_at	IGHA1	immunoglobulin heavy constant alpha 1	0.132	-2.920	0.004	0.030
8	213920_at	CUTL2	cut-like 2 (Drosophila)	0.134	-2.901	0.000	0.000
9	204515_at	HSD3B1	hydroxy-delta-5-steroid dehydrogenase, 3 beta- and steroid delta-isomerase 1	0.135	-2.890	0.003	0.020
10	207367_at	ATP12A	ATPase, H+/K+ transporting, nongastric, alpha polypeptide	0.138	-2.858	0.000	0.000
11	203027_s_at	MVD	mevalonate (diphospho) decarboxylase	0.139	-2.849	0.000	0.000
12	203400_s_at	TF	transferrin	0.140	-2.841	0.004	0.020
13	217059_at	MUC7	mucin 7, secreted	0.140	-2.833	0.000	0.000
14	206392_s_at	RARRES1	retinoic acid receptor responder (tazarotene induced) 1	0.146	-2.773	0.007	0.040
15	208331_at	BPY2	basic charge, Y-linked, 2	0.146	-2.777	0.001	0.010
16	215121_x_at	IGL@	immunoglobulin lambda locus	0.149	-2.746	0.000	0.000
17	221651_x_at	IGKC	immunoglobulin kappa constant	0.155	-2.690	0.001	0.010
18	214068_at	BEAN	brain expressed, associated with Nedd4	0.161	-2.637	0.000	0.000
19	204733_at	KLK6	kallikrein-related peptidase 6	0.162	-2.628	0.001	0.000
20	207847_s_at	MUC1	mucin 1, cell surface associated	0.162	-2.627	0.000	0.000
21	221671_x_at	IGKC	immunoglobulin kappa constant	0.163	-2.613	0.002	0.010
22	221872_at	RARRES1	retinoic acid receptor responder (tazarotene induced) 1	0.163	-2.621	0.006	0.040
23	213693_s_at	MUC1	mucin 1, cell surface associated	0.167	-2.581	0.000	0.000
24	220801_s_at	HAO2	hydroxyacid oxidase 2 (long chain)	0.170	-2.555	0.004	0.020
25	204818_at	HSD17B2	hydroxysteroid (17-beta) dehydrogenase 2	0.178	-2.489	0.000	0.000
26	214669_x_at	IGKC	immunoglobulin kappa constant	0.180	-2.471	0.001	0.000
27	206391_at	RARRES1	retinoic acid receptor responder (tazarotene induced) 1	0.182	-2.455	0.001	0.010
28	215379_x_at	IGL@	immunoglobulin lambda locus	0.182	-2.454	0.001	0.000
29	219131_at	UBIAD1	UbiA prenyltransferase domain containing 1	0.193	-2.374	0.000	0.000
30	212883_at	APOE	apolipoprotein E	0.194	-2.366	0.000	0.000
31	214063_s_at	TF	transferrin	0.195	-2.358	0.002	0.010
33	214768_x_at	IGKC	immunoglobulin kappa constant	0.224	-2.159	0.000	0.000
34	219975_x_at	OLAH	oleoyl-ACP hydrolase	0.247	-2.018	0.003	0.020
35	205208_at	ALDH1L1	aldehyde dehydrogenase 1 family, member L1	0.250	-2.001	0.000	0.000
36	219874_at	SLC12A8	solute carrier family 12 (potassium/chloride transporters), member 8	0.251	-1.995	0.000	0.000
37	209696_at	FBP1	fructose-1,6-bisphosphatase 1	0.253	-1.984	0.007	0.040
38	210452_x_at	CYP4F2	cytochrome P450, family 4, subfamily F, polypeptide 2	0.253	-1.983	0.000	0.000
39	212531_at	LCN2	lipocalin 2 (oncogene 24p3)	0.261	-1.938	0.005	0.030
40	214836_x_at	IGKC	immunoglobulin kappa constant	0.261	-1.940	0.002	0.010
41	222071_s_at	SLCO4C1	solute carrier organic anion transporter family, member 4C1	0.262	-1.930	0.002	0.020
42	209173_at	AGR2	anterior gradient homolog 2 (Xenopus laevis)	0.263	-1.928	0.002	0.010
43	221142_s_at	PECR	peroxisomal trans-2-enoyl-CoA reductase	0.263	-1.929	0.006	0.040
44	207993_s_at	CHP	calcium binding protein P22	0.269	-1.893	0.000	0.000
45	203576_at	BCAT2	branched chain aminotransferase 2, mitochondrial	0.283	-1.819	0.000	0.000
46	220937_s_at	ST6GALNAC4	ST6 (alpha-N-acetyl-neuraminyl-2,3-beta-galactosyl-1,3)-N-acetylgalactosaminide alpha-2,6-sialyltransferase 4	0.283	-1.822	0.000	0.000
47	204148_s_at	POMZP3	POM (POM121 homolog, rat) and ZP3 fusion	0.284	-1.817	0.000	0.000
48	201625_s_at	INSIG1	insulin induced gene 1	0.285	-1.812	0.002	0.010
49	209398_at	HIST1H1C	histone cluster 1, H1c	0.289	-1.789	0.001	0.000
50	213490_s_at	MAP2K2	mitogen-activated protein kinase kinase 2	0.297	-1.754	0.000	0.000
51	210667_s_at	C21orf33	chromosome 21 open reading frame 33	0.300	-1.737	0.000	0.000
52	205843_x_at	CRAT	carnitine acetyltransferase	0.302	-1.728	0.002	0.010
53	213240_s_at	KRT4	keratin 4	0.306	-1.708	0.000	0.000
54	219428_s_at	PXMP4	peroxisomal membrane protein 4, 24kDa	0.308	-1.698	0.000	0.000
55	215649_s_at	MVK	mevalonate kinase (mevalonic aciduria)	0.317	-1.657	0.000	0.000
56	222025_s_at	OPLAH	5-oxoprolinase (ATP-hydrolysing)	0.317	-1.658	0.000	0.000
57	209577_at	PCYT2	phosphate cytidylyltransferase 2, ethanolamine	0.319	-1.648	0.000	0.000
58	210910_s_at	POMZP3	POM (POM121 homolog, rat) and ZP3 fusion	0.320	-1.642	0.002	0.010
59	209522_s_at	CRAT	carnitine acetyltransferase	0.321	-1.637	0.007	0.040
60	206214_at	PLA2G7	phospholipase A2, group VII (platelet-activating factor acetylhydrolase, plasma)	0.328	-1.608	0.006	0.030
61	202982_s_at	ACOT2	acyl-CoA thioesterase 2	0.330	-1.598	0.008	0.040
62	217148_x_at	IGL@	immunoglobulin lambda locus	0.332	-1.589	0.009	0.040
64	204476_s_at	PC	pyruvate carboxylase	0.337	-1.571	0.000	0.000
65	43544_at	MED16	mediator complex subunit 16	0.341	-1.552	0.000	0.000
66	207192_at	DNASE1L2	deoxyribonuclease I-like 2	0.343	-1.545	0.002	0.020
67	209608_s_at	ACAT2	acetyl-Coenzyme A acetyltransferase 2 (acetoacetyl Coenzyme A thiolase)	0.343	-1.543	0.000	0.000
68	205364_at	ACOX2	acyl-Coenzyme A oxidase 2, branched chain	0.344	-1.540	0.001	0.010
69	204607_at	HMGCS2	3-hydroxy-3-methylglutaryl-Coenzyme A synthase 2 (mitochondrial)	0.346	-1.530	0.002	0.010
70	213553_x_at	APOC1	apolipoprotein C-I	0.346	-1.530	0.000	0.000
71	206605_at	P11	26 serine protease	0.347	-1.526	0.000	0.000
72	201791_s_at	DHCR7	7-dehydrocholesterol reductase	0.353	-1.500	0.000	0.000
73	209800_at	KRT16	keratin 16 (focal non-epidermolytic palmoplantar keratoderma)	0.356	-1.492	0.006	0.030
74	220067_at	SPTBN5	spectrin, beta, non-erythrocytic 5	0.356	-1.492	0.000	0.000
75	37966_at	PARVB	parvin, beta	0.359	-1.480	0.000	0.000
76	220197_at	ATP6V0A4	ATPase, H+ transporting, lysosomal V0 subunit a4	0.360	-1.475	0.001	0.010
77	202275_at	G6PD	glucose-6-phosphate dehydrogenase	0.362	-1.465	0.000	0.000
78	209605_at	TST	thiosulfate sulfurtransferase (rhodanese)	0.362	-1.465	0.000	0.000
80	221604_s_at	PEX16	peroxisomal biogenesis factor 16	0.365	-1.453	0.000	0.000
81	209498_at	CEACAM1	carcinoembryonic antigen-related cell adhesion molecule 1 (biliary glycoprotein)	0.366	-1.452	0.000	0.000
82	212707_s_at	RASA4	RAS p21 protein activator 4	0.368	-1.443	0.000	0.000
83	210130_s_at	TM7SF2	transmembrane 7 superfamily member 2	0.370	-1.435	0.000	0.000
84	212276_at	LPIN1	lipin 1	0.370	-1.434	0.000	0.000
85	202025_x_at	ACAA1	acetyl-Coenzyme A acyltransferase 1 (peroxisomal 3-oxoacyl-Coenzyme A thiolase)	0.371	-1.431	0.000	0.000

	Probe	Symbol	Description	FCH-Scalp-NL-N	log2FCH-Scalp-NL-N	p-Scalp-NL-N	FDR-Scalp-NL-N
86	210521_s_at	FETUB	fetuin B	0.371	-1.432	0.000	0.000
87	206466_at	ACSBG1	acyl-CoA synthetase bubblegum family member 1	0.373	-1.424	0.008	0.040
88	220233_at	FBXO17	F-box protein 17	0.379	-1.399	0.001	0.000
89	200979_at	MAP3K15	mitogen-activated protein kinase kinase kinase 15	0.380	-1.394	0.000	0.000
90	209279_s_at	NSDHL	NAD(P) dependent steroid dehydrogenase-like	0.380	-1.397	0.000	0.000
91	214681_at	GK	glycerol kinase	0.382	-1.388	0.001	0.000
92	215184_at	DAPK2	death-associated protein kinase 2	0.383	-1.385	0.000	0.000
94	206869_at	CHAD	chondroadherin	0.384	-1.381	0.000	0.000
95	220751_s_at	C5orf4	chromosome 5 open reading frame 4	0.384	-1.380	0.000	0.000
96	221222_s_at	C1orf56	chromosome 1 open reading frame 56	0.385	-1.376	0.000	0.000
97	201790_s_at	DHCR7	7-dehydrocholesterol reductase	0.386	-1.373	0.000	0.000
98	206514_s_at	CYP4F3	cytochrome P450, family 4, subfamily F, polypeptide 3	0.386	-1.374	0.000	0.000
99	211663_x_at	PTGDS	prostaglandin D2 synthase 21kDa (brain)	0.386	-1.373	0.000	0.000
100	212187_x_at	PTGDS	prostaglandin D2 synthase 21kDa (brain)	0.386	-1.372	0.006	0.030
101	214001_x_at	RPS10	ribosomal protein S10	0.388	-1.367	0.001	0.010
102	215082_at	ELOVL5	ELOVL family member 5, elongation of long chain fatty acids (FEN1/Elo2, SUR4/Elo3-like, yeast)	0.388	-1.365	0.001	0.000
103	218922_s_at	LASS4	LAG1 homolog, ceramide synthase 4	0.388	-1.367	0.000	0.000
104	214549_x_at	SPRR1A	small proline-rich protein 1A	0.389	-1.364	0.000	0.000
105	222011_s_at	ACAT2	acetyl-Coenzyme A acetyltransferase 2 (acetoacetyl Coenzyme A thiolase)	0.389	-1.360	0.000	0.000
106	206723_s_at	EDG4	endothelial differentiation, lysophosphatidic acid G-protein-coupled receptor, 4	0.390	-1.360	0.000	0.000
107	210082_at	ABCA4	ATP-binding cassette, sub-family A (ABC1), member 4	0.392	-1.350	0.006	0.030
108	201275_at	FDPS	farnesyl diphosphate synthase (farnesyl pyrophosphate synthetase, dimethylallyltranstransferase, geranyltranstransferase)	0.395	-1.341	0.000	0.000
109	218476_at	POMT1	protein-O-mannosyltransferase 1	0.396	-1.337	0.000	0.000
110	220357_s_at	SGK2	serum/glucocorticoid regulated kinase 2	0.398	-1.328	0.000	0.000
111	205232_s_at	PAFAH2	platelet-activating factor acetylhydrolase 2, 40kDa	0.399	-1.325	0.000	0.000
112	221614_s_at	RPH3AL	rabphilin 3A-like (without C2 domains)	0.399	-1.326	0.000	0.000
113	211564_s_at	PDLIM4	PDZ and LIM domain 4	0.406	-1.301	0.000	0.000
114	218322_s_at	ACSL5	acyl-CoA synthetase long-chain family member 5	0.408	-1.292	0.000	0.000
115	201171_at	ATP6V0E1	ATPase, H+ transporting, lysosomal 9kDa, V0 subunit e1	0.410	-1.287	0.000	0.000
116	48030_i_at	C5orf4	chromosome 5 open reading frame 4	0.410	-1.286	0.001	0.000
117	221009_s_at	ANGPTL4	angiopoietin-like 4	0.411	-1.284	0.001	0.010
118	207254_at	SLC15A1	solute carrier family 15 (oligopeptide transporter), member 1	0.412	-1.279	0.000	0.000
119	221545_x_at	MED16	mediator complex subunit 16	0.412	-1.279	0.000	0.000
120	221810_at	RAB15	RAB15, member RAS onocogene family	0.412	-1.280	0.000	0.000
121	204067_at	SUOX	sulfite oxidase	0.413	-1.276	0.000	0.000
122	203798_s_at	VSNL1	visinin-like 1	0.414	-1.271	0.001	0.010
123	221848_at	ZGPAT	zinc finger, CCCH-type with G patch domain	0.414	-1.272	0.000	0.000
124	211695_x_at	MUC1	mucin 1, cell surface associated	0.416	-1.265	0.000	0.000
125	212281_s_at	TMEM97	transmembrane protein 97	0.416	-1.267	0.006	0.040
126	205233_s_at	PAFAH2	platelet-activating factor acetylhydrolase 2, 40kDa	0.418	-1.259	0.000	0.000
127	204687_at	DKFZP564O0823	DKFZP564O0823 protein	0.419	-1.254	0.007	0.040
128	205918_at	SLC4A3	solute carrier family 4, anion exchanger, member 3	0.420	-1.251	0.000	0.000
129	218840_s_at	NADSYN1	NAD synthetase 1	0.420	-1.250	0.000	0.000
130	205627_at	CDA	cytidine deaminase	0.421	-1.248	0.001	0.010
131	218739_at	ABHD5	abhydrolase domain containing 5	0.424	-1.237	0.000	0.000
132	205464_at	SCNN1B	sodium channel, nonvoltage-gated 1, beta (Liddle syndrome)	0.425	-1.234	0.001	0.010
133	219389_at	SUSD4	sushi domain containing 4	0.425	-1.234	0.001	0.010
134	208998_at	UCP2	uncoupling protein 2 (mitochondrial, proton carrier)	0.430	-1.217	0.000	0.000
135	218507_at	HIG2	hypoxia-inducible protein 2	0.430	-1.216	0.002	0.010
136	203189_s_at	NDUFS8	NADH dehydrogenase (ubiquinone) Fe-S protein 8, 23kDa (NADH-coenzyme Q reductase)	0.431	-1.214	0.000	0.000
137	205864_at	SLC7A4	solute carrier family 7 (cationic amino acid transporter, y+ system), member 4	0.433	-1.207	0.000	0.000
139	220675_s_at	PNPLA3	patatin-like phospholipase domain containing 3	0.434	-1.206	0.004	0.020
140	209016_s_at	KRT7	keratin 7	0.435	-1.201	0.008	0.040
141	204638_at	ACP5	acid phosphatase 5, tartrate resistant	0.437	-1.194	0.000	0.000
143	209792_s_at	KLK10	kallikrein-related peptidase 10	0.438	-1.190	0.001	0.010
145	219359_at	ATHL1	ATH1, acid trehalase-like 1 (yeast)	0.439	-1.187	0.000	0.000
146	202740_at	ACY1	aminoacylase 1	0.442	-1.178	0.000	0.000
147	218608_at	ATP13A2	ATPase type 13A2	0.442	-1.178	0.005	0.030
148	202067_s_at	LDLR	low density lipoprotein receptor (familial hypercholesterolemia)	0.443	-1.176	0.007	0.040
149	202030_at	BCKDK	branched chain ketoacid dehydrogenase kinase	0.445	-1.170	0.000	0.000
151	205221_at	HGD	homogentisate 1,2-dioxygenase (homogentisate oxidase)	0.445	-1.167	0.004	0.030
152	204669_s_at	RNF24	ring finger protein 24	0.447	-1.162	0.000	0.000
153	222155_s_at	GPR172A	G protein-coupled receptor 172A	0.447	-1.162	0.000	0.000
154	206515_at	CYP4F3	cytochrome P450, family 4, subfamily F, polypeptide 3	0.448	-1.159	0.001	0.010
155	218664_at	MECR	mitochondrial trans-2-enoyl-CoA reductase	0.448	-1.160	0.000	0.000
156	219752_at	RASAL1	RAS protein activator like 1 (GAP1 like)	0.448	-1.158	0.000	0.000
157	202003_s_at	ACAA2	acetyl-Coenzyme A acyltransferase 2 (mitochondrial 3-oxoacyl-Coenzyme A thiolase)	0.449	-1.156	0.003	0.020
158	203382_s_at	APOE	apolipoprotein E	0.449	-1.156	0.002	0.010
159	203515_s_at	PMVK	phosphomevalonate kinase	0.449	-1.154	0.000	0.000
160	203821_at	HBEGF	heparin-binding EGF-like growth factor	0.449	-1.155	0.003	0.020
163	209919_x_at	GGT1	gamma-glutamyltransferase 1	0.449	-1.155	0.000	0.000
164	219076_s_at	PXMP2	peroxisomal membrane protein 2, 22kDa	0.449	-1.155	0.001	0.000
166	211748_x_at	PTGDS	prostaglandin D2 synthase 21kDa (brain)	0.450	-1.153	0.009	0.040
167	215966_x_at	GK3P	glycerol kinase 3 pseudogene	0.451	-1.148	0.002	0.010
168	220425_x_at	ROPN1	ropporin, rhophilin associated protein 1	0.451	-1.149	0.005	0.030
169	204981_at	SLC22A18	solute carrier family 22 (organic cation transporter), member 18	0.452	-1.146	0.000	0.000
170	205031_at	EFNB3	ephrin-B3	0.453	-1.143	0.000	0.000
171	204139_x_at	MZF1	myeloid zinc finger 1	0.455	-1.136	0.000	0.000
172	217973_at	DCXR	dicarbonyl/L-xylulose reductase	0.455	-1.136	0.000	0.000
173	211056_s_at	SRD5A1	steroid-5-alpha-reductase, alpha polypeptide 1 (3-oxo-5 alpha-steroid delta 4-dehydrogenase alpha 1)	0.456	-1.133	0.003	0.020
174	202735_at	EBP	emopamil binding protein (sterol isomerase)	0.457	-1.131	0.000	0.000
175	208700_s_at	TKT	transketolase (Wernicke-Korsakoff syndrome)	0.458	-1.126	0.000	0.000
176	214041_x_at	RPL37A	ribosomal protein L37a	0.458	-1.126	0.003	0.020
177	37117_at	ARHGAP8	Rho GTPase activating protein 8	0.458	-1.127	0.002	0.020
178	206628_at	SLC5A1	solute carrier family 5 (sodium/glucose cotransporter), member 1	0.459	-1.122	0.004	0.030

	Probe	Symbol	Description	FCH-Scalp-NL-N	log2FCH-Scalp-NL-N	p-Scalp-NL-N	FDR-Scalp-NL-N
179	202525_at	PRSS8	protease, serine, 8	0.460	-1.120	0.000	0.000
180	206754_s_at	CYP2B6	cytochrome P450, family 2, subfamily B, polypeptide 6	0.460	-1.121	0.000	0.000
181	213935_at	ABHD5	abhydrolase domain containing 5	0.460	-1.121	0.002	0.020
182	214023_x_at	TUBB2B	tubulin, beta 2B	0.460	-1.120	0.000	0.000
183	218921_at	SIGIRR	single immunoglobulin and toll-interleukin 1 receptor (TIR) domain	0.460	-1.119	0.000	0.000
184	218795_at	ACP6	acid phosphatase 6, lysophosphatidic	0.461	-1.118	0.001	0.010
185	210336_x_at	MZF1	myeloid zinc finger 1	0.462	-1.115	0.000	0.000
186	45653_at	KCTD13	potassium channel tetramerisation domain containing 13	0.462	-1.115	0.006	0.030
187	203458_at	SPR	sepiapterin reductase (7,8-dihydrobiopterin:NADP+ oxidoreductase)	0.463	-1.111	0.000	0.000
188	208534_s_at	RASA4	RAS p21 protein activator 4	0.463	-1.112	0.002	0.010
189	212212_s_at	INTS1	integrator complex subunit 1	0.463	-1.110	0.000	0.000
190	38710_at	OTUB1	OTU domain, ubiquitin aldehyde binding 1	0.463	-1.109	0.002	0.020
191	215387_x_at	GPC6	glypican 6	0.464	-1.107	0.000	0.000
192	206709_x_at	GPT	glutamic-pyruvate transaminase (alanine aminotransferase)	0.465	-1.106	0.000	0.000
193	213497_at	ABTB2	ankyrin repeat and BTB (POZ) domain containing 2	0.465	-1.105	0.000	0.000
194	46142_at	TMEM112	transmembrane protein 112	0.465	-1.104	0.000	0.000
195	213796_at	SPRR1A	small proline-rich protein 1A	0.467	-1.097	0.001	0.010
196	218272_at	FLJ20699	hypothetical protein FLJ20699	0.467	-1.098	0.000	0.000
197	209514_s_at	RAB27A	RAB27A, member RAS oncogene family	0.468	-1.096	0.006	0.040
198	209617_s_at	CTNND2	catenin (cadherin-associated protein), delta 2 (neural plakophilin-related arm-repeat protein)	0.468	-1.095	0.002	0.010
199	210653_s_at	BCKDHB	branched chain keto acid dehydrogenase E1, beta polypeptide (maple syrup urine disease)	0.469	-1.093	0.005	0.030
200	220486_x_at	TMEM164	transmembrane protein 164	0.469	-1.092	0.000	0.000
201	202856_s_at	SLC16A3	solute carrier family 16, member 3 (monocarboxylic acid transporter 4)	0.470	-1.088	0.000	0.000
202	215785_s_at	CYFIP2	cytoplasmic FMR1 interacting protein 2	0.471	-1.086	0.003	0.020
203	204730_at	RIMS3	regulating synaptic membrane exocytosis 3	0.472	-1.082	0.000	0.000
204	203119_at	CCDC86	coiled-coil domain containing 86	0.473	-1.081	0.000	0.000
205	208284_x_at	GGT1	gamma-glutamyltransferase 1	0.473	-1.080	0.000	0.000
207	222057_at	NOL12	nucleolar protein 12	0.473	-1.081	0.000	0.000
208	201490_s_at	PPIF	peptidylprolyl isomerase F (cyclophilin F)	0.474	-1.078	0.008	0.040
209	211105_s_at	NFATC1	nuclear factor of activated T-cells, cytoplasmic, calcineurin-dependent 1	0.474	-1.077	0.005	0.030
210	32836_at	AGPAT1	1-acylglycerol-3-phosphate O-acyltransferase 1 (lysophosphatidic acid acyltransferase, alpha)	0.474	-1.077	0.006	0.030
211	203652_at	MAP3K11	mitogen-activated protein kinase kinase kinase 11	0.475	-1.073	0.002	0.020
212	202793_at	MBOAT5	membrane bound O-acyltransferase domain containing 5	0.477	-1.068	0.000	0.000
213	204401_at	KCNN4	potassium intermediate/small conductance calcium-activated channel, subfamily N, member 4	0.477	-1.069	0.003	0.020
214	204546_at	KIAA0513	KIAA0513	0.478	-1.066	0.000	0.000
215	218368_s_at	TNFRSF12A	tumor necrosis factor receptor superfamily, member 12A	0.478	-1.064	0.005	0.030
216	219188_s_at	MACROD1	MACRO domain containing 1	0.478	-1.064	0.001	0.010
217	209695_at	PTP4A3	protein tyrosine phosphatase type IVA, member 3	0.480	-1.058	0.000	0.000
218	220782_x_at	KLK12	kallikrein-related peptidase 12	0.480	-1.060	0.000	0.000
219	203777_s_at	RPS6KB2	ribosomal protein S6 kinase, 70kDa, polypeptide 2	0.481	-1.055	0.000	0.000
220	204875_s_at	GMDS	GDP-mannose 4,6-dehydratase	0.481	-1.055	0.000	0.000
221	35147_at	MCF2L	MCF.2 cell line derived transforming sequence-like	0.481	-1.056	0.000	0.000
222	203702_s_at	TTLL4	tubulin tyrosine ligase-like family, member 4	0.482	-1.053	0.002	0.010
223	209262_s_at	NR2F6	nuclear receptor subfamily 2, group F, member 6	0.482	-1.054	0.000	0.000
224	219689_at	SEMA3G	sema domain, immunoglobulin domain (Ig), short basic domain, secreted, (semaphorin) 3G	0.482	-1.052	0.006	0.040
225	220734_s_at	LOC727825	hypothetical protein LOC727825	0.484	-1.046	0.000	0.000
226	222356_at	TBL1Y	transducin (beta)-like 1Y-linked	0.484	-1.046	0.000	0.000
227	200789_at	ECH1	enoyl Coenzyme A hydratase 1, peroxisomal	0.485	-1.044	0.000	0.000
228	213787_s_at	TBC1D25	TBC1 domain family, member 25	0.485	-1.044	0.001	0.010
229	212694_s_at	PCCB	propionyl Coenzyme A carboxylase, beta polypeptide	0.487	-1.038	0.000	0.000
230	215243_s_at	GJB3	gap junction protein, beta 3, 31kDa	0.488	-1.036	0.000	0.000
231	215535_s_at	AGPAT1	1-acylglycerol-3-phosphate O-acyltransferase 1 (lysophosphatidic acid acyltransferase, alpha)	0.488	-1.036	0.001	0.000
232	221551_x_at	ST6GALNAC4	ST6 (alpha-N-acetyl-neuraminyl-2,3-beta-galactosyl-1,3)-N-acetylgalactosaminide alpha-2,6-sialyltransferase 4	0.488	-1.036	0.000	0.000
233	208699_x_at	TKT	transketolase (Wernicke-Korsakoff syndrome)	0.489	-1.031	0.000	0.000
234	209618_at	CTNND2	catenin (cadherin-associated protein), delta 2 (neural plakophilin-related arm-repeat protein)	0.489	-1.031	0.000	0.000
235	207081_s_at	PI4KA	phosphatidylinositol 4-kinase, catalytic, alpha	0.490	-1.030	0.000	0.000
236	213273_at	ODZ4	odz, odd Oz/ten-m homolog 4 (Drosophila)	0.490	-1.028	0.000	0.000
237	219044_at	THNSL2	threonine synthase-like 2 (S. cerevisiae)	0.490	-1.029	0.002	0.010
238	204247_s_at	CDK5	cyclin-dependent kinase 5	0.491	-1.027	0.000	0.000
239	210720_s_at	APBA2BP	amyloid beta (A4) precursor protein-binding, family A, member 2 binding protein	0.491	-1.026	0.000	0.000
240	221938_x_at	MED16	mediator complex subunit 16	0.491	-1.027	0.000	0.000
241	41160_at	MBD3	methyl-CpG binding domain protein 3	0.491	-1.026	0.000	0.000
242	204263_s_at	CPT2	carnitine palmitoyltransferase II	0.493	-1.021	0.000	0.000
243	212274_at	LPIN1	lipin 1	0.494	-1.019	0.001	0.010
244	209017_s_at	LONP1	lon peptidase 1, mitochondrial	0.495	-1.015	0.000	0.000
246	211071_s_at	MLLT11	myeloid/lymphoid or mixed-lineage leukemia (trithorax homolog, Drosophila); translocated to, 11	0.495	-1.015	0.008	0.040
247	215489_x_at	HOMER3	homer homolog 3 (Drosophila)	0.497	-1.008	0.000	0.000
248	204212_at	ACOT8	acyl-CoA thioesterase 8	0.498	-1.007	0.000	0.000
249	212611_at	DTX4	deltex 4 homolog (Drosophila)	0.498	-1.005	0.000	0.000
250	204343_at	ABCA3	ATP-binding cassette, sub-family A (ABC1), member 3	0.499	-1.003	0.000	0.000

Table F. Upregulated genes in scalp psoriasis: non-lesional vs. normal.

	Probe	Symbol	Description	FCH-Scalp-NL-N	log2FCH-Scalp-NL-N	p-Scalp-NL-N	FDR-Scalp-NL-N
1	209189_at	FOS	v-fos FBJ murine osteosarcoma viral oncogene homolog	92.959	6.539	0.000	0.000
2	216834_at	RGS1	regulator of G-protein signaling 1	7.525	2.912	0.000	0.000
3	210764_s_at	CYR61	cysteine-rich, angiogenic inducer, 61	6.626	2.728	0.000	0.000
4	216512_s_at	DCT	dopachrome tautomerase (dopachrome delta-isomerase, tyrosine-related protein 2)	5.992	2.583	0.000	0.000
5	201996_s_at	SPEN	spen homolog, transcriptional regulator (Drosophila)	5.494	2.458	0.000	0.000
6	209060_x_at	NCOA3	nuclear receptor coactivator 3	5.004	2.323	0.000	0.000
7	219915_s_at	SLC16A10	solute carrier family 16, member 10 (aromatic amino acid transporter)	4.957	2.309	0.000	0.000
8	212420_at	ELF1	E74-like factor 1 (ets domain transcription factor)	4.900	2.293	0.000	0.000
9	210943_s_at	LYST	lysosomal trafficking regulator	4.756	2.250	0.000	0.000
10	211965_at	ZFP36L1	zinc finger protein 36, C3H type-like 1	4.719	2.239	0.000	0.000
11	209257_s_at	SMC3	structural maintenance of chromosomes 3	4.689	2.229	0.000	0.000
12	201917_s_at	SLC25A36	solute carrier family 25, member 36	4.675	2.225	0.000	0.000
13	206858_s_at	HOXC6	homeobox C6	4.673	2.225	0.000	0.000
14	204863_s_at	IL6ST	interleukin 6 signal transducer (gp130, oncostatin M receptor)	4.660	2.220	0.000	0.000
15	208003_s_at	NFAT5	nuclear factor of activated T-cells 5, tonicity-responsive	4.488	2.166	0.001	0.000
16	202412_s_at	USP1	ubiquitin specific peptidase 1	4.379	2.131	0.000	0.000
17	201693_s_at	EGR1	early growth response 1	4.293	2.102	0.000	0.000
18	210317_s_at	YWHAE	tyrosine 3-monooxygenase/tryptophan 5-monooxygenase activation protein, epsilon polypeptide	4.254	2.089	0.000	0.000
19	201041_s_at	DUSP1	dual specificity phosphatase 1	4.155	2.055	0.000	0.000
20	201289_at	CYR61	cysteine-rich, angiogenic inducer, 61	4.134	2.048	0.000	0.000
21	206133_at	XAF1	XIAP associated factor-1	4.127	2.045	0.000	0.000
22	201801_s_at	SLC29A1	solute carrier family 29 (nucleoside transporters), member 1	4.031	2.011	0.000	0.000
23	206848_at	HOXA7	homeobox A7	3.976	1.991	0.000	0.000
24	220351_at	CCRL1	chemokine (C-C motif) receptor-like 1	3.974	1.991	0.001	0.000
25	214734_at	EXPH5	exophilin 5	3.973	1.990	0.000	0.000
26	202765_s_at	FBN1	fibrillin 1	3.958	1.985	0.000	0.000
27	205991_s_at	PRRX1	paired related homeobox 1	3.944	1.980	0.000	0.000
28	201918_at	SLC25A36	solute carrier family 25, member 36	3.910	1.967	0.000	0.000
29	212926_at	SMC5	structural maintenance of chromosomes 5	3.879	1.956	0.000	0.000
30	205168_at	DDR2	discoidin domain receptor family, member 2	3.869	1.952	0.000	0.000
31	205127_at	PTGS1	prostaglandin-endoperoxide synthase 1 (prostaglandin G/H synthase and cyclooxygenase)	3.790	1.922	0.000	0.000
32	202619_s_at	PLOD2	procollagen-lysine, 2-oxoglutarate 5-dioxygenase 2	3.712	1.892	0.000	0.000
33	202626_s_at	LYN	v-yes-1 Yamaguchi sarcoma viral related oncogene homolog	3.561	1.832	0.000	0.000
34	205908_s_at	OMD	osteomodulin	3.556	1.830	0.000	0.000
35	205809_s_at	WASL	Wiskott-Aldrich syndrome-like	3.542	1.825	0.000	0.000
36	205338_s_at	DCT	dopachrome tautomerase (dopachrome delta-isomerase, tyrosine-related protein 2)	3.485	1.801	0.001	0.010
37	212451_at	KIAA0256	KIAA0256 gene product	3.440	1.782	0.000	0.000
38	214680_at	NTRK2	neurotrophic tyrosine kinase, receptor, type 2	3.438	1.782	0.000	0.000
39	211352_s_at	NCOA3	nuclear receptor coactivator 3	3.368	1.752	0.000	0.000
40	213765_at	MFAP5	microfibrillar associated protein 5	3.367	1.752	0.004	0.020
41	201291_s_at	TOP2A	topoisomerase (DNA) II alpha 170kDa	3.343	1.741	0.000	0.000
42	209758_s_at	MFAP5	microfibrillar associated protein 5	3.300	1.723	0.003	0.020
43	207749_s_at	PPP2R3A	protein phosphatase 2 (formerly 2A), regulatory subunit B'', alpha	3.274	1.711	0.000	0.000
44	212229_s_at	FBXO21	F-box protein 21	3.272	1.710	0.000	0.000
45	212385_at	TCF4	transcription factor 4	3.267	1.708	0.000	0.000
46	202600_s_at	NRIP1	nuclear receptor interacting protein 1	3.252	1.702	0.003	0.020
47	212582_at	OSBPL8	oxysterol binding protein-like 8	3.205	1.680	0.000	0.000
48	215049_x_at	CD163	CD163 molecule	3.161	1.660	0.005	0.030
49	204048_s_at	PHACTR2	phosphatase and actin regulator 2	3.158	1.659	0.000	0.000
50	218006_s_at	ZNF22	zinc finger protein 22 (KOX 15)	3.152	1.656	0.000	0.000
51	215936_s_at	KIAA1033	KIAA1033	3.145	1.653	0.000	0.000
52	214352_s_at	KRAS	v-Ki-ras2 Kirsten rat sarcoma viral oncogene homolog	3.143	1.652	0.003	0.020
53	208965_s_at	PYHIN1	pyrin and HIN domain family, member 1	3.133	1.648	0.000	0.000
54	203767_s_at	STS	steroid sulfatase (microsomal), isozyme S	3.132	1.647	0.000	0.000
55	207177_at	PTGFR	prostaglandin F receptor (FP)	3.114	1.639	0.000	0.000
56	206061_s_at	DICER1	Dicer1, Dcr-1 homolog (Drosophila)	3.065	1.616	0.000	0.000
57	213150_at	HOXA10	homeobox A10	3.052	1.610	0.002	0.010
58	219679_s_at	WAC	WW domain containing adaptor with coiled-coil	3.051	1.609	0.001	0.010
59	202975_s_at	RHOBTB3	Rho-related BTB domain containing 3	3.020	1.595	0.000	0.000
60	209905_at	HOXA9	homeobox A9	3.017	1.593	0.003	0.020
61	212634_at	KIAA0776	KIAA0776	2.993	1.581	0.000	0.000
62	220783_at	MMP27	matrix metallopeptidase 27	2.972	1.572	0.005	0.030
63	218967_s_at	PTER	phosphotriesterase related	2.955	1.563	0.002	0.010
64	200607_s_at	RAD21	RAD21 homolog (S. pombe)	2.947	1.559	0.000	0.000
65	212840_at	UBXD7	UBX domain containing 7	2.942	1.557	0.000	0.000
66	215446_s_at	LOX	lysyl oxidase	2.941	1.556	0.000	0.000
67	212332_at	RBL2	retinoblastoma-like 2 (p130)	2.939	1.555	0.000	0.000
68	205308_at	C8orf70	chromosome 8 open reading frame 70	2.926	1.549	0.000	0.000
69	204049_s_at	PHACTR2	phosphatase and actin regulator 2	2.920	1.546	0.000	0.000
70	212587_s_at	PTPRC	protein tyrosine phosphatase, receptor type, C	2.911	1.541	0.008	0.040
71	221753_at	SSH1	slingshot homolog 1 (Drosophila)	2.906	1.539	0.000	0.000
72	218232_at	C1QA	complement component 1, q subcomponent, A chain	2.902	1.537	0.004	0.030
73	201070_x_at	SF3B1	splicing factor 3b, subunit 1, 155kDa	2.881	1.527	0.002	0.020

	Probe	Symbol	Description	FCH-Scalp-NL-N	log2FCH-Scalp-NL-N	p-Scalp-NL-N	FDR-Scalp-NL-N
74	212486_s_at	FYN	FYN oncogene related to SRC, FGR, YES	2.875	1.524	0.002	0.010
75	212678_at	NF1	neurofibromin 1 (neurofibromatosis, von Recklinghausen disease, Watson disease)	2.842	1.507	0.000	0.000
76	201149_s_at	TIMP3	TIMP metallopeptidase inhibitor 3 (Sorsby fundus dystrophy, pseudoinflammatory)	2.836	1.504	0.003	0.020
77	213940_s_at	FNBP1	formin binding protein 1	2.835	1.503	0.000	0.000
78	220576_at	PGAP1	GPI deacylase	2.835	1.503	0.000	0.000
79	201508_at	IGFBP4	insulin-like growth factor binding protein 4	2.825	1.498	0.000	0.000
80	201151_s_at	MBNL1	muscleblind-like (Drosophila)	2.820	1.496	0.000	0.000
81	220342_x_at	EDEM3	ER degradation enhancer, mannosidase alpha-like 3	2.820	1.496	0.001	0.010
82	214012_at	ARTS-1	type 1 tumor necrosis factor receptor shedding aminopeptidase regulator	2.819	1.495	0.005	0.030
83	212196_at	IL6ST	interleukin 6 signal transducer (gp130, oncostatin M receptor)	2.799	1.485	0.000	0.000
84	209894_at	LEPR	leptin receptor	2.797	1.484	0.002	0.010
85	207302_at	SGCG	sarcoglycan, gamma (35kDa dystrophin-associated glycoprotein)	2.789	1.480	0.000	0.000
86	216033_s_at	FYN	FYN oncogene related to SRC, FGR, YES	2.776	1.473	0.000	0.000
87	205357_s_at	AGTR1	angiotensin II receptor, type 1	2.772	1.471	0.003	0.020
88	203153_at	IFIT1	interferon-induced protein with tetratricopeptide repeats 1	2.767	1.468	0.005	0.030
89	212286_at	ANKRD12	ankyrin repeat domain 12	2.761	1.465	0.001	0.010
90	205842_s_at	JAK2	Janus kinase 2 (a protein tyrosine kinase)	2.759	1.464	0.000	0.000
91	213764_s_at	MFAP5	microfibrillar associated protein 5	2.734	1.451	0.008	0.040
92	201295_s_at	WSB1	WD repeat and SOCS box-containing 1	2.722	1.445	0.002	0.020
94	201294_s_at	WSB1	WD repeat and SOCS box-containing 1	2.712	1.439	0.000	0.000
95	203321_s_at	ADNP2	ADNP homeobox 2	2.692	1.428	0.000	0.000
96	213850_s_at	SFRS2IP	splicing factor, arginine/serine-rich 2, interacting protein	2.688	1.427	0.000	0.000
97	212794_s_at	KIAA1033	KIAA1033	2.683	1.424	0.000	0.000
98	206170_at	ADRB2	adrenergic, beta-2-, receptor, surface	2.681	1.423	0.000	0.000
99	211726_s_at	FMO2	flavin containing monooxygenase 2 (non-functional)	2.666	1.415	0.008	0.040
100	201085_s_at	SON	SON DNA binding protein	2.641	1.401	0.000	0.000
101	209376_x_at	SFRS2IP	splicing factor, arginine/serine-rich 2, interacting protein	2.632	1.396	0.000	0.000
102	221765_at	UGCG	UDP-glucose ceramide glucosyltransferase	2.617	1.388	0.004	0.020
103	209281_s_at	ATP2B1	ATPase, Ca++ transporting, plasma membrane 1	2.616	1.387	0.000	0.000
104	205794_s_at	NOVA1	neuro-oncological ventral antigen 1	2.615	1.387	0.001	0.010
105	203319_s_at	ZNF148	zinc finger protein 148	2.608	1.383	0.000	0.000
106	215322_at	LONRF1	LON peptidase N-terminal domain and ring finger 1	2.598	1.378	0.008	0.040
107	215177_s_at	ITGA6	integrin, alpha 6	2.585	1.370	0.002	0.010
108	214577_at	MAP1B	microtubule-associated protein 1B	2.584	1.370	0.000	0.000
109	201986_at	MED13	mediator complex subunit 13	2.574	1.364	0.000	0.000
110	211958_at	IGFBP5	insulin-like growth factor binding protein 5	2.573	1.363	0.002	0.010
111	206030_at	ASPA	aspartoacylase (Canavan disease)	2.571	1.362	0.000	0.000
112	210186_s_at	FKBP1A	FK506 binding protein 1A, 12kDa	2.566	1.360	0.000	0.000
113	207700_s_at	NCOA3	nuclear receptor coactivator 3	2.564	1.358	0.000	0.000
114	204422_s_at	FGF2	fibroblast growth factor 2 (basic)	2.532	1.340	0.001	0.010
115	210754_s_at	LYN	v-yes-1 Yamaguchi sarcoma viral related oncogene homolog	2.530	1.339	0.000	0.000
116	208016_s_at	AGTR1	angiotensin II receptor, type 1	2.529	1.339	0.000	0.000
117	208131_s_at	PTGIS	prostaglandin I2 (prostacyclin) synthase	2.515	1.331	0.002	0.010
118	201299_s_at	MOBKL1B	MOB1, Mps One Binder kinase activator-like 1B (yeast)	2.485	1.313	0.006	0.030
119	218959_at	HOXC10	homeobox C10	2.485	1.313	0.002	0.010
120	202940_at	WNK1	WNK lysine deficient protein kinase 1	2.482	1.311	0.001	0.010
121	217966_s_at	FAM129A	family with sequence similarity 129, member A	2.481	1.311	0.002	0.010
122	209763_at	CHRDL1	chordin-like 1	2.468	1.303	0.001	0.010
123	211478_s_at	DPP4	dipeptidyl-peptidase 4 (CD26, adenosine deaminase complexing protein 2)	2.468	1.303	0.005	0.030
124	202543_s_at	GMFB	glia maturation factor, beta	2.467	1.303	0.004	0.030
125	217504_at	ABCA6	ATP-binding cassette, sub-family A (ABC1), member 6	2.465	1.302	0.008	0.040
126	201844_s_at	RYBP	RING1 and YY1 binding protein	2.462	1.300	0.000	0.000
127	217862_at	PIAS1	protein inhibitor of activated STAT, 1	2.457	1.297	0.000	0.000
128	221942_s_at	GUCY1A3	guanylate cyclase 1, soluble, alpha 3	2.453	1.295	0.001	0.010
129	202124_s_at	TRAK2	trafficking protein, kinesin binding 2	2.449	1.292	0.001	0.000
130	214651_s_at	HOXA9	homeobox A9	2.449	1.292	0.004	0.020
131	204964_s_at	SSPN	sarcospan (Kras oncogene-associated gene)	2.448	1.291	0.001	0.010
132	214305_s_at	SF3B1	splicing factor 3b, subunit 1, 155kDa	2.446	1.290	0.000	0.000
133	209543_s_at	CD34	CD34 molecule	2.438	1.286	0.001	0.010
134	219757_s_at	C14orf101	chromosome 14 open reading frame 101	2.437	1.285	0.000	0.000
135	204258_at	CHD1	chromodomain helicase DNA binding protein 1	2.435	1.284	0.000	0.000
136	214697_s_at	ROD1	ROD1 regulator of differentiation 1 (S. pombe)	2.434	1.284	0.002	0.010
137	91816_f_at	MEX3D	mex-3 homolog D (C. elegans)	2.431	1.281	0.004	0.030
139	205070_at	ING3	inhibitor of growth family, member 3	2.430	1.281	0.000	0.000
140	214691_x_at	FAM63B	family with sequence similarity 63, member B	2.429	1.280	0.000	0.000
141	208328_s_at	MEF2A	myocyte enhancer factor 2A	2.428	1.280	0.000	0.000
143	213229_at	DICER1	Dicer1, Dcr-1 homolog (Drosophila)	2.427	1.279	0.000	0.000
145	202118_s_at	CPNE3	copine III	2.423	1.277	0.003	0.020
146	207382_at	TP63	tumor protein p63	2.423	1.277	0.000	0.000
147	210875_s_at	ZEB1	zinc finger E-box binding homeobox 1	2.412	1.270	0.002	0.010
148	213899_at	METAP2	methionyl aminopeptidase 2	2.397	1.261	0.000	0.000
149	200922_at	KDELR1	KDEL (Lys-Asp-Glu-Leu) endoplasmic reticulum protein retention receptor 1	2.396	1.261	0.001	0.010
151	204463_s_at	EDNRA	endothelin receptor type A	2.370	1.245	0.000	0.000
152	214499_s_at	BCLAF1	BCL2-associated transcription factor 1	2.362	1.240	0.001	0.010
153	205534_at	PCDH7	protocadherin 7	2.355	1.235	0.000	0.000
154	215228_at	NHLH2	nescient helix loop helix 2	2.355	1.235	0.005	0.030
155	210385_s_at	ARTS-1	type 1 tumor necrosis factor receptor shedding aminopeptidase regulator	2.334	1.223	0.000	0.000

	Probe	Symbol	Description	FCH-Scalp-NL-N	log2FCH-Scalp-NL-N	p-Scalp-NL-N	FDR-Scalp-NL-N
156	219625_s_at	COL4A3BP	collagen, type IV, alpha 3 (Goodpasture antigen) binding protein	2.332	1.222	0.000	0.000
157	203215_s_at	MYO6	myosin VI	2.330	1.220	0.002	0.010
158	209895_at	PTPN11	protein tyrosine phosphatase, non-receptor type 11 (Noonan syndrome 1)	2.329	1.220	0.005	0.030
159	212057_at	KIAA0182	KIAA0182	2.329	1.220	0.000	0.000
160	214119_s_at	FKBP1A	FK506 binding protein 1A, 12kDa	2.325	1.217	0.000	0.000
163	209072_at	MBP	myelin basic protein	2.314	1.210	0.000	0.000
164	213517_at	PCBP2	poly(rC) binding protein 2	2.302	1.203	0.002	0.010
166	203300_x_at	AP1S2	adaptor-related protein complex 1, sigma 2 subunit	2.299	1.201	0.001	0.010
167	204285_s_at	PMAIP1	phorbol-12-myristate-13-acetate-induced protein 1	2.299	1.201	0.002	0.010
168	221895_at	MOSPD2	motile sperm domain containing 2	2.299	1.201	0.000	0.000
169	203504_s_at	ABCA1	ATP-binding cassette, sub-family A (ABC1), member 1	2.298	1.201	0.000	0.000
170	202363_at	SPOCK1	sparc/osteonectin, cwcv and kazal-like domains proteoglycan (testican) 1	2.295	1.198	0.004	0.030
171	203523_at	LSP1	lymphocyte-specific protein 1	2.291	1.196	0.003	0.020
172	203542_s_at	KLF9	Kruppel-like factor 9	2.280	1.189	0.002	0.010
173	209969_s_at	STAT1	signal transducer and activator of transcription 1, 91kDa	2.279	1.189	0.006	0.040
174	219372_at	IFT81	intraflagellar transport 81 homolog (Chlamydomonas)	2.272	1.184	0.000	0.000
175	219237_s_at	DNAJB14	DnaJ (Hsp40) homolog, subfamily B, member 14	2.268	1.181	0.000	0.000
176	207198_s_at	LIMS1	LIM and senescent cell antigen-like domains 1	2.258	1.175	0.000	0.000
177	213435_at	SATB2	SATB homeobox 2	2.256	1.174	0.002	0.010
178	209199_s_at	MEF2C	myocyte enhancer factor 2C	2.253	1.172	0.004	0.030
179	216252_x_at	FAS	Fas (TNF receptor superfamily, member 6)	2.252	1.171	0.001	0.000
180	218273_s_at	PPM2C	protein phosphatase 2C, magnesium-dependent, catalytic subunit	2.252	1.171	0.000	0.000
181	205062_x_at	ARID4A	AT rich interactive domain 4A (RBP1-like)	2.250	1.170	0.000	0.000
182	203934_at	KDR	kinase insert domain receptor (a type III receptor tyrosine kinase)	2.245	1.167	0.000	0.000
183	211537_x_at	MAP3K7	mitogen-activated protein kinase kinase kinase 7	2.243	1.166	0.001	0.000
184	208022_s_at	CDC14B	CDC14 cell division cycle 14 homolog B (S. cerevisiae)	2.241	1.164	0.000	0.000
185	202827_s_at	MMP14	matrix metallopeptidase 14 (membrane-inserted)	2.235	1.160	0.003	0.020
186	219901_at	FGD6	FYVE, RhoGEF and PH domain containing 6	2.232	1.159	0.003	0.020
187	210053_at	TAF5	TAF5 RNA polymerase II, TATA box binding protein (TBP)-associated factor, 100kDa	2.224	1.153	0.000	0.000
188	214684_at	MEF2A	myocyte enhancer factor 2A	2.218	1.150	0.001	0.010
189	205528_s_at	RUNX1T1	runt-related transcription factor 1; translocated to, 1 (cyclin D-related)	2.209	1.144	0.001	0.000
190	209865_at	SLC35A3	solute carrier family 35 (UDP-N-acetylglucosamine (UDP-GlcNAc) transporter), member A3	2.206	1.141	0.001	0.010
191	212002_at	C1orf144	chromosome 1 open reading frame 144	2.203	1.139	0.000	0.000
192	219778_at	ZFPM2	zinc finger protein, multitype 2	2.194	1.133	0.002	0.010
193	203810_at	DNAJB4	DnaJ (Hsp40) homolog, subfamily B, member 4	2.192	1.132	0.000	0.000
194	202620_s_at	PLOD2	procollagen-lysine, 2-oxoglutarate 5-dioxygenase 2	2.191	1.132	0.002	0.010
195	219737_s_at	PCDH9	protocadherin 9	2.191	1.132	0.001	0.010
196	221830_at	RAP2A	RAP2A, member of RAS oncogene family	2.189	1.130	0.000	0.000
197	209406_at	BAG2	BCL2-associated athanogene 2	2.181	1.125	0.005	0.030
198	212188_at	KCTD12	potassium channel tetramerisation domain containing 12	2.181	1.125	0.000	0.000
199	209242_at	PEG3	paternally expressed 3	2.178	1.123	0.005	0.030
200	212290_at	SLC7A1	solute carrier family 7 (cationic amino acid transporter, y+ system), member 1	2.173	1.120	0.002	0.010
201	215719_x_at	FAS	Fas (TNF receptor superfamily, member 6)	2.168	1.116	0.007	0.040
202	204057_at	IRF8	interferon regulatory factor 8	2.167	1.116	0.005	0.030
203	207992_s_at	AMPD3	adenosine monophosphate deaminase (isoform E)	2.167	1.116	0.000	0.000
204	218458_at	GMCL1	germ cell-less homolog 1 (Drosophila)	2.163	1.113	0.007	0.040
205	219230_at	TMEM100	transmembrane protein 100	2.162	1.113	0.002	0.020
207	214869_x_at	GAPVD1	GTPase activating protein and VPS9 domains 1	2.148	1.103	0.006	0.030
208	221760_at	MAN1A1	mannosidase, alpha, class 1A, member 1	2.147	1.102	0.000	0.000
209	221950_at	EMX2	empty spiracles homeobox 2	2.145	1.101	0.002	0.010
210	213707_s_at	DLX5	distal-less homeobox 5	2.143	1.100	0.001	0.000
211	203883_s_at	RAB11FIP2	RAB11 family interacting protein 2 (class I)	2.141	1.099	0.000	0.000
212	215990_s_at	BCL6	B-cell CLL/lymphoma 6 (zinc finger protein 51)	2.140	1.097	0.000	0.000
213	205079_s_at	MPDZ	multiple PDZ domain protein	2.139	1.097	0.002	0.010
214	219326_s_at	B3GNT2	UDP-GlcNAc:betaGal beta-1,3-N-acetylglucosaminyltransferase 2	2.137	1.096	0.003	0.020
215	204969_s_at	RDX	radixin	2.136	1.095	0.001	0.010
216	209318_x_at	PLAGL1	pleiomorphic adenoma gene-like 1	2.133	1.093	0.000	0.000
217	200646_s_at	NUCB1	nucleobindin 1	2.132	1.092	0.004	0.030
218	204614_at	SERPINB2	serpin peptidase inhibitor, clade B (ovalbumin), member 2	2.128	1.090	0.000	0.000
219	201730_s_at	TPR	translocated promoter region (to activated MET oncogene)	2.126	1.088	0.001	0.000
220	219158_s_at	NARG1	NMDA receptor regulated 1	2.122	1.086	0.002	0.020
221	215039_at	LOC339524	hypothetical protein LOC339524	2.121	1.085	0.009	0.040
222	202191_s_at	GAS7	growth arrest-specific 7	2.117	1.082	0.001	0.010
223	204749_at	NAP1L3	nucleosome assembly protein 1-like 3	2.116	1.081	0.005	0.030
224	205467_at	CASP10	caspase 10, apoptosis-related cysteine peptidase	2.111	1.078	0.000	0.000
225	206710_s_at	EPB41L3	erythrocyte membrane protein band 4.1-like 3	2.104	1.073	0.003	0.020
226	202137_s_at	ZMYND11	zinc finger, MYND domain containing 11	2.101	1.071	0.001	0.000
227	208047_s_at	NAB1	NGFI-A binding protein 1 (EGR1 binding protein 1)	2.101	1.071	0.008	0.040
228	211622_s_at	ARF3	ADP-ribosylation factor 3	2.096	1.068	0.004	0.020
229	212208_at	MED13L	mediator complex subunit 13-like	2.096	1.068	0.000	0.000
230	212382_at	TCF4	transcription factor 4	2.095	1.067	0.001	0.010
231	203354_s_at	PSD3	pleckstrin and Sec7 domain containing 3	2.094	1.066	0.004	0.020
232	204944_at	PTPRG	protein tyrosine phosphatase, receptor type, G	2.091	1.064	0.009	0.040
233	215221_at	FOXP1	forkhead box P1	2.089	1.063	0.001	0.000
234	219427_at	FAT4	FAT tumor suppressor homolog 4 (Drosophila)	2.086	1.061	0.002	0.020
235	211139_s_at	NAB1	NGFI-A binding protein 1 (EGR1 binding protein 1)	2.084	1.059	0.007	0.040
236	219865_at	HSPC157	HSPC157 protein	2.082	1.058	0.000	0.000

	Probe	Symbol	Description	FCH-Scalp-NL-N	log2FCH-Scalp-NL-N	p-Scalp-NL-N	FDR-Scalp-NL-N
237	205651_x_at	RAPGEF4	Rap guanine nucleotide exchange factor (GEF) 4	2.080	1.057	0.001	0.010
238	215435_at	PDS5A	PDS5, regulator of cohesion maintenance, homolog A (S. cerevisiae)	2.080	1.057	0.001	0.010
239	202971_s_at	DYRK2	dual-specificity tyrosine-(Y)-phosphorylation regulated kinase 2	2.079	1.056	0.000	0.000
240	200935_at	CALR	calreticulin	2.068	1.048	0.004	0.020
241	204732_s_at	TRIM23	tripartite motif-containing 23	2.067	1.048	0.001	0.010
242	205170_at	STAT2	signal transducer and activator of transcription 2, 113kDa	2.067	1.047	0.006	0.030
243	219717_at	C4orf30	chromosome 4 open reading frame 30	2.067	1.047	0.008	0.040
244	214720_x_at	SEPT10	septin 10	2.066	1.047	0.001	0.010
246	214850_at	GUSBP1	glucuronidase, beta pseudogene 1	2.063	1.044	0.005	0.030
247	208994_s_at	PPIG	peptidylprolyl isomerase G (cyclophilin G)	2.061	1.043	0.001	0.000
248	204313_s_at	CREB1	cAMP responsive element binding protein 1	2.056	1.040	0.000	0.000
249	204591_at	CHL1	cell adhesion molecule with homology to L1CAM (close homolog of L1)	2.055	1.039	0.006	0.030
250	206421_s_at	SERPINB7	serpin peptidase inhibitor, clade B (ovalbumin), member 7	2.054	1.038	0.002	0.010
251	218947_s_at	PAPD1	PAP associated domain containing 1	2.053	1.038	0.000	0.000
252	210649_s_at	ARID1A	AT rich interactive domain 1A (SWI-like)	2.051	1.036	0.000	0.000
253	211536_x_at	MAP3K7	mitogen-activated protein kinase kinase kinase 7	2.046	1.033	0.001	0.010
254	204348_s_at	AK3L1	adenylate kinase 3-like 1	2.040	1.029	0.000	0.000
255	201846_s_at	RYBP	RING1 and YY1 binding protein	2.039	1.028	0.000	0.000
257	209024_s_at	SYNCRIP	synaptotagmin binding, cytoplasmic RNA interacting protein	2.034	1.024	0.000	0.000
258	208790_s_at	PTRF	polymerase I and transcript release factor	2.029	1.021	0.001	0.010
259	203603_s_at	ZEB2	zinc finger E-box binding homeobox 2	2.028	1.020	0.005	0.030
260	202902_s_at	CTSS	cathepsin S	2.027	1.020	0.002	0.020
265	209655_s_at	TMEM47	transmembrane protein 47	2.017	1.012	0.002	0.010
266	205967_at	HIST1H4C	histone cluster 1, H4c	2.015	1.011	0.003	0.020
267	209821_at	IL33	interleukin 33	2.015	1.011	0.000	0.000
268	203620_s_at	FCHSD2	FCH and double SH3 domains 2	2.014	1.010	0.000	0.000
269	204612_at	PKIA	protein kinase (cAMP-dependent, catalytic) inhibitor alpha	2.014	1.010	0.000	0.000
270	205529_s_at	RUNX1T1	runt-related transcription factor 1; translocated to, 1 (cyclin D-related)	2.014	1.010	0.005	0.030
271	218885_s_at	GALNT12	UDP-N-acetyl-alpha-D-galactosamine:polypeptide N-acetylgalactosaminyltransferase 12 (GalNAc-T12)	2.014	1.010	0.004	0.030
272	213125_at	OLFML2B	olfactomedin-like 2B	2.010	1.007	0.003	0.020
273	212951_at	GPR116	G protein-coupled receptor 116	2.008	1.005	0.001	0.010
274	215338_s_at	NKTR	natural killer-tumor recognition sequence	2.006	1.005	0.000	0.000
275	39313_at	WNK1	WNK lysine deficient protein kinase 1	2.002	1.001	0.006	0.030
276	205836_s_at	YTHDC2	YTH domain containing 2	2.001	1.001	0.000	0.000
277	210664_s_at	TFPI	tissue factor pathway inhibitor (lipoprotein-associated coagulation inhibitor)	2.001	1.001	0.006	0.030
278	213895_at	EMP1	epithelial membrane protein 1	2.001	1.001	0.003	0.020

Table G. Downregulated genes in scalp skin: non-lesional vs. normal.

	Probe	Symbol	Description	FCH-Scalp-NL-N	log2FCH-Scalp-NL-N	p-Scalp-NL-N	FDR-Scalp-NL-N
1	211430_s_at	IGHG3	immunoglobulin heavy constant gamma 3 (G3m marker)	0.050	-4.326	0.001	0.000
2	214677_x_at	IGLJ3	immunoglobulin lambda joining 3	0.058	-4.114	0.000	0.000
3	209138_x_at	IGL@	immunoglobulin lambda locus	0.061	-4.044	0.000	0.000
4	220507_s_at	UPB1	ureidopropionase, beta	0.104	-3.268	0.000	0.000
5	214370_at	S100A8	S100 calcium binding protein A8	0.120	-3.055	0.000	0.000
6	215946_x_at	CTA-246H3.1	similar to omega protein	0.130	-2.945	0.000	0.000
7	217022_s_at	IGHA1	immunoglobulin heavy constant alpha 1	0.132	-2.920	0.004	0.030
8	213920_at	CUTL2	cut-like 2 (Drosophila)	0.134	-2.901	0.000	0.000
9	204515_at	HSD3B1	hydroxy-delta-5-steroid dehydrogenase, 3 beta- and steroid delta-isomerase 1	0.135	-2.890	0.003	0.020
10	207367_at	ATP12A	ATPase, H+/K+ transporting, nongastric, alpha polypeptide	0.138	-2.858	0.000	0.000
11	203027_s_at	MVD	mevalonate (diphospho) decarboxylase	0.139	-2.849	0.000	0.000
12	203400_s_at	TF	transferrin	0.140	-2.841	0.004	0.020
13	217059_at	MUC7	mucin 7, secreted	0.140	-2.833	0.000	0.000
14	206392_s_at	RARRES1	retinoic acid receptor responder (tazarotene induced) 1	0.146	-2.773	0.007	0.040
15	208331_at	BPY2	basic charge, Y-linked, 2	0.146	-2.777	0.001	0.010
16	215121_x_at	IGL@	immunoglobulin lambda locus	0.149	-2.746	0.000	0.000
17	221651_x_at	IGKC	immunoglobulin kappa constant	0.155	-2.690	0.001	0.010
18	214068_at	BEAN	brain expressed, associated with Nedd4	0.161	-2.637	0.000	0.000
19	204733_at	KLK6	kallikrein-related peptidase 6	0.162	-2.628	0.001	0.000
20	207847_s_at	MUC1	mucin 1, cell surface associated	0.162	-2.627	0.000	0.000
21	221671_x_at	IGKC	immunoglobulin kappa constant	0.163	-2.613	0.002	0.010
22	221872_at	RARRES1	retinoic acid receptor responder (tazarotene induced) 1	0.163	-2.621	0.006	0.040
23	213693_s_at	MUC1	mucin 1, cell surface associated	0.167	-2.581	0.000	0.000
24	220801_s_at	HAO2	hydroxyacid oxidase 2 (long chain)	0.170	-2.555	0.004	0.020
25	204818_at	HSD17B2	hydroxysteroid (17-beta) dehydrogenase 2	0.178	-2.489	0.000	0.000
26	214669_x_at	IGKC	immunoglobulin kappa constant	0.180	-2.471	0.001	0.000
27	206391_at	RARRES1	retinoic acid receptor responder (tazarotene induced) 1	0.182	-2.455	0.001	0.010
28	215379_x_at	IGL@	immunoglobulin lambda locus	0.182	-2.454	0.001	0.000
29	219131_at	UBIAD1	UbiA prenyltransferase domain containing 1	0.193	-2.374	0.000	0.000
30	212883_at	APOE	apolipoprotein E	0.194	-2.366	0.000	0.000
31	214063_s_at	TF	transferrin	0.195	-2.358	0.002	0.010
33	214768_x_at	IGKC	immunoglobulin kappa constant	0.224	-2.159	0.000	0.000
34	219975_x_at	OLAH	oleoyl-ACP hydrolase	0.247	-2.018	0.003	0.020
35	205208_at	ALDH1L1	aldehyde dehydrogenase 1 family, member L1	0.250	-2.001	0.000	0.000
36	219874_at	SLC12A8	solute carrier family 12 (potassium/chloride transporters), member 8	0.251	-1.995	0.000	0.000
37	209696_at	FBP1	fructose-1,6-bisphosphatase 1	0.253	-1.984	0.007	0.040
38	210452_x_at	CYP4F2	cytochrome P450, family 4, subfamily F, polypeptide 2	0.253	-1.983	0.000	0.000
39	212531_at	LCN2	lipocalin 2 (oncogene 24p3)	0.261	-1.938	0.005	0.030
40	214836_x_at	IGKC	immunoglobulin kappa constant	0.261	-1.940	0.002	0.010
41	222071_s_at	SLCO4C1	solute carrier organic anion transporter family, member 4C1	0.262	-1.930	0.002	0.020
42	209173_at	AGR2	anterior gradient homolog 2 (Xenopus laevis)	0.263	-1.928	0.002	0.010
43	221142_s_at	PECR	peroxisomal trans-2-enoyl-CoA reductase	0.263	-1.929	0.006	0.040
44	207993_s_at	CHP	calcium binding protein P22	0.269	-1.893	0.000	0.000
45	203576_at	BCAT2	branched chain aminotransferase 2, mitochondrial	0.283	-1.819	0.000	0.000
46	220937_s_at	ST6GALNAC4	ST6 (alpha-N-acetyl-neuraminyl-2,3-beta-galactosyl-1,3)-N-acetylgalactosaminide alpha-2,6-sialyltransferase 4	0.283	-1.822	0.000	0.000
47	204148_s_at	POMZP3	POM (POM121 homolog, rat) and ZP3 fusion	0.284	-1.817	0.000	0.000
48	201625_s_at	INSIG1	insulin induced gene 1	0.285	-1.812	0.002	0.010
49	209398_at	HIST1H1C	histone cluster 1, H1c	0.289	-1.789	0.001	0.000
50	213490_s_at	MAP2K2	mitogen-activated protein kinase kinase 2	0.297	-1.754	0.000	0.000
51	210667_s_at	C21orf33	chromosome 21 open reading frame 33	0.300	-1.737	0.000	0.000
52	205843_x_at	CRAT	carnitine acetyltransferase	0.302	-1.728	0.002	0.010
53	213240_s_at	KRT4	keratin 4	0.306	-1.708	0.000	0.000
54	219428_s_at	PXMP4	peroxisomal membrane protein 4, 24kDa	0.308	-1.698	0.000	0.000
55	215649_s_at	MVK	mevalonate kinase (mevalonic aciduria)	0.317	-1.657	0.000	0.000
56	222025_s_at	OPLAH	5-oxoprolinase (ATP-hydrolysing)	0.317	-1.658	0.000	0.000
57	209577_at	PCYT2	phosphate cytidylyltransferase 2, ethanolamine	0.319	-1.648	0.000	0.000
58	210910_s_at	POMZP3	POM (POM121 homolog, rat) and ZP3 fusion	0.320	-1.642	0.002	0.010
59	209522_s_at	CRAT	carnitine acetyltransferase	0.321	-1.637	0.007	0.040
60	206214_at	PLA2G7	phospholipase A2, group VII (platelet-activating factor acetylhydrolase, plasma)	0.328	-1.608	0.006	0.030
61	202982_s_at	ACOT2	acyl-CoA thioesterase 2	0.330	-1.598	0.008	0.040
62	217148_x_at	IGL@	immunoglobulin lambda locus	0.332	-1.589	0.009	0.040
64	204476_s_at	PC	pyruvate carboxylase	0.337	-1.571	0.000	0.000
65	43544_at	MED16	mediator complex subunit 16	0.341	-1.552	0.000	0.000
66	207192_at	DNASE1L2	deoxyribonuclease I-like 2	0.343	-1.545	0.002	0.020
67	209608_s_at	ACAT2	acetyl-Coenzyme A acetyltransferase 2 (acetoacetyl Coenzyme A thiolase)	0.343	-1.543	0.000	0.000
68	205364_at	ACOX2	acyl-Coenzyme A oxidase 2, branched chain	0.344	-1.540	0.001	0.010
69	204607_at	HMGCS2	3-hydroxy-3-methylglutaryl-Coenzyme A synthase 2 (mitochondrial)	0.346	-1.530	0.002	0.010
70	213553_x_at	APOC1	apolipoprotein C-I	0.346	-1.530	0.000	0.000
71	206605_at	P11	26 serine protease	0.347	-1.526	0.000	0.000
72	201791_s_at	DHCR7	7-dehydrocholesterol reductase	0.353	-1.500	0.000	0.000
73	209800_at	KRT16	keratin 16 (focal non-epidermolytic palmoplantar keratoderma)	0.356	-1.492	0.006	0.030
74	220067_at	SPTBN5	spectrin, beta, non-erythrocytic 5	0.356	-1.492	0.000	0.000
75	37966_at	PARVB	parvin, beta	0.359	-1.480	0.000	0.000
76	220197_at	ATP6V0A4	ATPase, H+ transporting, lysosomal V0 subunit a4	0.360	-1.475	0.001	0.010
77	202275_at	G6PD	glucose-6-phosphate dehydrogenase	0.362	-1.465	0.000	0.000
78	209605_at	TST	thiosulfate sulfurtransferase (rhodanese)	0.362	-1.465	0.000	0.000
80	221604_s_at	PEX16	peroxisomal biogenesis factor 16	0.365	-1.453	0.000	0.000
81	209498_at	CEACAM1	carcinoembryonic antigen-related cell adhesion molecule 1 (biliary glycoprotein)	0.366	-1.452	0.000	0.000
82	212707_s_at	RASA4	RAS p21 protein activator 4	0.368	-1.443	0.000	0.000
83	210130_s_at	TM7SF2	transmembrane 7 superfamily member 2	0.370	-1.435	0.000	0.000
84	212276_at	LPIN1	lipin 1	0.370	-1.434	0.000	0.000
85	202025_x_at	ACAA1	acetyl-Coenzyme A acyltransferase 1 (peroxisomal 3-oxoacyl-Coenzyme A thiolase)	0.371	-1.431	0.000	0.000

	Probe	Symbol	Description	FCH-Scalp-NL-N	log2FCH-Scalp-NL-N	p-Scalp-NL-N	FDR-Scalp-NL-N
86	210521_s_at	FETUB	fetuin B	0.371	-1.432	0.000	0.000
87	206466_at	ACSBG1	acyl-CoA synthetase bubblegum family member 1	0.373	-1.424	0.008	0.040
88	220233_at	FBXO17	F-box protein 17	0.379	-1.399	0.001	0.000
89	200979_at	MAP3K15	mitogen-activated protein kinase kinase kinase 15	0.380	-1.394	0.000	0.000
90	209279_s_at	NSDHL	NAD(P) dependent steroid dehydrogenase-like	0.380	-1.397	0.000	0.000
91	214681_at	GK	glycerol kinase	0.382	-1.388	0.001	0.000
92	215184_at	DAPK2	death-associated protein kinase 2	0.383	-1.385	0.000	0.000
94	206869_at	CHAD	chondroadherin	0.384	-1.381	0.000	0.000
95	220751_s_at	C5orf4	chromosome 5 open reading frame 4	0.384	-1.380	0.000	0.000
96	221222_s_at	C1orf56	chromosome 1 open reading frame 56	0.385	-1.376	0.000	0.000
97	201790_s_at	DHCR7	7-dehydrocholesterol reductase	0.386	-1.373	0.000	0.000
98	206514_s_at	CYP4F3	cytochrome P450, family 4, subfamily F, polypeptide 3	0.386	-1.374	0.000	0.000
99	211663_x_at	PTGDS	prostaglandin D2 synthase 21kDa (brain)	0.386	-1.373	0.000	0.000
100	212187_x_at	PTGDS	prostaglandin D2 synthase 21kDa (brain)	0.386	-1.372	0.006	0.030
101	214001_x_at	RPS10	ribosomal protein S10	0.388	-1.367	0.001	0.010
102	215082_at	ELOVL5	ELOVL family member 5, elongation of long chain fatty acids (FEN1/Elo2, SUR4/Elo3-like, yeast)	0.388	-1.365	0.001	0.000
103	218922_s_at	LASS4	LAG1 homolog, ceramide synthase 4	0.388	-1.367	0.000	0.000
104	214549_x_at	SPRR1A	small proline-rich protein 1A	0.389	-1.364	0.000	0.000
105	222011_s_at	ACAT2	acetyl-Coenzyme A acetyltransferase 2 (acetoacetyl Coenzyme A thiolase)	0.389	-1.360	0.000	0.000
106	206723_s_at	EDG4	endothelial differentiation, lysophosphatidic acid G-protein-coupled receptor, 4	0.390	-1.360	0.000	0.000
107	210082_at	ABCA4	ATP-binding cassette, sub-family A (ABC1), member 4	0.392	-1.350	0.006	0.030
108	201275_at	FDPS	farnesyl diphosphate synthase (farnesyl pyrophosphate synthetase, dimethylallyltranstransferase, geranyltranstransferase)	0.395	-1.341	0.000	0.000
109	218476_at	POMT1	protein-O-mannosyltransferase 1	0.396	-1.337	0.000	0.000
110	220357_s_at	SGK2	serum/glucocorticoid regulated kinase 2	0.398	-1.328	0.000	0.000
111	205232_s_at	PAFAH2	platelet-activating factor acetylhydrolase 2, 40kDa	0.399	-1.325	0.000	0.000
112	221614_s_at	RPH3AL	rabphilin 3A-like (without C2 domains)	0.399	-1.326	0.000	0.000
113	211564_s_at	PDLIM4	PDZ and LIM domain 4	0.406	-1.301	0.000	0.000
114	218322_s_at	ACSL5	acyl-CoA synthetase long-chain family member 5	0.408	-1.292	0.000	0.000
115	201171_at	ATP6V0E1	ATPase, H+ transporting, lysosomal 9kDa, V0 subunit e1	0.410	-1.287	0.000	0.000
116	48030_i_at	C5orf4	chromosome 5 open reading frame 4	0.410	-1.286	0.001	0.000
117	221009_s_at	ANGPTL4	angiopoietin-like 4	0.411	-1.284	0.001	0.010
118	207254_at	SLC15A1	solute carrier family 15 (oligopeptide transporter), member 1	0.412	-1.279	0.000	0.000
119	221545_x_at	MED16	mediator complex subunit 16	0.412	-1.279	0.000	0.000
120	221810_at	RAB15	RAB15, member RAS onocogene family	0.412	-1.280	0.000	0.000
121	204067_at	SUOX	sulfite oxidase	0.413	-1.276	0.000	0.000
122	203798_s_at	VSNL1	visinin-like 1	0.414	-1.271	0.001	0.010
123	221848_at	ZGPAT	zinc finger, CCCH-type with G patch domain	0.414	-1.272	0.000	0.000
124	211695_x_at	MUC1	mucin 1, cell surface associated	0.416	-1.265	0.000	0.000
125	212281_s_at	TMEM97	transmembrane protein 97	0.416	-1.267	0.006	0.040
126	205233_s_at	PAFAH2	platelet-activating factor acetylhydrolase 2, 40kDa	0.418	-1.259	0.000	0.000
127	204687_at	DKFZP564O0823	DKFZP564O0823 protein	0.419	-1.254	0.007	0.040
128	205918_at	SLC4A3	solute carrier family 4, anion exchanger, member 3	0.420	-1.251	0.000	0.000
129	218840_s_at	NADSYN1	NAD synthetase 1	0.420	-1.250	0.000	0.000
130	205627_at	CDA	cytidine deaminase	0.421	-1.248	0.001	0.010
131	218739_at	ABHD5	abhydrolase domain containing 5	0.424	-1.237	0.000	0.000
132	205464_at	SCNN1B	sodium channel, nonvoltage-gated 1, beta (Liddle syndrome)	0.425	-1.234	0.001	0.010
133	219389_at	SUSD4	sushi domain containing 4	0.425	-1.234	0.001	0.010
134	208998_at	UCP2	uncoupling protein 2 (mitochondrial, proton carrier)	0.430	-1.217	0.000	0.000
135	218507_at	HIG2	hypoxia-inducible protein 2	0.430	-1.216	0.002	0.010
136	203189_s_at	NDUFS8	NADH dehydrogenase (ubiquinone) Fe-S protein 8, 23kDa (NADH-coenzyme Q reductase)	0.431	-1.214	0.000	0.000
137	205864_at	SLC7A4	solute carrier family 7 (cationic amino acid transporter, y+ system), member 4	0.433	-1.207	0.000	0.000
139	220675_s_at	PNPLA3	patatin-like phospholipase domain containing 3	0.434	-1.206	0.004	0.020
140	209016_s_at	KRT7	keratin 7	0.435	-1.201	0.008	0.040
141	204638_at	ACP5	acid phosphatase 5, tartrate resistant	0.437	-1.194	0.000	0.000
143	209792_s_at	KLK10	kallikrein-related peptidase 10	0.438	-1.190	0.001	0.010
145	219359_at	ATHL1	ATH1, acid trehalase-like 1 (yeast)	0.439	-1.187	0.000	0.000
146	202740_at	ACY1	aminoacylase 1	0.442	-1.178	0.000	0.000
147	218608_at	ATP13A2	ATPase type 13A2	0.442	-1.178	0.005	0.030
148	202067_s_at	LDLR	low density lipoprotein receptor (familial hypercholesterolemia)	0.443	-1.176	0.007	0.040
149	202030_at	BCKDK	branched chain ketoacid dehydrogenase kinase	0.445	-1.170	0.000	0.000
151	205221_at	HGD	homogentisate 1,2-dioxygenase (homogentisate oxidase)	0.445	-1.167	0.004	0.030
152	204669_s_at	RNF24	ring finger protein 24	0.447	-1.162	0.000	0.000
153	222155_s_at	GPR172A	G protein-coupled receptor 172A	0.447	-1.162	0.000	0.000
154	206515_at	CYP4F3	cytochrome P450, family 4, subfamily F, polypeptide 3	0.448	-1.159	0.001	0.010
155	218664_at	MECR	mitochondrial trans-2-enoyl-CoA reductase	0.448	-1.160	0.000	0.000
156	219752_at	RASAL1	RAS protein activator like 1 (GAP1 like)	0.448	-1.158	0.000	0.000
157	202003_s_at	ACAA2	acetyl-Coenzyme A acyltransferase 2 (mitochondrial 3-oxoacyl-Coenzyme A thiolase)	0.449	-1.156	0.003	0.020
158	203382_s_at	APOE	apolipoprotein E	0.449	-1.156	0.002	0.010
159	203515_s_at	PMVK	phosphomevalonate kinase	0.449	-1.154	0.000	0.000
160	203821_at	HBEGF	heparin-binding EGF-like growth factor	0.449	-1.155	0.003	0.020
163	209919_x_at	GGT1	gamma-glutamyltransferase 1	0.449	-1.155	0.000	0.000
164	219076_s_at	PXMP2	peroxisomal membrane protein 2, 22kDa	0.449	-1.155	0.001	0.000
166	211748_x_at	PTGDS	prostaglandin D2 synthase 21kDa (brain)	0.450	-1.153	0.009	0.040
167	215966_x_at	GK3P	glycerol kinase 3 pseudogene	0.451	-1.148	0.002	0.010
168	220425_x_at	ROPN1	ropporin, rhophilin associated protein 1	0.451	-1.149	0.005	0.030
169	204981_at	SLC22A18	solute carrier family 22 (organic cation transporter), member 18	0.452	-1.146	0.000	0.000
170	205031_at	EFNB3	ephrin-B3	0.453	-1.143	0.000	0.000
171	204139_x_at	MZF1	myeloid zinc finger 1	0.455	-1.136	0.000	0.000
172	217973_at	DCXR	dicarbonyl/L-xylulose reductase	0.455	-1.136	0.000	0.000
173	211056_s_at	SRD5A1	steroid-5-alpha-reductase, alpha polypeptide 1 (3-oxo-5 alpha-steroid delta 4-dehydrogenase alpha 1)	0.456	-1.133	0.003	0.020
174	202735_at	EBP	emopamil binding protein (sterol isomerase)	0.457	-1.131	0.000	0.000
175	208700_s_at	TKT	transketolase (Wernicke-Korsakoff syndrome)	0.458	-1.126	0.000	0.000
176	214041_x_at	RPL37A	ribosomal protein L37a	0.458	-1.126	0.003	0.020
177	37117_at	ARHGAP8	Rho GTPase activating protein 8	0.458	-1.127	0.002	0.020
178	206628_at	SLC5A1	solute carrier family 5 (sodium/glucose cotransporter), member 1	0.459	-1.122	0.004	0.030

	Probe	Symbol	Description	FCH-Scalp-NL-N	log2FCH-Scalp-NL-N	p-Scalp-NL-N	FDR-Scalp-NL-N
179	202525_at	PRSS8	protease, serine, 8	0.460	-1.120	0.000	0.000
180	206754_s_at	CYP2B6	cytochrome P450, family 2, subfamily B, polypeptide 6	0.460	-1.121	0.000	0.000
181	213935_at	ABHD5	abhydrolase domain containing 5	0.460	-1.121	0.002	0.020
182	214023_x_at	TUBB2B	tubulin, beta 2B	0.460	-1.120	0.000	0.000
183	218921_at	SIGIRR	single immunoglobulin and toll-interleukin 1 receptor (TIR) domain	0.460	-1.119	0.000	0.000
184	218795_at	ACP6	acid phosphatase 6, lysophosphatidic	0.461	-1.118	0.001	0.010
185	210336_x_at	MZF1	myeloid zinc finger 1	0.462	-1.115	0.000	0.000
186	45653_at	KCTD13	potassium channel tetramerisation domain containing 13	0.462	-1.115	0.006	0.030
187	203458_at	SPR	sepiapterin reductase (7,8-dihydrobiopterin:NADP+ oxidoreductase)	0.463	-1.111	0.000	0.000
188	208534_s_at	RASA4	RAS p21 protein activator 4	0.463	-1.112	0.002	0.010
189	212212_s_at	INTS1	integrator complex subunit 1	0.463	-1.110	0.000	0.000
190	38710_at	OTUB1	OTU domain, ubiquitin aldehyde binding 1	0.463	-1.109	0.002	0.020
191	215387_x_at	GPC6	glypican 6	0.464	-1.107	0.000	0.000
192	206709_x_at	GPT	glutamic-pyruvate transaminase (alanine aminotransferase)	0.465	-1.106	0.000	0.000
193	213497_at	ABTB2	ankyrin repeat and BTB (POZ) domain containing 2	0.465	-1.105	0.000	0.000
194	46142_at	TMEM112	transmembrane protein 112	0.465	-1.104	0.000	0.000
195	213796_at	SPRR1A	small proline-rich protein 1A	0.467	-1.097	0.001	0.010
196	218272_at	FLJ20699	hypothetical protein FLJ20699	0.467	-1.098	0.000	0.000
197	209514_s_at	RAB27A	RAB27A, member RAS oncogene family	0.468	-1.096	0.006	0.040
198	209617_s_at	CTNND2	catenin (cadherin-associated protein), delta 2 (neural plakophilin-related arm-repeat protein)	0.468	-1.095	0.002	0.010
199	210653_s_at	BCKDHB	branched chain keto acid dehydrogenase E1, beta polypeptide (maple syrup urine disease)	0.469	-1.093	0.005	0.030
200	220486_x_at	TMEM164	transmembrane protein 164	0.469	-1.092	0.000	0.000
201	202856_s_at	SLC16A3	solute carrier family 16, member 3 (monocarboxylic acid transporter 4)	0.470	-1.088	0.000	0.000
202	215785_s_at	CYFIP2	cytoplasmic FMR1 interacting protein 2	0.471	-1.086	0.003	0.020
203	204730_at	RIMS3	regulating synaptic membrane exocytosis 3	0.472	-1.082	0.000	0.000
204	203119_at	CCDC86	coiled-coil domain containing 86	0.473	-1.081	0.000	0.000
205	208284_x_at	GGT1	gamma-glutamyltransferase 1	0.473	-1.080	0.000	0.000
207	222057_at	NOL12	nucleolar protein 12	0.473	-1.081	0.000	0.000
208	201490_s_at	PPIF	peptidylprolyl isomerase F (cyclophilin F)	0.474	-1.078	0.008	0.040
209	211105_s_at	NFATC1	nuclear factor of activated T-cells, cytoplasmic, calcineurin-dependent 1	0.474	-1.077	0.005	0.030
210	32836_at	AGPAT1	1-acylglycerol-3-phosphate O-acyltransferase 1 (lysophosphatidic acid acyltransferase, alpha)	0.474	-1.077	0.006	0.030
211	203652_at	MAP3K11	mitogen-activated protein kinase kinase kinase 11	0.475	-1.073	0.002	0.020
212	202793_at	MBOAT5	membrane bound O-acyltransferase domain containing 5	0.477	-1.068	0.000	0.000
213	204401_at	KCNN4	potassium intermediate/small conductance calcium-activated channel, subfamily N, member 4	0.477	-1.069	0.003	0.020
214	204546_at	KIAA0513	KIAA0513	0.478	-1.066	0.000	0.000
215	218368_s_at	TNFRSF12A	tumor necrosis factor receptor superfamily, member 12A	0.478	-1.064	0.005	0.030
216	219188_s_at	MACROD1	MACRO domain containing 1	0.478	-1.064	0.001	0.010
217	209695_at	PTP4A3	protein tyrosine phosphatase type IVA, member 3	0.480	-1.058	0.000	0.000
218	220782_x_at	KLK12	kallikrein-related peptidase 12	0.480	-1.060	0.000	0.000
219	203777_s_at	RPS6KB2	ribosomal protein S6 kinase, 70kDa, polypeptide 2	0.481	-1.055	0.000	0.000
220	204875_s_at	GMDS	GDP-mannose 4,6-dehydratase	0.481	-1.055	0.000	0.000
221	35147_at	MCF2L	MCF.2 cell line derived transforming sequence-like	0.481	-1.056	0.000	0.000
222	203702_s_at	TTLL4	tubulin tyrosine ligase-like family, member 4	0.482	-1.053	0.002	0.010
223	209262_s_at	NR2F6	nuclear receptor subfamily 2, group F, member 6	0.482	-1.054	0.000	0.000
224	219689_at	SEMA3G	sema domain, immunoglobulin domain (Ig), short basic domain, secreted, (semaphorin) 3G	0.482	-1.052	0.006	0.040
225	220734_s_at	LOC727825	hypothetical protein LOC727825	0.484	-1.046	0.000	0.000
226	222356_at	TBL1Y	transducin (beta)-like 1Y-linked	0.484	-1.046	0.000	0.000
227	200789_at	ECH1	enoyl Coenzyme A hydratase 1, peroxisomal	0.485	-1.044	0.000	0.000
228	213787_s_at	TBC1D25	TBC1 domain family, member 25	0.485	-1.044	0.001	0.010
229	212694_s_at	PCCB	propionyl Coenzyme A carboxylase, beta polypeptide	0.487	-1.038	0.000	0.000
230	215243_s_at	GJB3	gap junction protein, beta 3, 31kDa	0.488	-1.036	0.000	0.000
231	215535_s_at	AGPAT1	1-acylglycerol-3-phosphate O-acyltransferase 1 (lysophosphatidic acid acyltransferase, alpha)	0.488	-1.036	0.001	0.000
232	221551_x_at	ST6GALNAC4	ST6 (alpha-N-acetyl-neuraminyl-2,3-beta-galactosyl-1,3)-N-acetylgalactosaminide alpha-2,6-sialyltransferase 4	0.488	-1.036	0.000	0.000
233	208699_x_at	TKT	transketolase (Wernicke-Korsakoff syndrome)	0.489	-1.031	0.000	0.000
234	209618_at	CTNND2	catenin (cadherin-associated protein), delta 2 (neural plakophilin-related arm-repeat protein)	0.489	-1.031	0.000	0.000
235	207081_s_at	PI4KA	phosphatidylinositol 4-kinase, catalytic, alpha	0.490	-1.030	0.000	0.000
236	213273_at	ODZ4	odz, odd Oz/ten-m homolog 4 (Drosophila)	0.490	-1.028	0.000	0.000
237	219044_at	THNSL2	threonine synthase-like 2 (S. cerevisiae)	0.490	-1.029	0.002	0.010
238	204247_s_at	CDK5	cyclin-dependent kinase 5	0.491	-1.027	0.000	0.000
239	210720_s_at	APBA2BP	amyloid beta (A4) precursor protein-binding, family A, member 2 binding protein	0.491	-1.026	0.000	0.000
240	221938_x_at	MED16	mediator complex subunit 16	0.491	-1.027	0.000	0.000
241	41160_at	MBD3	methyl-CpG binding domain protein 3	0.491	-1.026	0.000	0.000
242	204263_s_at	CPT2	carnitine palmitoyltransferase II	0.493	-1.021	0.000	0.000
243	212274_at	LPIN1	lipin 1	0.494	-1.019	0.001	0.010
244	209017_s_at	LONP1	lon peptidase 1, mitochondrial	0.495	-1.015	0.000	0.000
246	211071_s_at	MLLT11	myeloid/lymphoid or mixed-lineage leukemia (trithorax homolog, Drosophila); translocated to, 11	0.495	-1.015	0.008	0.040
247	215489_x_at	HOMER3	homer homolog 3 (Drosophila)	0.497	-1.008	0.000	0.000
248	204212_at	ACOT8	acyl-CoA thioesterase 8	0.498	-1.007	0.000	0.000
249	212611_at	DTX4	deltex 4 homolog (Drosophila)	0.498	-1.005	0.000	0.000
250	204343_at	ABCA3	ATP-binding cassette, sub-family A (ABC1), member 3	0.499	-1.003	0.000	0.000

Table H. Gene Set Variation Analysis (GSVA): 'Epidermal Biology' and 'Cells' groups of gene sets.

		Skin				Scalp				Scalp vs Skin normalized by tissue			
Group	Gene set	LS vs NL		NL vs Normal		LS vs NL		NL vs Normal		LS		NL	
		logFCH	FDR	logFCH	FDR	logFCH	FDR	logFCH	FDR	logFCH	FDR	logFCH	FDR
Epidermal Biology	Epidermal Differentiation Complex (EDC)	2.26	<0.001	3.12	0.061	2.07	<0.001	-1.4	0.061	-4.71	<0.001	-4.52	<0.001
	Cornified Envelope (CE)	1.56	0.001	2.11	0.001	1.56	0.001	-0.85	0.272	-2.95	<0.001	-2.95	0.001
	EDC-CE	2.73	<0.001	3.75	<0.001	2.59	<0.001	-1.62	0.085	-5.51	<0.001	-5.37	0.001
	Basal vs upper epidermis	-6.07	<0.001	-5.38	<0.001	-3.98	<0.001	2.3	0.157	9.77	<0.001	7.68	<0.001
	Skin pigmentation	-0.07	0.898	2.79	<0.001	0.22	0.705	2.11	0.016	-0.39	0.692	-0.68	0.534
	Skin pigmentation (cell surf. recep.)	-0.92	<0.001	0.68	0.022	-0.68	0.002	0.26	0.525	-0.17	0.681	-0.41	0.378
	Skin pigmentation (TFs)	-1.29	<0.001	1.65	0.001	-0.56	0.121	1.63	0.009	0.71	0.293	-0.02	0.978
	Skin pigmentation (transp. & uptake)	2.28	<0.001	1.86	0.001	1.92	<0.001	0.32	0.686	-1.91	0.013	-1.55	0.065
	Skin pigmentation (mel. synthesis)	-1.27	0.010	1.08	0.099	-1.15	0.023	2.04	0.015	1.07	0.254	0.96	0.368
Cells	Treg	0.70	0.271	0.96	0.262	1.77	0.006	1.71	0.099	1.81	0.124	0.74	0.576
	T cells	8.07	<0.001	6.55	<0.001	7.25	<0.001	-0.74	0.686	-8.11	<0.001	-7.29	<0.001
	CD4+ T cells	0.34	0.384	-1.11	0.034	0.90	0.024	1.24	0.057	2.91	<0.001	2.35	0.003
	PBMC	6.69	<0.001	3.78	0.001	6.34	<0.001	-1.34	0.355	-5.47	<0.001	-5.12	0.003
	Macrophages	-0.30	0.551	0.23	0.745	-1.02	0.041	0.07	0.947	-0.88	0.345	-0.16	0.873
	Macrophages (LPS)	6.29	<0.001	4.15	<0.001	5.13	<0.001	2.42	0.075	-2.88	0.055	-1.72	0.332
	Monocytes	0.99	0.081	3.16	<0.001	0.78	0.186	1.76	0.064	-1.61	0.129	-1.40	0.241
	Mature DCs	1.66	<0.001	1.05	0.050	1.88	<0.001	-1.77	0.011	-2.59	<0.001	-2.82	0.001
	Immature DCs	-0.01	0.976	-0.15	0.821	0.35	0.513	-2.61	0.002	-2.09	0.023	-2.46	0.014
	Inflammatory Myeloid DCs	1.48	0.094	-0.26	0.821	0.94	0.319	2.07	0.157	1.79	0.288	2.33	0.201
	BDCA(-) DCs LS vs NL	0.95	0.003	-1.47	0.001	0.72	0.031	1.45	0.010	2.68	<0.001	2.92	<0.001
	Keratinocytes	4.62	<0.001	7.40	<0.001	5.41	<0.001	-1.16	0.596	-7.77	<0.001	-8.56	<0.001
	Melanocytes	-4.08	<0.001	-2.72	0.002	-3.20	<0.001	0.59	0.632	4.19	<0.001	3.31	0.010
	Fibroblasts	-9.15	<0.001	-6.18	0.006	-7.60	<0.001	4.34	0.120	12.07	<0.001	10.52	0.002

Table I. Gene Set Variation Analysis (GSVA): 'Immune response' group of gene sets.

		Skin				Scalp				Scalp vs Skin normalized by tissue			
Group	Gene set	LS vs NL		NL vs Normal		LS vs NL		NL vs Normal		LS		NL	
		logFCH	FDR	logFCH	FDR	logFCH	FDR	logFCH	FDR	logFCH	FDR	logFCH	FDR
Immune Response	Immune Genes	5.99	<0.001	1.98	0.060	5.54	<0.001	0.89	0.544	-1.54	0.302	-1.09	0.524
	Th1	4.57	<0.001	0.93	0.212	3.61	<0.001	1.58	0.079	-0.31	0.769	0.65	0.574
	Th1 (specific)	4.65	<0.001	1.44	0.041	3.68	<0.001	1.73	0.053	-0.68	0.510	0.29	0.790
	Th2	2.63	<0.001	0.28	0.728	2.59	<0.001	-0.13	0.901	-0.45	0.680	-0.42	0.729
	Th2 (specific)	1.96	<0.001	1.04	0.039	1.81	<0.001	-0.03	0.967	-1.22	0.080	-1.07	0.163
	Th17	4.07	<0.001	3.91	<0.001	3.32	<0.001	-1.05	0.157	-5.70	<0.001	-4.96	<0.001
	Th17 (specific)	3.96	<0.001	2.49	<0.001	3.20	<0.001	-0.47	0.525	-3.73	<0.001	-2.96	<0.001
	Th22	1.62	<0.001	2.51	<0.001	1.40	<0.001	-0.73	0.132	-3.46	<0.001	-3.24	<0.001
	Th22 and IL-22	1.89	<0.001	1.73	0.001	1.98	<0.001	0.1	0.901	-1.54	0.027	-1.63	0.031
	KC IFNA (WR Swindell)	8.07	<0.001	3.46	0.038	8.35	<0.001	2.68	0.193	-0.51	0.837	-0.78	0.763
	KC IFNG (WR Swindell)	5.97	<0.001	4.39	0.003	6.07	<0.001	6.12	<0.001	1.82	0.372	1.73	0.459
	KC IFNA	8.23	<0.001	1.08	0.413	6.33	<0.001	3.04	0.059	0.06	0.969	1.96	0.347
	KC IFNG	9.81	<0.001	7.74	<0.001	10.14	<0.001	0.63	0.763	-6.78	0.001	-7.11	0.002
	KC IL-1	6.85	<0.001	4.79	<0.001	6.96	<0.001	-1.12	0.319	-5.80	<0.001	-5.91	<0.001
	KC IL-4 (WR Swindell)	-1.95	<0.001	-0.46	0.519	-0.52	0.326	0.22	0.826	2.11	0.024	0.68	0.531
	KC IL-13 (WR Swindell)	0.04	0.915	-1.12	0.017	0.23	0.532	1.05	0.066	2.37	<0.001	2.18	0.002
	KC IL-17	7.86	<0.001	3.25	0.002	6.02	<0.001	-0.59	0.686	-5.67	<0.001	-3.84	0.011
	KC IL-17 (Illumina)	6.75	<0.001	3.91	<0.001	5.72	<0.001	-0.62	0.630	-5.56	<0.001	-4.53	<0.001
	KC IL-17 (WR Swindell)	10.64	<0.001	3.69	0.004	7.27	<0.001	-0.83	0.646	-7.89	<0.001	-4.52	0.017
	KC IL-17 (not TNFa)	3.78	<0.001	0.24	0.750	2.39	<0.001	-0.79	0.391	-2.42	0.013	-1.03	0.368
	KC IL-17 and TNFa	6.96	<0.001	3.83	<0.001	5.70	<0.001	-0.15	0.901	-5.25	<0.001	-3.99	0.003
	KC IL-22	4.20	<0.001	1.40	0.040	3.46	<0.001	-0.61	0.525	-2.76	0.003	-2.02	0.047

		Skin				Scalp				Scalp vs Skin normalized by tissue			
Group	Gene set	LS vs NL		NL vs Normal		LS vs NL		NL vs Normal		LS		NL	
		logFCH	FDR	logFCH	FDR	logFCH	FDR	logFCH	FDR	logFCH	FDR	logFCH	FDR
(cont.)	KC Additive IL17&IL-22	6.96	*<0.001*	2.94	*<0.001*	5.53	*<0.001*	0.34	0.741	-4.02	*<0.001*	-2.60	*0.018*
	KC Additive IL17&TNFa	11.32	*<0.001*	6.05	*<0.001*	8.71	*<0.001*	-1.24	0.489	-9.90	*<0.001*	-7.29	*<0.001*
	KC Additive TNFa&IL-22	1.73	*<0.001*	2.93	*<0.001*	1.12	*<0.001*	-0.03	0.944	-3.58	*<0.001*	-2.97	*<0.001*
	KC Synergistic IL17&IL-22	6.37	*<0.001*	2.21	*0.004*	5.67	*<0.001*	0.58	0.596	-2.33	*0.025*	-1.63	*0.160*
	KC Synergistic IL17&TNFa	6.26	*<0.001*	4.37	*<0.001*	5.79	*<0.001*	-1.03	0.336	-5.87	*<0.001*	-5.40	*<0.001*
	Eosinophils IL-13	2.95	*<0.001*	2.11	*0.016*	2.37	*<0.001*	2.58	*0.018*	-0.11	0.922	0.47	0.730
	Macrophages IFNG	8.56	*<0.001*	6.42	*<0.001*	7.24	*<0.001*	3.52	*0.034*	-4.22	*0.019*	-2.90	0.150
	Macrophages IFNG (LPS)	8.46	*<0.001*	5.88	*<0.001*	7.44	*<0.001*	4.01	*0.018*	-2.88	0.124	-1.87	0.385
	Macrophages IL-4	-0.28	0.645	-0.27	0.750	-0.10	0.884	0.49	0.682	0.94	0.414	0.75	*0.556*
	Macrophages TNFa	0.58	0.146	2.84	*<0.001*	0.92	*0.025*	0.95	0.157	-1.55	*0.033*	-1.89	*0.017*
	Monocytes IL-17	3.06	0.235	4.99	0.146	2.62	0.326	9.96	*0.020*	4.53	0.350	4.96	*0.368*
	Myeloid DCs IL-17	-2.72	*<0.001*	-1.91	*0.004*	-2.55	*<0.001*	1.93	*0.019*	4.00	<0.001	3.84	*<0.001*
	iDCs IL-17	0.30	0.464	0.25	0.655	0.01	0.990	-0.36	0.632	-0.91	0.222	-0.61	*0.477*
	Fibroblasts IL-4	-0.91	*0.009*	-0.29	0.558	-0.82	*0.022*	0.25	0.698	0.63	0.345	0.54	0.477
	Fibroblasts IFNG	5.09	*<0.001*	2.55	0.061	6.23	*<0.001*	3.54	*0.038*	2.14	0.269	1.00	*0.646*
	Fibroblasts IL-17	1.06	*0.018*	-0.59	0.342	0.93	*0.043*	-0.07	0.940	0.38	0.664	0.51	0.591
	Fibroblasts IL-22	0.29	0.693	2.84	*0.001*	0.74	0.320	-1.67	0.157	-4.05	*0.002*	-4.51	*0.002*
	KC Atopic Dermatitis IL-17	5.84	*<0.001*	4.69	*<0.001*	4.66	*<0.001*	-0.56	0.634	-0.06	*<0.001*	-5.24	*<0.001*
	KC Atopic Dermatitis IL-22	2.56	*<0.001*	1.75	*<0.001*	2.50	*<0.001*	-0.47	0.326	-2.28	*<0.001*	-2.22	*<0.001*
	KC Atopic Dermatitis IL-17 LS^NL	4.90	*<0.001*	4.58	*<0.001*	4.06	*<0.001*	-0.51	0.630	-5.93	*<0.001*	-5.09	*<0.001*
	KC Atopic Dermatitis IL-22 LS^NL	1.85	*<0.001*	0.34	0.342	1.98	*<0.001*	-0.41	0.366	-0.62	0.206	-0.75	*0.161*
	Rheumatoid Arthritis IFNG	8.37	*<0.001*	7.39	*<0.001*	9.43	*<0.001*	-2.52	0.292	-8.84	*0.001*	-9.91	*0.001*
	Rheumatoid Arthritis IL-17	15.31	*<0.001*	6.91	*<0.001*	11.18	*<0.001*	-2.54	0.282	-13.58	*<0.001*	-9.45	*0.001*
	Rheumatoid Arthritis IL-22	3.58	*<0.001*	2.16	*<0.001*	3.39	*<0.001*	-1.00	0.142	-3.35	*<0.001*	-3.15	*<0.001*

Table J. Gene Set Variation Analysis (GSVA): 'Psoriasis' group of gene sets.

		Skin				Scalp				Scalp vs Skin normalized by tissue			
Group	Gene set	LS vs NL		NL vs Normal		LS vs NL		NL vs Normal		LS		NL	
		logFCH	FDR	logFCH	FDR	logFCH	FDR	logFCH	FDR	logFCH	FDR	logFCH	FDR
PsO studies	PsO NGS	28.90	*<0.001*	15.27	*<0.001*	22.22	*<0.001*	-2.72	0.513	-24.68	*<0.001*	-18.00	*<0.001*
	PsO MAD5	35.54	*<0.001*	17.67	*<0.001*	27.85	*<0.001*	0.09	0.952	-27.27	*<0.001*	-17.58	*0.003*
	PsO MAD3	37.45	*<0.001*	16.51	*<0.001*	28.18	*<0.001*	-0.31	0.952	-26.0.9	*<0.001*	-16.82	*0.005*
	PsO Centocore	35.93	*<0.001*	17.96	*<0.001*	27.49	*<0.001*	0.38	0.947	-26.02	*<0.001*	-17.58	*0.006*
	PsO (Y. Yao)	36.35	*<0.001*	13.80	*<0.001*	27.30	*<0.001*	-0.83	0.901	-23.69	*<0.001*	-14.64	*0.009*
	PsO (M. Suarez-Fariñas)	35.87	*<0.001*	16.83	*<0.001*	25.57	*<0.001*	2.15	0.698	-24.99	*<0.001*	-14.68	*0.014*
	PsO (J.E. Gudjonsson) LSvsNL	32.78	*<0.001*	15.52	*<0.001*	24.49	*<0.001*	0.75	0.901	-23.06	*<0.001*	-14.77	*0.004*
	PsO (J.E. Gudjonsson) UP	31.42	*<0.001*	13.82	*<0.001*	23.32	*<0.001*	0.8	0.893	-21.13	*<0.001*	-13.03	*0.008*
	PsO (A.M. Bowcock)	18.67	*<0.001*	8.87	*<0.001*	13.67	*<0.001*	1.71	0.570	-12.16	*<0.001*	-7.16	*0.023*

Table K. Gene Set Variation Analysis (GSVA): 'Genetic regulation' group of gene sets.

		Skin				Scalp				Scalp vs Skin normalized by tissue			
Group	Gene set	LS vs NL		NL vs Normal		LS vs NL		NL vs Normal		LS		NL	
		logFCH	FDR	logFCH	FDR	logFCH	FDR	logFCH	FDR	logFCH	FDR	logFCH	FDR
Genetical regulation	Positive regulators	4.72	*<0.001*	3.89	*0.001*	5.03	*<0.001*	2.08	0.157	-1.51	0.372	-1.81	0.350
	Negative regulators	1.91	*<0.001*	-0.69	0.310	1.96	*<0.001*	1.64	0.052	2.38	*0.009*	2.34	*0.018*
	MIFT targets	-2.64	*<0.001*	1.50	*0.022*	-2.31	*<0.001*	0.64	0.478	-0.53	0.574	-0.87	0.406
	IL-17 targets (S. Gaffen)	4.26	*<0.001*	2.85	*<0.001*	3.60	*<0.001*	0.03	0.975	-3.48	*0.001*	-2.82	*0.014*
	Epigenetics changes LS vs NL	-2.61	*<0.001*	-2.13	*0.001*	-1.25	*0.007*	-0.53	0.525	2.97	*<0.001*	1.60	0.078
	Epigenetics changes NL vs N	-0.80	*0.003*	0.50	0.169	-0.16	0.569	-0.88	0.053	-0.73	0.141	-1.37	*0.010*
